# A phylogenetic analysis of the British flora sheds light on the evolutionary and ecological factors driving plant invasions

**DOI:** 10.1002/ece3.1274

**Published:** 2014-10-23

**Authors:** Junying Lim, Mick J Crawley, Natasha De Vere, Tim Rich, Vincent Savolainen

**Affiliations:** 1 Department of Life Sciences, Imperial College London, Silwood Park CampusAscot, SL5 7PY, UK; 2 National Botanic Gardens of WalesLlanarthne, Carmarthenshire, SA32 8HG, UK; 3 57 Aberdulais Road, Cardiff, CF14 2PH, UK; 4 Royal Botanic GardensKew, Richmond, TW9 3DS, UK

**Keywords:** British flora, Darwin's naturalization hypothesis, Ellenberg indicators, functional trait, invasive species, molecular phylogenetics

## Abstract

Darwin's naturalization hypothesis predicts that invasive species should perform better in their novel range in the absence of close relatives in the native flora due to reduced competition. Evidence from recent taxonomic and phylogenetic-based studies, however, is equivocal. We test Darwin's naturalization hypothesis at two different spatial scales using a fossil-dated molecular phylogenetic tree of the British native and alien flora (ca. 1600 species) and extensive, fine-scale survey data from the 1998 Countryside Survey. At both landscape and local scales, invasive species were neither significantly more nor less related to the native flora than their non-invasive alien counterparts. Species invasiveness was instead correlated with higher nitrogen and moisture preference, but not other life history traits such as life-form and height. We argue that invasive species spread in Britain is hence more likely determined by changes in land use and other anthropogenic factors, rather than evolutionary history. *Synthesis*. The transition from non-invasive to invasive is not related to phylogenetic distinctiveness to the native community, but instead to their environmental preferences. Therefore, combating biological invasions in the Britain and other industrialized countries need entirely different strategies than in more natural environments.

## Introduction

Invasive species are considered one of the major threats to ecosystems worldwide (Sala 2000) and have the propensity to alter ecosystems fundamentally through their effects on native biodiversity (Powell et al. 2011; van Hengstum et al. 2014), ecosystem processes (Vilà et al. 2011) and ultimately the services they provide (Pejchar and Mooney 2009). Some invasive species have also been implicated in major economic losses in agriculture and forestry, in addition to the costs associated with controlling and managing their impacts after they have established (Pimentel et al. 2005). Given these economic and environmental impacts, there has been tremendous interest in identifying potential problematic introductions in the early stages of invasion, or even screen species before its introduction. As such, research over the past few decades has focused on understanding why certain alien species become invasive, whereas others do not (Rejmánek and Richardson 1996).

One approach has been to focus on predicting species invasiveness either by comparing the functional traits or performance of invasive species to that of either non-invasive alien or native species (Williamson and Fitter 1996; Kolar and Lodge 2001; Daehler 2003; van Kleunen et al. 2010). For example, van Kleunen et al. (2010), in a meta-analysis of 117 studies, found that invasive species tended to be associated with higher values across various performance-related traits, compared with non-invasive species. Similarly, some invasive-native comparisons have found significant differences in allocation to reproduction (Hawkes 2007), height and seed size (Crawley et al. 1996; Ordonez et al. 2010). Although it is hard to dismiss that successful invaders are characterized by certain life history traits, results have been highly idiosyncratic (Colautti et al. 2006) and appear to be context dependent (Daehler 2003).

Some researchers have attempted to predict patterns of invasiveness on the basis of shared evolutionary history (e.g., Daehler 2001) with the native flora. One of the earliest theories, first proposed by Darwin (Darwin 1859), suggests that because closely related taxa are more similar in ecological traits (e.g., soil requirements, shade tolerance) than distantly related taxa, they are more likely to face strong competition with natives (Elton 1958) or share their natural enemies (Keane and Crawley 2002), the so-called Darwin's naturalization hypothesis (Daehler 2001). Darwin's naturalization hypothesis hence predicts distantly related invaders to be more successful at invading novel environments. The opposite pattern, however, has also been suggested. High relatedness to the native taxa may allow an invader to be better preadapted to the invaded environment (Darwin 1859; Duncan and Williams 2002).

Evidence for Darwin's naturalization hypothesis to date, however, has been largely mixed. Previous studies have either found that invasion success was associated with distantly related invaders (Rejmánek 1996; Strauss et al. 2006; Schaefer et al. 2011; Park and Potter 2013), closely related ones (Daehler 2001; Duncan and Williams 2002; Cadotte et al. 2009; Ricotta et al. 2010), or found no pattern (Lambdon and Hulme 2006; Lambdon 2008). It has, however, been increasingly argued that such conflicting results may be because observed phylogenetic patterns are highly dependent on the spatial scale within which they are considered (Procheş et al. 2008; Diez et al. 2008; Cadotte et al. 2009; Thuiller et al. 2010). For example, Cavender-Bares et al. (2006) found that species within a community appeared more closely related when compared with species pools of greater size. This is in part because competitive and abiotic filtering processes in community assembly may be dominant at different spatial scales (Cavender-Bares et al. 2006; Swenson et al. 2006). Similarly, the predictions of Darwin's naturalization hypothesis may be more applicable at smaller spatial scales where competitive interactions dominate. Conversely, at larger spatial scales, closely related invasive species may be more likely to co-occur with native assemblages due to similar broad-scale environmental preferences brought about by shared evolutionary history. To date, only a few studies have addressed issues of scale when testing Darwin's naturalization hypothesis (Cadotte et al. 2009; Schaefer et al. 2011; Carboni et al. 2013).

Furthermore, while both trait-based and phylogenetic approaches have their merits, few studies take into account both the characteristics of the invader and its relatedness to the native communities (Carboni et al. 2013; Park and Potter 2013). For example, Schaefer et al. (2011) found that a combination of both traits and phylogenetic relatedness best predicted plant species invasiveness in the Azores. In addition, recent phylogenetic studies of Darwin's naturalization hypothesis are often limited in taxonomic scope (Strauss et al. 2006; Park and Potter 2013), and their generality is unclear.

Here, we built a comprehensive molecular phylogenetic tree of the alien and native flora of Britain, encompassing ca. 1600 species. We tested the generality of Darwin's naturalization hypothesis in the British flora, asking if non-invasive and invasive alien species differ in their phylogenetic relatedness to the native flora. We also asked whether such relatedness patterns change across spatial scales, using comprehensive fine-scale survey data across Britain. Finally, we tested the relative importance of phylogenetic relatedness and various ecological traits such as life-form, clonality, and Ellenberg indicator scores, in influencing species invasiveness.

## Materials and Methods

### Plant sampling and data

A list of the flora of the British Isles was adapted from a comprehensive inventory of species and traits of the British and Irish flora – PLANTATT (Hill et al. 2004). Hybrids and casual aliens were excluded from the study, while species complexes, aggregates, and subspecies were collapsed into single species. We focus our research on Britain, which represents a naturally defined island area – excluding Northern Ireland.

We used data at different scales. Small-scale vegetation data were obtained from the 1998 Countryside Survey (http://www.countrysidesurvey.org.uk/). The Countryside Survey consists of vegetation plot surveys conducted within stratified, randomly chosen 1 km squares, and designed to representatively cover all landscape types in Great Britain (see Smart et al. 2003 for more details on sampling methodology). Plots were also randomized within each 1 × 1 km square to reduce spatial clustering and sampled a range of landscape features and plant communities: stream and river banks, road verges, hedgerows, fields, and unenclosed land. Taxa within each plot were identified to species level. We used plot data from linear features (1 × 10 m) and areal plots (2 × 2 m). Where plot types were sampled in a nested fashion, only the smallest, least inclusive nest (2 × 2 m) was used. Uncertain species records were excluded, after which only plots with at least one native and one alien species were included (see Fig. S1). In total, 5541 non-native species occurrences across 3614 plots (21% of all plots) were included in the analyses. It is worth noting, however, that urban habitats were intentionally under-represented by the Countryside Survey; sampling design avoided 1 km squares with >75% built land (Smart et al. 2003).

The classification of invasiveness status is a difficult task (Richardson et al. 2000; Colautti and MacIsaac 2004; Valéry et al. 2008), and studies often adopt either a geographic (Richardson et al. 2000) or an ecological impact criterion (Davis and Thompson 2000). Here, we divided the non-native species into “invasive aliens” and “non-invasive aliens” based on their ecological impact and relative abundance in the recipient communities that they invade (see Table S1). Because there is no unified protocol for quantifying the impact of alien plants, any “impact criterion” is bound to be context dependent. Here, we based our classification following Stace and Crawley (2015). While some argue that the geographic spread of self-sustaining populations beyond their original point of introduction may be a more objective measure, we argue that an “impact criterion” is important in the context of local community dynamics. Also, our classification is highly consistent with relative changes in hectad level (10 × 10 km) occupancy across the United Kingdom for the two groups, as such it incorporate a geographic component in addition to the ecological impact described by Stace and Crawley (2015). Using PLANTATT (Hill et al. 2004) data on alien species’ “Change Index” (Telfer et al. 2002) between two periods – 1930–1960 and 1987–1999 – invasive alien species under our definition have increased more greatly in range during the intervening period than non-invasive alien species (Wilcoxon rank-sum test, *W* = 4659.5, *P* < 0.001). The change index was only calculated for species recorded in both time periods and hence excludes the most recent introductions. One caveat, however, is that because botanical records have improved, some of the apparent expansion in ranges of alien taxa, particularly of recent arrivals, may be due in part to changes in recorder effort, although such changes in recorder effort should affect both groups similarly. Nevertheless, we believe that our classification is robust and hence capture both aspects (“impact” and “geographic”) of their dynamics.

We also subdivided alien species into archaeophytes and neophytes (species introduced before, and after, approximately 1500 A.D., respectively) based on PLANTATT (Hill et al. 2004), reflecting the history of plant invasion in Europe. Archaeophytes are often associated with old crops (e.g., cereals) introduced with Neolithic agriculture, while neophytes were typically introduced following the discovery of the New World as more recent agricultural and horticultural introductions (Pyšek et al. 2005). In Britain, these two alien groups are often distinguished by a variety and combination of paleobotanical, archaeological and historical evidence (Preston et al. 2004). Because of their introduction histories and longer residence time, most archaeophytes are thought to be more integrated into native plant communities and thus more constrained by environmental filters than neophytes (Ricotta et al. 2009).

### DNA extraction and sequencing

To achieve good phylogenetic resolution and internal support, a combination of two plastid loci were used – the maturase K gene *matK* and the large subunit of the ribulose 1,5-bisphosphate carboxylase/oxygenase gene (*rbcL*). A combination of portions of *rbcL* and *matK* has been recommended as the plant barcode based on assessments of recoverability, sequence quality and discriminatory power among species (CBOL Plant Working Group 2009). Furthermore, large-scale phylogenies based on *rbcL* have proved successful for recovering angiosperm relationships (Chase et al. 1993), while *matK* has the advantage of evolving faster than that of *rbcL* and providing finer resolution (Hilu et al. 2003).

A combination of previously published and DNA sequences produced here was used for phylogenetic construction. 1421 (82.1%) and 1362 (78.7%) sequences were obtained from GenBank/EBI for *rbcL* and *matK,* respectively. Together, these sequences comprise 1489 (86.1%) of the 1729 species considered (see Table S1). A large proportion of sequences were from a recently completed barcoding project for the native Welsh flora (de Vere et al. 2012). Of the remaining 240 taxa not in GenBank, we acquired leaf samples for 123 species (Table S2). We also acquired samples for missing sequences in 51 species (species with either *rbcL* or *matK* available in GenBank, and therefore requiring further sequencing; Table S2).

DNA extraction from leaf material was performed using the CTAB method (Doyle and Doyle 1987). The two loci were subsequently amplified and sequenced using standard protocols described by the Plant Working Group for the Consortium for the Barcode of Life (CBOL Plant Working Group 2009). We amplified *rbcL* in two overlapping fragments using the primer pairs: *rbcL*-1F, *rbcL*-700R; and *rbcL*-600F, *rbcL*-1460R (Asmussen and Chase 2001); therefore, we sequenced the entire *rbcL* exon rather than just the portion recommended as the barcoding region. For *matK*, a pair of universal primers of the sequence were used to amplify the DNA barcoding region – *matK*-F-uni: 5′-ATT TTA CGA TCH ATT CAT TCM ATW TTT CC-3′ and *matK*-R-uni: 5′-AGT TYT ARC ACA AGA AAG TCG AAR TAT ATA-3′ (Schaefer et al. 2011). All DNA sequences are available in GenBank/EBI (http://www.ncbi.nlm.nih.gov/) (Table S2).

### Phylogenetic inference

Sequences were edited using Geneious pro 6.0 (Biomatters 2013). The *rbcL* and *matK* sequences were aligned separately using MAFFT (Katoh et al. 2005) and concatenated. maximum-likelihood (ML) tree searches were performed using RAxML-VI-HPC v7.0.4 (Stamatakis 2006) using 1000 bootstrap replicates, with two independent partitions corresponding to each locus and specifying a GTR-GAMMA model of nucleotide substitution as selected by jModeltest 2 (Darriba et al. 2012) on the basis of Akaike information criterion. Tree searches were also constrained at the family level based on the Angiosperm Phylogeny Group III (APG) classification (Angiosperm Phylogeny Group 2009), generated using Phylomatic (version 3; Phylomatic tree R20120829)(Webb and Donoghue 2005).

Due to the size of the data set, we were limited with regard to the methods that can be used to date the tree. We estimated divergence times in the phylogeny using nonparametric rate smoothing (Sanderson 1997) implemented in r8s (Sanderson 2003) on the best-scoring ML tree. We calibrated the tree by fixing the age of the eudicot crown group at 121 million years (mya), which corresponds to the appearance of tricolpate pollen grains characteristic of the clade (Drinnan et al. 1994). Because of the large taxonomic scope and disparities in diversity across tracheophyte clades, we set an upper limit on the dates estimated for the tracheophytes and angiosperms, constraining the maximum age of the tracheophytes at 454 mya following Clarke et al. (2011) and constraining the age of the angiosperms crown group to be between 140 and 180 mya (Soltis et al. 2008). We calibrated the tree further with four minimum age constraints: monilophytes, seed plants (spermatophytes), Nymphaeales, and the node subtending the Cucurbitales and Fagales clades (see Table S3). The dated tree is available from TreeBASE (http://treebase.org/; accession number 15105).

### Ecological traits

Trait data for plant primary life-form, height (cm), clonality, and Ellenberg indicator values for light (*L*), moisture (*F*), soil fertility (*N*), soil pH (*R*), and salt tolerance (*S*) were obtained from PLANTATT (Hill et al. 2004). While Ellenberg indicator values (Ellenberg et al. 1992) are not strictly plant traits, they can be interpreted to reflect broad environmental or habitat preferences (e.g., Thompson and McCarthy 2008). Primary life-form data were based on Raunkiaer's life-form categories. Plant height was log-transformed to improve normality, and species were grouped by their ability to spread clonally or not.

### Spatial distribution and invasiveness analyses

To test for spatial congruence between non-invasive and invasive alien species, we used atlas data at the hectad scale (10 × 10 km) across Britain (Preston et al. 2002). We calculated the Spearman's rank correlation between species richness of the two alien groups. We used Dutilleul's test (Dutilleul et al. 1993) which evaluates the “effective degrees of freedom” after taking into account the spatial autocorrelation of both samples have been taken into account. To reduce the influence of uninvaded cells, we omitted grid cells where neither non-invasive nor invasive species were recorded. Spatial covariance was incorporated using the centroids of each hectad. To assess which hectads have a higher or lower richness of invasive species relative to non-invasive alien species richness, we calculated the residuals from a loess regression of invasive alien species richness on non-invasive alien species richness.

Using Fritz and Purvis (2010) measure of phylogenetic signal for binary traits (D), we evaluated the signal strength of invasiveness in the phylogeny. *D* allows us to compare observed phylogenetic patterns of invasiveness against null scenarios where invasiveness is randomly assigned across the tips of the phylogeny and where invasiveness is simulated under a Brownian threshold model (Felsenstein 2005). Values of *D* < 1 indicate that invasiveness was more phylogenetically clustered than expected than random, whereas values of *D* > 1 indicate that invasiveness was more phylogenetically dispersed than random. We calculated *D* for invasiveness among the naturalized aliens, among archaeophytes and among neophytes, each time with 10,000 randomizations.

Using the dated tree, we also quantified the evolutionary relatedness of each alien taxon to the native flora using two metrics: the phylogenetic nearest neighbor distance (PNND) and mean phylogenetic distance (MPD). PNND was calculated by summing up the total intervening branch length between each alien species and the native taxa to which it is most closely related in the phylogeny, whereas MPD was calculated as the mean pairwise phylogenetic distance between the alien species and all native taxa. We performed these calculations at two spatial scales: at the country scale and the local scale (Countryside Survey plots).

Linear models for PNND and MPD were used to test whether phylogenetic relatedness to the native flora differed between invasive alien and non-invasive alien species. For local-scale analyses, we instead used linear mixed effects models, fitting invasiveness as a fixed effect. To account for the nonindependence of PNND and MPD of alien species among plots within the same sampled 1 × 1 km square, square identity was fitted as a nested random effect within species identity (i.e*., PNND or MPD ˜ invasiveness + (1 | species/square)*).

We modeled invasiveness of alien taxa at the country scale as a binary response variable using a generalized linear model with a binomial error structure, with treewide PNND, alien status group (archaeophyte or neophyte), differences in ecological variables between the alien taxa and its most closely related native, as well as absolute ecological trait values as explanatory variables. For continuous traits, the trait value of the alien species was subtracted from the trait value of the most closely related native species. For categorical traits, a value of 1 was assigned if both alien and the closest native species had different trait classes, whereas a value of 0 was assigned if both taxa shared a trait class. Ellenberg indicator values and plant height were treated as continuous variables, while life-form and clonality were treated as categorical variables in our model. To obtain uncertainty estimates for parameters, a model-averaging approach was adopted (Burnham and Anderson 2002). However, because of the large number of variables assessed and data constraints, we only fitted all possible candidate models with combinations of up to five variables to avoid model over-fitting. Furthermore, because we expect the phylogenetic distance metrics as well as relative trait difference calculations to be sensitive to phylogenetic topology and branch lengths, we evaluated model sensitivity by repeating the analysis using a second recently published, dated, ultrametric phylogenetic tree of the European flora, hereafter referred to as the DAPHNE phylogeny (Durka and Michalski 2012). The topology of the DAPHNE phylogeny is similarly based upon the backbone family phylogeny of the APG III, but constructed by manually pruning partial phylogenetic subtrees from 518 recent studies onto this backbone. We did not perform the same analysis at the local scale as our invasiveness classification was at the species level and recorded non-native contribution to cover in Countryside Survey plots was generally low (Maskell et al. 2006) and hence inadequate to determine local-scale invasiveness.

All analyses and modeling were implemented in R v3.01 (R Core Team 2013). Mixed effect models were implemented using the “lmer” function in the *lme4* package (Bates and Maechler 2013). Model averaging was implemented using the *MuMIn* package (v 1.95, (“MuMIn: R package for multi-model inference 2013)). Dutilleul's test was implemented using “modified t-test” in the *SpatialPack* (v 0.2, Osorio et al. 2013). *D* was calculated using the “phylo.d” function in the *caper* package (Orme et al. 2012).

## Results

We obtained sequence information (either generated de novo or from GenBank) for 1612 species (non-invasive alien: 274, invasive alien: 89, native: 1249), just over 93% of the total British taxa considered in PLANTATT (Hill et al. 2004)(non-invasive alien: 93.1%, invasive alien: 96.7%, native: 93.0%).

**Figure 1:**
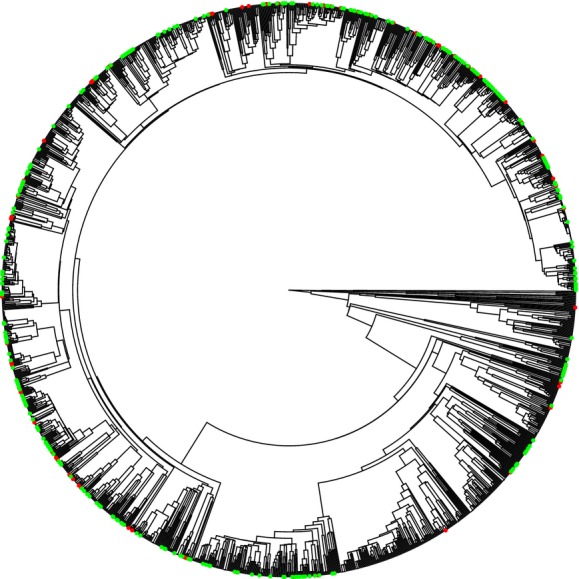
Time-calibrated phylogeny of the British flora (1249 native, 274 non-invasive, and 89 invasive). Tip labels are not shown for clarity. Alien species are highlighted (red = invasive, green = non-invasive).

The combined phylogenetic matrix consisted of 4692 aligned nucleotides. Most nodes in the tree are well supported, although a few genera were not recovered as monophyletic groups in our tree (Fig. 1).

**Figure 2:**
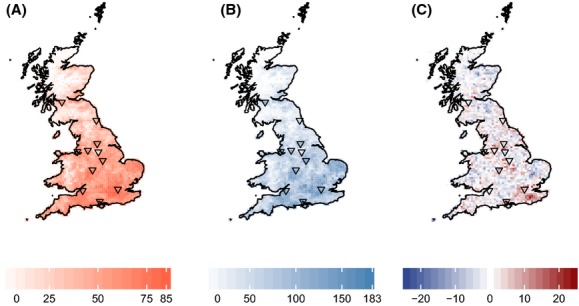
Species richness of invasive (A) and non-invasive (B) alien species across Britain based on atlas data (Preston et al. 2002) at the hectad scale. (C) Residuals from a loess (local second-degree polynomial) regression (smoothing parameter, *α* = 0.75; pseudo-*R*^2^ = 0.951) of invasive and non-invasive species richness. Positive residuals are indicated in red, while negative residuals are shown in blue. Triangles indicate the 11 densest cities.

There was a high amount of spatial congruence between invasive alien and non-invasive alien species distributions (Spearman's rank correlation = 0.946, *F* = 45.3, df = 1, 5.33, *P* < 0.001; Fig. 2A and B). Of the 2814 hectad (10 × 10 km) grid cells across Britain, only 64 were uninvaded by either non-invasive or invasive aliens. Both invasive and non-invasive alien species show a latitudinal gradient in species diversity and appear to be associated with areas of high urbanization (Fig. 2A and B). Patterns of invasive species richness relative to non-invasive species richness do not show a latitudinal gradient, but highlight strong regional “hot spots” (Fig. 2C).

**Figure 3:**
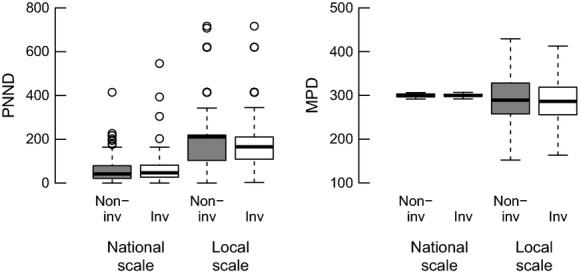
Phylogenetic nearest neighbor distance (PNND; left) and mean phylogenetic distance (MPD, outliers not shown for clarity; right) of alien species to the native flora at the national scale (89 invasive and 274 non-invasive) and local scale (99 invasive and 61 non-invasive) (in millions of years; based on the time-calibrated phylogenetic tree of alien species across Britain).

Invasiveness appears to have a nonrandom phylogenetic signal among the neophytes and all naturalized aliens, while a signal was slightly weaker among the archaeophytes (Table 1, Fig. 1). Across the British flora, PNND (*t* = 1.206, df = 1, 361, *P* = 0.23) and MPD (*t* = −0.544, df = 1, 361, *P* = 0.59) are not significantly different between invasive and non-invasive aliens (Fig. 3). Our results were qualitatively the same using the DAPHNE phylogeny (PNND: *t* = 1.475, df = 1, 344, *P* = 0.14; MPD: *t* = −0.623, df = 1, 344, *P* = 0.53)(Fig. S2). The Countryside Survey plots analyzed comprised a total of 5541 occurrences of 160 non-native species (99 invasive and 61 non-invasive) across 3614 plots. Most plots (68%) only contained one non-native species (mean = 1.53, max = 12). Note that due to differences in species coverage in phylogenies, analyses using the DAPHNE phylogeny considered 4829 occurrences of 154 alien species (95 non-invasive and 59 invasive) across 3341 plots. At the local scale, no significant difference in PNND and MPD between invasive and non-invasive species was found (Table 2, Fig. 3).

**Table 1:** Phylogenetic signal of invasiveness. P_random_ and P_Brownian_ are *P*-values showing whether *D* is significantly different from expected from random (*D* = 1) or from Brownian expectation (*D* = 0), respectively. Number of randomizations = 10,000

	No. of invasives	No. of non-invasives	*D*	P_random_	P_Brownian_
Among naturalized aliens	89	274	0.75	0.001	0
Among neophytes	61	167	0.76	0.018	0
Among archaeophytes	28	107	0.78	0.091	0.001

**Table 2:** Mixed effects models of PNND and MPD across Countryside Survey (CS) plots fitted using restricted maximum likelihood (REML). Estimates of mean PNND and MPD (±standard errors) are reported. PNND and MPD were calculated using the time-calibrated phylogeny of this study and the DAPHNE phylogeny (Durka and Michalski 2012)

Caefd	Time-calibrated phylogeny (*n* = 5541)	DAPHNE phylogeny (*n* = 4829)
PNND		
Invasive	180.86 ± 18.31	172.58 ± 23.15
Non-invasive	201.56 ± 18.31	203.51 ± 23.15
MPD		
Invasive	291.79 ± 14.07	262.92 ± 18.54
Non-invasive	306.64 ± 14.07	280.36 ± 18.54

Although invasives are neither more nor less phylogenetically related to the native flora than non-invasive aliens, at the UK scale, invasiveness is significantly positive correlated with soil fertility (Ellenberg *N*) and moisture preferences (Ellenberg *F*) (Table 3). PNND, alien status (archaeophyte or neophyte), relative trait differences, and life history traits, such as plant height, various life-form types, and clonality were not significantly associated with invasiveness (Table 3). Model averaging with models containing PNND and relative trait differences calculated using the DAPHNE supertree were qualitatively similar (Table S4).

**Table 3:** Model-averaging results for generalized linear models with invasiveness as a binary trait for the entire UK. *N* = 363 alien species (274 non-invasive, 89 invasive). Coefficients are averages from the full set of candidate models. SE = standard error; CI = 95% confidence intervals; relative variable importance is the sum of Akaike weights across all models that included that variable (Burnham and Anderson 2002)

Variable	Model averaged coefficient	Adjusted SE	Lower CI	Upper CI	Relative Importance
Ellenberg *F*	0.303	1.506	0.121	0.484	0.93^1^
Ellenberg *N*	0.263	0.117	0.061	0.466	0.85^1^
Ellenberg *L*	−0.229	0.092	−0.460	0.001	0.49
Ellenberg *S* (Difference)	−0.212	0.137	−0.483	0.057	0.36
Clonality	0.377	0.305	−0.223	0.976	0.22
Log height (difference)	0.181	0.178	−0.167	0.529	0.18
Alien group (archaeophyte/neophytes)	−0.242	0.299	−0.828	0.345	0.16
Log height	0.074	0.108	−0.138	0.286	0.15
Life-form (difference)	0.196	0.269	−0.331	0.724	0.14
Ellenberg *R* (Difference)	0.092	0.130	−0.164	0.348	0.14
Ellenberg *L* (Difference)	0.073	0.122	−0.167	0.313	0.13
Clonality (Difference)	−0.17	0.293	−0.744	0.404	0.13
Ellenberg *N* (Difference)	0.056	0.101	−0.141	0.253	0.13
Ellenberg *F* (Difference)	0.049	0.094	−0.136	0.234	0.13
Ellenberg *S*	0.132	0.301	−0.458	0.723	0.13
Ellenberg *R*	0.037	0.159	−0.274	0.348	0.12
PNND	−0.000953	0.00230	−0.005	0.003	0.12
Primary life-form:					
Bulbous geophytes	−1.81	1.21	−4.179	0.546	0.07
Nonbulbous geophytes	−0.352	0.840	−1.999	1.294	
Hemicryptophytes	−0.150	0.615	−1.355	1.056	
Hydrophyte	2.56	1.36	−0.109	5.232	
Phanerophyte	0.220	0.706	−1.163	1.604	
Nanophanerophyte	0.342	0.768	−1.162	1.847	
Therophyte	−0.507	0.612	−1.706	0.695	

1 A parameter was considered significant if its 95% confidence interval (CI) of the parameter estimate does not include 0.

## Discussion

While it has been argued that the lack of consensus in support for Darwin's naturalization hypothesis may be attributed to differences in spatial scale (Procheş et al. 2008; Thuiller et al. 2010), a consistent scale-dependent pattern has received mixed empirical support (Davies et al. 2010; Schaefer et al. 2011; Carboni et al. 2013). Schaefer et al. (2011) found that invasive species tend to be less closely related to the native Azorean flora than non-invasive species, but significance was lost at the smallest scale. In contrast, Carboni et al. (2013) demonstrated that while most invaders of Mediterranean coastal marsh plant communities were less related to their nearest native relative at the finest sampling resolution, the same pattern was not found at larger scales, with some non-native species actually being more closely related to the native communities than expected by chance.

Here, we find no evidence that the presence of closely related native species influences species invasiveness (using two different phylogenetic relatedness metrics). This was also true at finer scales (4–10 m^2^) across the British countryside, where competition is expected to dominate (Swenson et al. 2006). While Countryside Survey plots were biased against urban sites, focusing on natural and semi-natural habitats, competition should be strongest in these habitats due to a smaller impact of disturbance (Burke and Grime 1996; Davis et al. 2000).

One possible explanation for the lack of phylogenetic signal is that, while widely assumed, phylogenetic relatedness may not strongly reflect the outcome of competitive interactions (Cahill et al. 2008; Kunstler et al. 2012; Bennett et al. 2013) or patterns of co-occurrence (Narwani et al. 2013). Moreover, patterns of niche conservatism have been hypothesized to be scale dependent, with niche traits that determine coexistence within habitats being less conserved and labile (Silvertown et al. 2006a,b).

In addition, while biotic resistance may have some impact on invader performance and establishment success, competitive processes rarely lead to exclusion of the invader and that biotic interactions may instead simply constrain the abundance of invasive species (Levine et al. 2004). While we do not test this at the local scale, this is a challenging problem. Indeed, most studies, ours included, implicitly assume that biotic constraints from the native community should lead to contrasting patterns of co-occurrence of invasive and non-invasive alien species across local communities. However, given that we are dealing with aliens at the spread and impacts phase, relatedness would rather generate differences in the abundance and not co-occurrence patterns. Furthermore, it is difficult to differentiate the effects of competitive exclusion from other processes such as dispersal limitation or local differences in propagule pressure (Veltman et al. 1996; Lockwood et al. 2005; Simberloff 2009), on the absence of an invader.

Together, our results indicate that tests of Darwin's naturalization hypothesis may be confounded in two principal ways: phylogenetic relatedness may not relate to trait similarity and/or does not capture competitive interactions at macroscale. Patterns of phylogenetic relatedness between native invaders may not always reflect the outcome of competitive processes on co-occurrence patterns when dealing with postestablishment communities. Hence, treewide comparisons based on relatedness in the phylogeny alone may have limited ability to detect a signal of biotic resistance from competition once an alien has already been established. Further, local-scale patterns of co-occurrence may have limited power to differentiate invasive alien and non-invasive alien performance and hence underestimate nearest neighbor phylogenetic distances.

Although we do not find a phylogenetic pattern consistent with Darwin's naturalization hypothesis, we also do not find invasive species to be more closely related to the native flora than their non-invasive counterparts at the landscape scale. Instead, we find that invasive species differ from their non-invasive counterparts in their abiotic preferences. Such differences in habitat preferences may explain the non-random phylogenetic signal in species invasiveness. For example, Thompson et al. (1995) investigated increasing alien and native plants of four northwest European countries and found that successful invaders were strongly habitat dependent. While our study does not test for phylogenetic niche conservatism in the naturalized aliens, there is evidence that Ellenberg indicator values (Prinzing et al. 2001) and habitat-determining traits (Silvertown et al. 2006a) are more evolutionarily conserved. Together, this suggests that invasion success may be less dependent upon sharing similar traits with the native flora, but more determined by landscape-level changes in abiotic environment. For example, the high spatial congruence in invasive and non-invasive alien species richness suggests that the same large-scale abiotic filters and anthropogenic factors are constraining their distributions (Fig. 2A and B). However, “hot spots” of high invasive alien species richness relative to non-invasive alien species richness highlights the importance of local changes in environmental conditions in mediating invasion success (Fig. 2C).

In particular, invasive aliens in Britain appear to have a preference for more fertile and wetter conditions compared with non-invasive aliens. Invasive alien species performance has long been associated with nutrient-rich conditions where changes in resource availability may alter competitive hierarchies in local communities (Burke and Grime 1996; Davis and Pelsor 2001; Daehler 2003). While the association of invasiveness with higher moisture may be partly driven by aquatic plants which tend to be highly invasive (e.g., *Elodea nuttallii*, *E. canadensis*, and *Azolla filiculoides*), our results are consistent with other studies that looked at plot scale trait associations as well as changes in species composition across habitats in Britain (Smart et al. 2003; Braithwaite et al. 2006; Maskell et al. 2006; Norton et al. 2012). Invaded communities in the Britain are significantly associated with native communities of higher soil fertility (Maskell et al. 2006) and nutrient-rich wet habitats (Maskell et al. 2008).

Furthermore, there is some evidence that landscape-scale environmental changes in Britain may be favouring invasive species with high nutrient and moisture preferences. Decreases in species richness has been observed in infertile habitats such as calcareous grasslands (Braithwaite et al. 2006; Maskell et al. 2010), whereas changes in plant community composition suggest increased nutrient availability across both upland and lowland landscapes (Smart et al. 2003). Moreover, there have been increase in species preferring wetter conditions across all vegetation types in the United Kingdom due to large-scale changes in rainfall regime (Norton et al. 2012).

In conclusion, our study calls for further evaluation of the role of phylogenetic relatedness in predicting invasiveness (Lambdon and Hulme 2006; Mitchell et al. 2006) especially in highly disturbed environments. Darwin's original hypothesis may apply to pristine, naturally invaded environments or may be restricted to certain spatial scales, whereas these relationships may be masked in highly man-modified landscapes such as in Britain. Although we do not dismiss the important role of native species composition on the invasibility of local communities (Crawley et al. 1999; Levine et al. 2004), anthropogenic drivers such as eutrophication, urbanization or land-use changes that alter habitat-level attributes more likely have had greater influence on the spread of invasive species in Britain than competitive interactions. Therefore, combating biological invasions in the Britain and other industrialized countries may need entirely different strategies than in more natural environments.

## Supporting Information

**Figure S1.** Distribution of Countryside Survey plots. **Figure S2.** Phylogenetic nearest neighbor distance and mean phylogenetic distance of invasive and non-invasive alien species based on the DAPHNE phylogeny. **Table S1.** Species included in our study, GenBank/EBI accession numbers for rbcL and matK, and species invasiveness classifications. **Table S2.** Specimen sources and sequences generated for this study. **Table S3.** Age calibration points used for divergence time estimation. **Table S4.** Model-averaging results for species invasiveness using the DAPHNE phylogeny.
