# Supplementary material for: A phylogenetic analysis of the British flora sheds light on the evolutionary and ecological factors driving plant invasions
Source: Ecol Evol. 2014 Oct 23;4(22):4258–69. doi: 10.1002/ece3.1274 (PMC4267865; doi:10.1002/ece3.1274)
Supplement: Supplementary file 1 [file ece30004-4258-SD1.docx]

**Supplementary Information: Junying Lim, Mick J Crawley, Natasha De Vere, Tim Rich, Vincent Savolainen**

**Fig. S1**: Distribution of the 3,614 Countryside Survey plots included in this study across Britain. Colour scale represents the number of plots considered within each hectad.

**Fig. S2**: Mean phylogenetic nearest neighbor distance (PNND; left) and mean phylogenetic distance (MPD, outliers not shown for clarity; right)(in millions of years; based on the DAPHNE supertree) of invasive (red) and non-invasive (green) alien species across Britain (n = 346), and at the local scale (n = 154).

**Table S1**: Species included in our study, Genbank/EBI accession numbers for *rbcL* and *matK* and invasive species classifications. T.S. = this study (Supplementary Table 2). Status: N = native, AN = neophyte, AR = archaeophyte [1]. Invasive: Inv = high invasive impact, Non-inv = low invasive impact, Nat = native. WCA are invasive species classified under the Wildlife and Countryside Act 1981

| \|  \| Taxon.name \| matK_accession \| rbcL_accession \| NS \| WCA \| Inv? \| \| --- \| --- \| --- \| --- \| --- \| --- \| --- \| \| 1 \| Acaena novae-zelandiae \| T.S. \| T.S. \| AN \|  \| Non-inv \| \| 2 \| Acer campestre \| JN894032 \| DQ978399 \| N \|  \| Nat \| \| 3 \| Acer platanoides \| AJ438788.1 \| DQ978424.1 \| AN \|  \| Inv \| \| 4 \| Acer pseudoplatanus \| HM850604.1 \| HM849739.1 \| AN \|  \| Inv \| \| 5 \| Aceras anthropophorum \| \| T.S. \| N \|  \| Nat \| \| 6 \| Achillea millefolium \| EU385315.1 \| HM849740 \| N \|  \| Nat \| \| 7 \| Achillea ptarmica \| JN895745.1 \| JN891887.1 \| N \|  \| Nat \| \| 8 \| Acorus calamus \| AJ879453.1 \| AJ879453.1 \| AN \|  \| Inv \| \| 9 \| Actaea spicata \| JQ033440.1 \| JQ033709.1 \| N \|  \| Nat \| \| 10 \| Adiantum capillus-veneris \| NC_004766.1 \| NC_004766.1 \| N \|  \| Nat \| \| 11 \| Adonis annua \| JN895412 \| JN891273.1 \| AR \|  \| Non-inv \| \| 12 \| Adoxa moschatellina \| AF446900.1 \| AF446930.1 \| N \|  \| Nat \| \| 13 \| Aegopodium podagraria \| JN894400.1 \| U50220.1 \| AR \|  \| Inv \| \| 14 \| Aesculus hippocastanum \| AY724266.1 \| HE963308.1 \| AN \|  \| Non-inv \| \| 15 \| Agrimonia eupatoria \| JN893958.1 \| HM849744.1 \| N \|  \| Nat \| \| 16 \| Agrimonia procera \| JN894283.1 \| JN891532.1 \| N \|  \| Nat \| \| 17 \| Agrostis canina \| FJ231115.1 \| JN893786.1 \| N \|  \| Nat \| \| 18 \| Agrostis capillaris \| FJ231112.1 \| AY395527.1 \| N \|  \| Nat \| \| 19 \| Agrostis curtisii \| JN894587.1 \| JN891530.1 \| N \|  \| Nat \| \| 20 \| Agrostis gigantea \| JN895346.1 \| JN893047.1 \| AR \|  \| Non-inv \| \| 21 \| Agrostis stolonifera \| NC_008591.1 \| NC_008591.1 \| N \|  \| Nat \| \| 22 \| Agrostis vinealis \| FJ231113.1 \| JN893780.1 \| N \|  \| Nat \| \| 23 \| Aira caryophyllea \| DQ786878.1 \| HM849751.1 \| N \|  \| Nat \| \| 24 \| Aira praecox \| AM234540.1 \| HM849752.1 \| N \|  \| Nat \| \| 25 \| Ajuga reptans \| JN894214.1 \| U32163.1 \| N \|  \| Nat \| \| 26 \| Alchemilla acutiloba \| T.S. \| T.S. \| N \|  \| Nat \| \| 27 \| Alchemilla alpina \| T.S. \| T.S. \| N \|  \| Nat \| \| 28 \| Alchemilla filicaulis \| JN894290.1 \| JN891526.1 \| N \|  \| Nat \| \| 29 \| Alchemilla glabra \| JN894582.1 \| JN892118.1 \| N \|  \| Nat \| \| 30 \| Alchemilla glaucescens \| T.S. \| T.S. \| N \|  \| Nat \| \| 31 \| Alchemilla glomerulans \| T.S. \| T.S. \| N \|  \| Nat \| \| 32 \| Alchemilla micans \| T.S. \| T.S. \| N \|  \| Nat \| \| 33 \| Alchemilla minima \| T.S. \| T.S. \| NE \|  \| Nat \| \| 34 \| Alchemilla mollis \| T.S. \| U06792.1 \| AN \|  \| Non-inv \| \| 35 \| Alchemilla monticola \| \| T.S. \| N \|  \| Nat \| \| 36 \| Alchemilla wichurae \| T.S. \| T.S. \| N \|  \| Nat \| \| 37 \| Alchemilla xanthochlora \| JN895007.1 \| JN890788.1 \| N \|  \| Nat \| \| 38 \| Alisma gramineum \| JF781067.1 \| JF781041.1 \| N \|  \| Nat \| \| 39 \| Alisma lanceolatum \| HM850585.1 \| HM849753.1 \| N \|  \| Nat \| \| 40 \| Alisma plantago-aquatica \| JF781065.1 \| JN890986.1 \| N \|  \| Nat \| \| 41 \| Alliaria petiolata \| AF144363.1 \| JQ933212.1 \| N \|  \| Nat \| \| 42 \| Allium ampeloprasum \| JN893883.1 \| HM849754.1 \| AR \|  \| Non-inv \| \| 43 \| Allium carinatum \| \| HE963312.1 \| AN \|  \| Non-inv \| \| 44 \| Allium oleraceum \| JQ412191.1 \| JQ412309.1 \| N \|  \| Nat \| \| 45 \| Allium paradoxum \| \| T.S. \| AN \| 1 \| Non-inv \| \| 46 \| Allium roseum \| \| T.S. \| AN \|  \| Non-inv \| \| 47 \| Allium schoenoprasum \| \| JN890785.1 \| N \|  \| Nat \| \| 48 \| Allium scorodoprasum \| T.S. \| T.S. \| N \|  \| Nat \| \| 49 \| Allium triquetrum \| HM850503.1 \| HM849755.1 \| AN \| 1 \| Inv \| \| 50 \| Allium ursinum \| JN896208.1 \| HF572822.1 \| N \|  \| Nat \| \| 51 \| Allium vineale \| HM850504.1 \| HM849756.1 \| N \|  \| Nat \| \| 52 \| Alnus glutinosa \| HQ600562.1 \| EU644678.1 \| N \|  \| Nat \| \| 53 \| Alnus incana \| HM850705.1 \| HM849757.1 \| AN \|  \| Non-inv \| \| 54 \| Alopecurus aequalis \| JN894037.1 \| HQ600469.1 \| N \|  \| Nat \| \| 55 \| Alopecurus borealis \| JN966072.1 \| JN965222.1 \| N \|  \| Nat \| \| 56 \| Alopecurus bulbosus \| JN894201.1 \| JN891006.1 \| N \|  \| Nat \| \| 57 \| Alopecurus geniculatus \| JN894200.1 \| HM849758.1 \| N \|  \| Nat \| \| 58 \| Alopecurus myosuroides \| FN908046.1 \| JN891007.1 \| AR \|  \| Inv \| \| 59 \| Alopecurus pratensis \| EU434293.1 \| AY395528.1 \| N \|  \| Nat \| \| 60 \| Althaea hirsuta \| EU346794.1 \| T.S. \| AN \|  \| Non-inv \| \| 61 \| Althaea officinalis \| EU346765.1 \| JN891502.1 \| N \|  \| Nat \| \| 62 \| Amaranthus albus \| JF953137.1 \| JF940782.1 \| AN \|  \| Non-inv \| \| 63 \| Amaranthus retroflexus \| JF953149.1 \| JF940796.1 \| AN \|  \| Non-inv \| \| 64 \| Ammophila arenaria \| JN895834.1 \| JN893253.1 \| N \|  \| Nat \| \| 65 \| Amsinckia micrantha \| \| T.S. \| AN \|  \| Non-inv \| \| 66 \| Anacamptis pyramidalis \| JN895643.1 \| JN892999.1 \| N \|  \| Nat \| \| 67 \| Anagallis arvensis \| JN895655.1 \| HM849770.1 \| N \|  \| Nat \| \| 68 \| Anagallis minima \| JN894204.1 \| HM849772.1 \| N \|  \| Nat \| \| 69 \| Anagallis tenella \| JN893976.1 \| HM849773.1 \| N \|  \| Nat \| \| 70 \| Anaphalis margaritacea \| HM445632.1 \| HQ589951.1 \| AN \|  \| Non-inv \| \| 71 \| Anchusa arvensis \| EU599718.1 \| JN893638.1 \| AR \|  \| Non-inv \| \| 72 \| Andromeda polifolia \| AF124569.1 \| AF124572.1 \| N \|  \| Nat \| \| 73 \| Anemone nemorosa \| JN895407.1 \| JN893775.1 \| N \|  \| Nat \| \| 74 \| Angelica sylvestris \| DQ133783.1 \| DQ133798.1 \| N \|  \| Nat \| \| 75 \| Anisantha rigida \| HM850584.1 \| HQ600435.1 \| AN \|  \| Non-inv \| \| 76 \| Anisantha sterilis \| JN895072.1 \| AY836155.1 \| AR \|  \| Inv \| \| 77 \| Anogramma leptophylla \| \| AY168719.1 \| N \|  \| Nat \| \| 78 \| Antennaria dioica \| HM445620.1 \| JN891313.1 \| N \|  \| Nat \| \| 79 \| Anthemis arvensis \| HM850610.1 \| HM849778.1 \| AR \|  \| Non-inv \| \| 80 \| Anthemis cotula \| JN895749.1 \| HM849779.1 \| AR \|  \| Non-inv \| \| 81 \| Anthoxanthum odoratum \| DQ786884.1 \| AJ746256.1 \| N \|  \| Nat \| \| 82 \| Anthriscus caucalis \| JN895073.1 \| JN893703.1 \| N \|  \| Nat \| \| 83 \| Anthriscus sylvestris \| U58547.1 \| FJ395576.1 \| N \|  \| Nat \| \| 84 \| Anthyllis vulneraria \| JN894822.1 \| JN892206.1 \| N \|  \| Nat \| \| 85 \| Antirrhinum majus \| AF375189.1 \| GQ997015.1 \| AN \|  \| Non-inv \| \| 86 \| Apera interrupta \| \| EF125147.1 \| AN \|  \| Non-inv \| \| 87 \| Apera spica-venti \| AM234542.1 \| T.S. \| AR \|  \| Non-inv \| \| 88 \| Aphanes arvensis \| JN894468.1 \| JN892207.1 \| N \|  \| Nat \| \| 89 \| Aphanes australis \| JN895218.1 \| JN892408.1 \| N \|  \| Nat \| \| 90 \| Apium graveolens \| AJ429370.1 \| HM849783.1 \| N \|  \| Nat \| \| 91 \| Apium inundatum \| JN895622.1 \| JN892973.1 \| N \|  \| Nat \| \| 92 \| Apium nodiflorum \| JN895216.1 \| HM850050.1 \| N \|  \| Nat \| \| 93 \| Aquilegia vulgaris \| HM851055.1 \| HM849784.1 \| N \|  \| Nat \| \| 94 \| Arabidopsis thaliana \| NC_000932.1 \| NC_000932.1 \| N \|  \| Nat \| \| 95 \| Arabis alpina \| AF144329.1 \| JN965256.1 \| N \|  \| Nat \| \| 96 \| Arabis glabra \| AF144333.1 \| DQ310542.1 \| N \|  \| Nat \| \| 97 \| Arabis hirsuta \| NC_009268.1 \| NC_009268.1 \| N \|  \| Nat \| \| 98 \| Arabis petraea \| AF144336.1 \| JN890772.1 \| N \|  \| Nat \| \| 99 \| Arabis scabra \| \| T.S. \| N \|  \| Nat \| \| 100 \| Arbutus unedo \| T.S. \| T.S. \| N \|  \| Nat \| \| 101 \| Arctium lappa \| JN895220.1 \| JQ933225.1 \| AR \|  \| Inv \| \| 102 \| Arctium minus \| AY013521.1 \| HM849788.1 \| N \|  \| Nat \| \| 103 \| Arctostaphylos uva-ursi \| AF440411.1 \| GU176649.1 \| N \|  \| Nat \| \| 104 \| Arenaria ciliata \| DQ901542.1 \| T.S. \| N \|  \| Nat \| \| 105 \| Arenaria serpyllifolia \| JN895833.1 \| HQ589962.1 \| N \|  \| Nat \| \| 106 \| Armeria arenaria \| \| T.S. \| N \|  \| Nat \| \| 107 \| Armeria maritima \| GQ901433.1 \| HM849792.1 \| N \|  \| Nat \| \| 108 \| Armoracia rusticana \| FN597648.1 \| AF020323.1 \| AR \|  \| Non-inv \| \| 109 \| Arrhenatherum elatius \| AM234543.1 \| AJ784823.1 \| N \|  \| Nat \| \| 110 \| Artemisia absinthium \| JN894750.1 \| JN892095.1 \| AR \|  \| Non-inv \| \| 111 \| Artemisia campestris \| JN894047.1 \| JN890800.1 \| N \|  \| Nat \| \| 112 \| Artemisia vulgaris \| JN894753.1 \| JN891751.1 \| AR \|  \| Inv \| \| 113 \| Arum italicum \| EU886517.1 \| HQ901583.1 \| N \|  \| Nat \| \| 114 \| Arum maculatum \| EU193641.1 \| GU067582.1 \| N \|  \| Nat \| \| 115 \| Asparagus officinalis \| JQ276399.1 \| JQ273904.1 \| N \|  \| Nat \| \| 116 \| Asperula cynanchica \| HE970677.1 \| JN892424.1 \| N \|  \| Nat \| \| 117 \| Asplenium adiantum-nigrum \| \| EF463143.1 \| N \|  \| Nat \| \| 118 \| Asplenium marinum \| \| AF240647.1 \| N \|  \| Nat \| \| 119 \| Asplenium onopteris \| \| AY300131.1 \| N \|  \| Nat \| \| 120 \| Asplenium ruta-muraria \| JF832253.1 \| AF525273.1 \| N \|  \| Nat \| \| 121 \| Asplenium septentrionale \| JF832254.1 \| AF525275.1 \| N \|  \| Nat \| \| 122 \| Asplenium trichomanes \| JF832256.1 \| EF463157.1 \| N \|  \| Nat \| \| 123 \| Asplenium viride \| \| AF240649.1 \| N \|  \| Nat \| \| 124 \| Aster lanceolatus \| EU749434.1 \| EU677042.1 \| AN \|  \| Non-inv \| \| 125 \| Aster linosyris \| JN895153 \| JN892320 \| N \|  \| Nat \| \| 126 \| Aster novae-angliae \| EU749440 \| GU817740.1 \| AN \|  \| Non-inv \| \| 127 \| Aster novi-belgii \| \| T.S. \| AN \|  \| Non-inv \| \| 128 \| Aster tripolium \| JN894915.1 \| JN891980.1 \| N \|  \| Nat \| \| 129 \| Astragalus alpinus \| JN966121.1 \| JN965276.1 \| N \|  \| Nat \| \| 130 \| Astragalus glycyphyllos \| JN894914.1 \| JN891850.1 \| N \|  \| Nat \| \| 131 \| Athyrium distentifolium \| \| EF463304.1 \| N \|  \| Nat \| \| 132 \| Athyrium filix-femina \| JF303941.1 \| JF832056.1 \| N \|  \| Nat \| \| 133 \| Atriplex glabriuscula \| JN894916.1 \| JN965282.1 \| N \|  \| Nat \| \| 134 \| Atriplex laciniata \| JN894220.1 \| JN891030.1 \| N \|  \| Nat \| \| 135 \| Atriplex littoralis \| JN894951.1 \| FR775291.1 \| N \|  \| Nat \| \| 136 \| Atriplex longipes \| \| JN890999.1 \| N \|  \| Nat \| \| 137 \| Atriplex patula \| HM850759.1 \| HM849801.1 \| N \|  \| Nat \| \| 138 \| Atriplex portulacoides \| DQ468648.1 \| JN892551.1 \| N \|  \| Nat \| \| 139 \| Atriplex prostrata \| JN894517.1 \| HM849802.1 \| N \|  \| Nat \| \| 140 \| Atropa belladonna \| NC_004561.1 \| NC_004561.1 \| N \|  \| Nat \| \| 141 \| Avena fatua \| EU833847.1 \| AJ746257.1 \| AR \|  \| Inv \| \| 142 \| Avena sterilis \| GU367305.1 \| HE963347.1 \| AN \|  \| Non-inv \| \| 143 \| Azolla filiculoides \| \| EF520926.1 \| AN \| 1 \| Inv \| \| 144 \| Baldellia ranunculoides \| HQ456458.1 \| HM849805.1 \| N \|  \| Nat \| \| 145 \| Ballota nigra \| JN896121.1 \| HM849806.1 \| AR \|  \| Non-inv \| \| 146 \| Barbarea intermedia \| \| T.S. \| AN \|  \| Non-inv \| \| 147 \| Barbarea verna \| AP009370.1 \| AP009370.1 \| AN \|  \| Non-inv \| \| 148 \| Barbarea vulgaris \| AF144330.1 \| HQ589972.1 \| N \|  \| Nat \| \| 149 \| Bartsia alpina \| AY849600.1 \| AF190903.1 \| N \|  \| Nat \| \| 150 \| Bellis perennis \| JN893935.1 \| AY395530.1 \| N \|  \| Nat \| \| 151 \| Berula erecta \| JN895702.1 \| AM234813.1 \| N \|  \| Nat \| \| 152 \| Beta vulgaris \| AY514832.1 \| DQ067450.1 \| N \|  \| Nat \| \| 153 \| Betula nana \| AY372021.1 \|  \| N \|  \| Nat \| \| 154 \| Betula pendula \| AY372008.1 \| JN892640.1 \| N \|  \| Nat \| \| 155 \| Betula pubescens \| AY372025.1 \| JN893280.1 \| N \|  \| Nat \| \| 156 \| Bidens cernua \| JN893870.1 \| HQ589973.1 \| N \|  \| Nat \| \| 157 \| Bidens tripartita \| JN896081.1 \| JN891149.1 \| N \|  \| Nat \| \| 158 \| Blackstonia perfoliata \| HM850819.1 \| HM849816.1 \| N \|  \| Nat \| \| 159 \| Blechnum spicant \| JF832262.1 \| AB040571.1 \| N \|  \| Nat \| \| 160 \| Blysmus compressus \| \| AJ404700.1 \| N \|  \| Nat \| \| 161 \| Blysmus rufus \| \| JN892567.1 \| N \|  \| Nat \| \| 162 \| Bolboschoenus maritimus \| HM850837.1 \| JF313185.1 \| N \|  \| Nat \| \| 163 \| Botrychium lunaria \| \| AB574664.1 \| N \|  \| Nat \| \| 164 \| Brachypodium pinnatum \| DQ786891.1 \| AM849347.1 \| N \|  \| Nat \| \| 165 \| Brachypodium sylvaticum \| AF164400.1 \| AJ746258.1 \| N \|  \| Nat \| \| 166 \| Brassica napus \| NC_016734.1 \| NC_016734.1 \| AN \|  \| Non-inv \| \| 167 \| Brassica rapa \| AY541619.1 \| AY167977.1 \| AR \|  \| Non-inv \| \| 168 \| Briza maxima \| FN908048.1 \| FN870384.1 \| AN \|  \| Non-inv \| \| 169 \| Briza media \| AM234610.1 \| AJ746285.1 \| N \|  \| Nat \| \| 170 \| Briza minor \| DQ786892.1 \| HQ600461.1 \| AR \|  \| Non-inv \| \| 171 \| Bromopsis benekenii \| JN894144.1 \| JN890931.1 \| N \|  \| Nat \| \| 172 \| Bromopsis erecta \| AM234570.1 \| AJ746286.1 \| N \|  \| Nat \| \| 173 \| Bromopsis inermis \| AF164398.1 \| HQ652722.1 \| AN \|  \| Non-inv \| \| 174 \| Bromopsis ramosa \| HE586076.1 \| HE575814.1 \| N \|  \| Nat \| \| 175 \| Bromus commutatus \| JN895848.1 \| AJ746287.1 \| N \|  \| Nat \| \| 176 \| Bromus hordeaceus \| HE573929.1 \| AY395531.1 \| N \|  \| Nat \| \| 177 \| Bromus racemosus \| JN895847.1 \| JN892639.1 \| N \|  \| Nat \| \| 178 \| Bromus secalinus \| AM234571.1 \| JN892641.1 \| AR \|  \| Non-inv \| \| 179 \| Bryonia dioica \| DQ536641.1 \| DQ535786.1 \| N \|  \| Nat \| \| 180 \| Buddleja davidii \| HQ384530.1 \| AJ001757.1 \| AN \|  \| Inv \| \| 181 \| Bunium bulbocastanum \| \| T.S. \| N \|  \| Nat \| \| 182 \| Bupleurum falcatum \| U58552.1 \| U50224.1 \| AN \|  \| Non-inv \| \| 183 \| Bupleurum tenuissimum \| JN895623.1 \| JN892970.1 \| N \|  \| Nat \| \| 184 \| Butomus umbellatus \| DQ401367.1 \| AY149345.1 \| N \|  \| Nat \| \| 185 \| Buxus sempervirens \| AF543728.1 \| DQ182333.1 \| N \|  \| Nat \| \| 186 \| Cakile maritima \| JN584959.1 \| AY167981.1 \| N \|  \| Nat \| \| 187 \| Calamagrostis canescens \| JN894176.1 \| JN892634.1 \| N \|  \| Nat \| \| 188 \| Calamagrostis epigejos \| JN894015.1 \| AJ784820.1 \| N \|  \| Nat \| \| 189 \| Calendula officinalis \| AF151446.1 \| HM849835.1 \| AN \|  \| Non-inv \| \| 190 \| Callitriche brutia \| \| AF248009.1 \| N \|  \| Nat \| \| 191 \| Callitriche hamulata \| JN896066.1 \| AF248013.1 \| N \|  \| Nat \| \| 192 \| Callitriche hermaphroditica \| JN894013.1 \| L36441.1 \| N \|  \| Nat \| \| 193 \| Callitriche obtusangula \| JN896068.1 \| JN893579.1 \| N \|  \| Nat \| \| 194 \| Callitriche platycarpa \| JN896071.1 \| AF248022.1 \| N \|  \| Nat \| \| 195 \| Callitriche stagnalis \| JN893980.1 \| AF248023.1 \| N \|  \| Nat \| \| 196 \| Callitriche truncata \| JN895908.1 \| AF248025.1 \| N \|  \| Nat \| \| 197 \| Calluna vulgaris \| JN894846.1 \| HM849840.1 \| N \|  \| Nat \| \| 198 \| Caltha palustris \| AB069845.1 \| AY395532.1 \| N \|  \| Nat \| \| 199 \| Calystegia pulchra \| \| T.S. \| AN \|  \| Non-inv \| \| 200 \| Calystegia sepium \| FJ395438.1 \| AY100992.1 \| N \|  \| Nat \| \| 201 \| Calystegia silvatica \| T.S. \| T.S. \| AN \|  \| Inv \| \| 202 \| Calystegia soldanella \| JN894768.1 \| JN891775.1 \| N \|  \| Nat \| \| 203 \| Camelina sativa \| GQ424578.1 \| \| AR \|  \| Non-inv \| \| 204 \| Campanula glomerata \| JN894555.1 \| JN891483.1 \| N \|  \| Nat \| \| 205 \| Campanula latifolia \| EU713271.1 \| JN891483.1 \| N \|  \| Nat \| \| 206 \| Campanula patula \| JN894553.1 \| JN891766.1 \| N \|  \| Nat \| \| 207 \| Campanula persicifolia \| EU713324.1 \| FJ587264.1 \| AN \|  \| Non-inv \| \| 208 \| Campanula portenschlagiana \| \| T.S. \| AN \|  \| Non-inv \| \| 209 \| Campanula poscharskyana \| \| FJ587266.1 \| AN \|  \| Inv \| \| 210 \| Campanula rapunculoides \| EU713285.1 \| FJ587271.1 \| AN \|  \| Non-inv \| \| 211 \| Campanula rapunculus \| JN893981.1 \| FJ587272.1 \| AR \|  \| Non-inv \| \| 212 \| Campanula rotundifolia \| JN571958.1 \| FJ587273.1 \| N \|  \| Nat \| \| 213 \| Campanula trachelium \| JN896225.1 \| FJ587285.1 \| N \|  \| Nat \| \| 214 \| Capsella bursa-pastoris \| AP009371.1 \| AP009371.1 \| AR \|  \| Non-inv \| \| 215 \| Cardamine amara \| AF144337.1 \| JN891288.1 \| N \|  \| Nat \| \| 216 \| Cardamine bulbifera \| \| T.S. \| N \|  \| Nat \| \| 217 \| Cardamine flexuosa \| JN894558.1 \| D88905.1 \| N \|  \| Nat \| \| 218 \| Cardamine hirsuta \| HM850748.1 \| HM849847.1 \| N \|  \| Nat \| \| 219 \| Cardamine impatiens \| JF953419.1 \| JN847832.1 \| N \|  \| Nat \| \| 220 \| Cardamine pratensis \| JN894921.1 \| HM849848.1 \| N \|  \| Nat \| \| 221 \| Carduus crispus \| JN894376.1 \| JN893592.1 \| N \|  \| Nat \| \| 222 \| Carduus nutans \| JN894377.1 \| JN893595.1 \| N \|  \| Nat \| \| 223 \| Carduus tenuiflorus \| JN894378.1 \| HM849849.1 \| N \|  \| Nat \| \| 224 \| Carex acuta \| JN895251.1 \| JN892450.1 \| N \|  \| Nat \| \| 225 \| Carex acutiformis \| JN895249.1 \| JN892448.1 \| N \|  \| Nat \| \| 226 \| Carex aquatilis \| FN668461.1 \| JN892453.1 \| N \|  \| Nat \| \| 227 \| Carex arenaria \| JN895639.1 \| JN892994.1 \| N \|  \| Nat \| \| 228 \| Carex atrata \|  \| JX644605.1 \| N \|  \| Nat \| \| 229 \| Carex atrofusca \| FJ548074.1 \| FJ548250.1 \| N \|  \| Nat \| \| 230 \| Carex bigelowii \| JN895168.1 \| JN892344.1 \| N \|  \| Nat \| \| 231 \| Carex binervis \| JN896034.1 \| JN893529.1 \| N \|  \| Nat \| \| 232 \| Carex capillaris \| FJ548087.1 \| FJ548254.1 \| N \|  \| Nat \| \| 233 \| Carex caryophyllea \| JN895022.1 \| JN893402.1 \| N \|  \| Nat \| \| 234 \| Carex chordorrhiza \| JN966176.1 \| FJ548256.1 \| N \|  \| Nat \| \| 235 \| Carex curta \| JN895021.1 \| GQ469845.1 \| N \|  \| Nat \| \| 236 \| Carex depauperata \| JN895024.1 \| JN893398.1 \| N \|  \| Nat \| \| 237 \| Carex diandra \| JN895023.1 \| JN892140.1 \| N \|  \| Nat \| \| 238 \| Carex digitata \| \| JN892144.1 \| N \|  \| Nat \| \| 239 \| Carex dioica \| JN895942.1 \| JN892784.1 \| N \|  \| Nat \| \| 240 \| Carex distans \| JN895486.1 \| GQ469848.1 \| N \|  \| Nat \| \| 241 \| Carex disticha \| JN895943.1 \| JN892788.1 \| N \|  \| Nat \| \| 242 \| Carex divisa \|  \| JN892780.1 \| N \|  \| Nat \| \| 243 \| Carex divulsa \| JN895562.1 \| HM849851.1 \| N \|  \| Nat \| \| 244 \| Carex echinata \| JN895483.1 \| HM849852.1 \| N \|  \| Nat \| \| 245 \| Carex elata \| JN895554.1 \| JN892568.1 \| N \|  \| Nat \| \| 246 \| Carex elongata \| JN895742.1 \| JN893126.1 \| N \|  \| Nat \| \| 247 \| Carex extensa \| JN895345.1 \| GQ469846.1 \| N \|  \| Nat \| \| 248 \| Carex flacca \| JN895262.1 \| JN891463.1 \| N \|  \| Nat \| \| 249 \| Carex hirta \| JN896155.1 \| JN892186.1 \| N \|  \| Nat \| \| 250 \| Carex hostiana \| JN895279.1 \| GQ469841.1 \| N \|  \| Nat \| \| 251 \| Carex humilis \| FR865043.1 \| FR865118.1 \| N \|  \| Nat \| \| 252 \| Carex lachenalii \| FJ548105.1 \| FJ548262.1 \| N \|  \| Nat \| \| 253 \| Carex laevigata \| T.S. \| JN891460.1 \| N \|  \| Nat \| \| 254 \| Carex lasiocarpa \| JN895750.1 \| JN891455.1 \| N \|  \| Nat \| \| 255 \| Carex limosa \| JN896254.1 \| JX644630.1 \| N \|  \| Nat \| \| 256 \| Carex magellanica \| JN896255.1 \| GQ469849.1 \| N \|  \| Nat \| \| 257 \| Carex maritima \| JN966193.1 \| GQ469840.1 \| N \|  \| Nat \| \| 258 \| Carex microglochin \| \| GQ469844.1 \| N \|  \| Nat \| \| 259 \| Carex montana \| JN895152.1 \| JN891206.1 \| N \|  \| Nat \| \| 260 \| Carex muricata \| JN896252.1 \| JN891204.1 \| N \|  \| Nat \| \| 261 \| Carex nigra \| JN895336.1 \| HM849856.1 \| N \|  \| Nat \| \| 262 \| Carex norvegica \| FJ548127.1 \|  \| N \|  \| Nat \| \| 263 \| Carex otrubae \| JN896253.1 \| HM849857.1 \| N \|  \| Nat \| \| 264 \| Carex ovalis \| HM850845.1 \| HM849858.1 \| N \|  \| Nat \| \| 265 \| Carex pallescens \| JN896250.1 \| JN891906.1 \| N \|  \| Nat \| \| 266 \| Carex panicea \| JN896251.1 \| HM849860.1 \| N \|  \| Nat \| \| 267 \| Carex paniculata \| JN894850.1 \| JN892598.1 \| N \|  \| Nat \| \| 268 \| Carex pauciflora \| JN896248.1 \| GQ469850.1 \| N \|  \| Nat \| \| 269 \| Carex pendula \| JN896249.1 \| HM849861.1 \| N \|  \| Nat \| \| 270 \| Carex pilulifera \| JN893867.1 \| HM849863.1 \| N \|  \| Nat \| \| 271 \| Carex pseudocyperus \| JN894711.1 \| JN891696.1 \| N \|  \| Nat \| \| 272 \| Carex pulicaris \| JN894931.1 \| GQ469843.1 \| N \|  \| Nat \| \| 273 \| Carex punctata \| JN896087.1 \| HM849864.1 \| N \|  \| Nat \| \| 274 \| Carex rariflora \| JN966199.1 \| FJ548276.1 \| N \|  \| Nat \| \| 275 \| Carex recta \|  \| FJ904602.1 \| N \|  \| Nat \| \| 276 \| Carex remota \| JN894576.1 \| JN893762.1 \| N \|  \| Nat \| \| 277 \| Carex riparia \| JN896086.1 \| JN893598.1 \| N \|  \| Nat \| \| 278 \| Carex rostrata \| JN896202.1 \| GQ469851.1 \| N \|  \| Nat \| \| 279 \| Carex rupestris \| FJ548133.1 \| FJ548278.1 \| N \|  \| Nat \| \| 280 \| Carex saxatilis \| JN966206.1 \| FJ548280.1 \| N \|  \| Nat \| \| 281 \| Carex spicata \| JN894304.1 \| JN893600.1 \| N \|  \| Nat \| \| 282 \| Carex strigosa \| JN896041.1 \| JN893544.1 \| N \|  \| Nat \| \| 283 \| Carex sylvatica \| JN896090.1 \| JN893602.1 \| N \|  \| Nat \| \| 284 \| Carex vaginata \| JN966214.1 \| JN965420.1 \| N \|  \| Nat \| \| 285 \| Carex vesicaria \| JN314531.1 \| JN892608.1 \| N \|  \| Nat \| \| 286 \| Carex viridula \| JN896091.1 \| HM849865.1 \| N \|  \| Nat \| \| 287 \| Carex vulpina \| \| T.S. \| N \|  \| Nat \| \| 288 \| Carlina vulgaris \| JN895932.1 \| JN892332.1 \| N \|  \| Nat \| \| 289 \| Carpinus betulus \| AJ417512.1 \| AY263928.1 \| N \|  \| Nat \| \| 290 \| Carpobrotus edulis \| JQ024942.1 \| AM234787.1 \| AN \| 1 \| Inv \| \| 291 \| Carum carvi \| U58553.1 \|  \| AR \|  \| Non-inv \| \| 292 \| Carum verticillatum \| JN894449.1 \| JN893496.1 \| N \|  \| Nat \| \| 293 \| Castanea sativa \| JN895513.1 \| AF500363.1 \| AR \|  \| Inv \| \| 294 \| Catabrosa aquatica \| DQ786898.1 \| JN892646.1 \| N \|  \| Nat \| \| 295 \| Catapodium marinum \| JN895498.1 \| JN892800.1 \| N \|  \| Nat \| \| 296 \| Catapodium rigidum \| JN895497.1 \| JN892364.1 \| N \|  \| Nat \| \| 297 \| Centaurea cyanus \| JN894130.1 \| AB530955.1 \| AR \|  \| Non-inv \| \| 298 \| Centaurea nigra \| JN895499.1 \| JN891108.1 \| N \|  \| Nat \| \| 299 \| Centaurea scabiosa \| JN894134.1 \| JN892734.1 \| N \|  \| Nat \| \| 300 \| Centaurium erythraea \| JN894719.1 \| HM849874.1 \| N \|  \| Nat \| \| 301 \| Centaurium littorale \| JN895504.1 \| JN892806.1 \| N \|  \| Nat \| \| 302 \| Centaurium pulchellum \| JN895503.1 \| HM849876.1 \| N \|  \| Nat \| \| 303 \| Centaurium scilloides \| JN894297.1 \| HM849877.1 \| N \|  \| Nat \| \| 304 \| Centaurium tenuiflorum \| HM850823.1 \| HM849878.1 \| N \|  \| Nat \| \| 305 \| Centranthus ruber \| AF446926.1 \| HM849879.1 \| AN \|  \| Inv \| \| 306 \| Cephalanthera damasonium \| \| AF074123.1 \| N \|  \| Nat \| \| 307 \| Cephalanthera longifolia \| \| JX051381.1 \| N \|  \| Nat \| \| 308 \| Cephalanthera rubra \| \| JX088502.1 \| N \|  \| Nat \| \| 309 \| Cerastium alpinum \| JN894309.1 \| JN890578.1 \| N \|  \| Nat \| \| 310 \| Cerastium arcticum \| JN894854.1 \| JN890804.1 \| N \|  \| Nat \| \| 311 \| Cerastium arvense \| AY936295.1 \| JN892004.1 \| N \|  \| Nat \| \| 312 \| Cerastium brachypetalum \| \| T.S. \| AN \|  \| Non-inv \| \| 313 \| Cerastium diffusum \| JN895181.1 \| JN892002.1 \| N \|  \| Nat \| \| 314 \| Cerastium fontanum \| FJ404829.1 \| HM849881.1 \| N \|  \| Nat \| \| 315 \| Cerastium glomeratum \| JN895359.1 \| HM849882.1 \| N \|  \| Nat \| \| 316 \| Cerastium nigrescens \| \| T.S. \| NE \|  \| Nat \| \| 317 \| Cerastium pumilum \| JN894932.1 \| JN892000.1 \| N \|  \| Nat \| \| 318 \| Cerastium semidecandrum \| JN895252.1 \| JN892588.1 \| N \|  \| Nat \| \| 319 \| Cerastium tomentosum \| \| T.S. \| AN \|  \| Non-inv \| \| 320 \| Ceratocapnos claviculata \| JN894940.1 \| JN892014.1 \| N \|  \| Nat \| \| 321 \| Ceratochloa carinata \| \| HQ600457.1 \| AN \|  \| Non-inv \| \| 322 \| Ceratochloa cathartica \| FN908049.1 \| FN870385.1 \| AN \|  \| Non-inv \| \| 323 \| Ceratophyllum demersum \| NC_009962.1 \| NC_009962.1 \| N \|  \| Nat \| \| 324 \| Ceratophyllum submersum \| DQ401361.1 \| AF197599.1 \| N \|  \| Nat \| \| 325 \| Ceterach officinarum \| \| AF240643.1 \| N \|  \| Nat \| \| 326 \| Chaenorhinum minus \| JN895897.1 \| JN892462.1 \| AR \|  \| Non-inv \| \| 327 \| Chaerophyllum temulum \| FJ395423.1 \| JN893229.1 \| N \|  \| Nat \| \| 328 \| Chamaecyparis lawsoniana \| HQ245883.1 \| AY380880.1 \| AN \|  \| Inv \| \| 329 \| Chamaemelum nobile \| JN895754.1 \| HM849885.1 \| N \|  \| Nat \| \| 330 \| Chamerion angustifolium \| JN894188.1 \| L10217.1 \| N \|  \| Nat \| \| 331 \| Chelidonium majus \| HQ593230.1 \| HM849887.1 \| AR \|  \| Non-inv \| \| 332 \| Chenopodium album \| JN894388.1 \| HM849888.1 \| N \|  \| Nat \| \| 333 \| Chenopodium bonus-henricus \| HE855613.1 \| AY270079.1 \| AR \|  \| Non-inv \| \| 334 \| Chenopodium chenopodioides \| HE855622.1 \|  \| N \|  \| Nat \| \| 335 \| Chenopodium ficifolium \| HE855666.1 \| JN891252.1 \| AR \|  \| Non-inv \| \| 336 \| Chenopodium glaucum \| HE855627.1 \| JF941276.1 \| AR \|  \| Non-inv \| \| 337 \| Chenopodium hybridum \| HE855635.1 \|  \| AR \|  \| Non-inv \| \| 338 \| Chenopodium murale \| HE855633.1 \| HM849890.1 \| AR \|  \| Non-inv \| \| 339 \| Chenopodium polyspermum \| HE855631.1 \| JN892910.1 \| AR \|  \| Non-inv \| \| 340 \| Chenopodium rubrum \| HE855625.1 \| JN892679.1 \| N \|  \| Nat \| \| 341 \| Chenopodium urbicum \| HE855630.1 \| HM587596.1 \| AR \|  \| Non-inv \| \| 342 \| Chenopodium vulvaria \| HE855640.1 \| JN892912.1 \| AR \|  \| Non-inv \| \| 343 \| Chrysanthemum segetum \| JN896039.1 \| HM849893.1 \| AR \|  \| Non-inv \| \| 344 \| Chrysosplenium alternifolium \| AM396496.2 \| JN892258.1 \| N \|  \| Nat \| \| 345 \| Chrysosplenium oppositifolium \| JN894080.1 \| JN891003.1 \| N \|  \| Nat \| \| 346 \| Cicendia filiformis \| HM850824.1 \| HM849894.1 \| N \|  \| Nat \| \| 347 \| Cicerbita alpina \| AJ633233.1 \|  \| N \|  \| Nat \| \| 348 \| Cicerbita macrophylla \| \| T.S. \| AN \|  \| Non-inv \| \| 349 \| Cichorium intybus \| JN895731.1 \| HM849895.1 \| AR \|  \| Non-inv \| \| 350 \| Cicuta virosa \| JN966246.1 \| JN893232.1 \| N \|  \| Nat \| \| 351 \| Circaea alpina \| \| L10216.1 \| N \|  \| Nat \| \| 352 \| Circaea lutetiana \| T.S. \| HQ590036.1 \| N \|  \| Nat \| \| 353 \| Cirsium acaule \| JN895488.1 \| JN892902.1 \| N \|  \| Nat \| \| 354 \| Cirsium arvense \| HQ593238.1 \| HM849897.1 \| N \|  \| Nat \| \| 355 \| Cirsium dissectum \| JN895541.1 \| JN892904.1 \| N \|  \| Nat \| \| 356 \| Cirsium eriophorum \| JN895575.1 \| JN892858.1 \| N \|  \| Nat \| \| 357 \| Cirsium heterophyllum \| JN894434.1 \| JN892860.1 \| N \|  \| Nat \| \| 358 \| Cirsium palustre \| JN895512.1 \| HM849898.1 \| N \|  \| Nat \| \| 359 \| Cirsium tuberosum \| JN894436.1 \| JN892654.1 \| N \|  \| Nat \| \| 360 \| Cirsium vulgare \| JN895404.1 \| HM849899.1 \| N \|  \| Nat \| \| 361 \| Cladium mariscus \| JN895569.1 \| HM849902.1 \| N \|  \| Nat \| \| 362 \| Claytonia perfoliata \| AY764091.1 \| AF132093.1 \| AN \|  \| Non-inv \| \| 363 \| Claytonia sibirica \| JX456286.1 \| T.S. \| AN \|  \| Inv \| \| 364 \| Clematis vitalba \| AB110525.1 \| FR865151.1 \| N \|  \| Nat \| \| 365 \| Clinopodium acinos \| AY840144.1 \| JN890568.1 \| N \|  \| Nat \| \| 366 \| Clinopodium calamintha \| AY840151.1 \|  \| N \|  \| Nat \| \| 367 \| Clinopodium menthifolium \| HM850807.1 \| \| N \|  \| Nat \| \| 368 \| Clinopodium vulgare \| AY840153.1 \| HM849904.1 \| N \|  \| Nat \| \| 369 \| Cochlearia anglica \| JN895334.1 \| JN892554.1 \| N \|  \| Nat \| \| 370 \| Cochlearia danica \| AF174531.1 \| JN892594.1 \| N \|  \| Nat \| \| 371 \| Cochlearia officinalis \| JN895761.1 \| JN893155.1 \| N \|  \| Nat \| \| 372 \| Cochlearia pyrenaica \| AF144357.1 \| JN891278.1 \| N \|  \| Nat \| \| 373 \| Coeloglossum viride \| JN894050.1 \| JN890805.1 \| N \|  \| Nat \| \| 374 \| Coincya monensis \| JN584961.1 \| JN891266.1 \| N \|  \| Nat \| \| 375 \| Colchicum autumnale \| JN895471.1 \| HF572820.1 \| N \|  \| Nat \| \| 376 \| Colutea arborescens \| \| T.S. \| AN \|  \| Non-inv \| \| 377 \| Conium maculatum \| JN895815.1 \| HM849908.1 \| AR \|  \| Inv \| \| 378 \| Conopodium majus \| JN894264.1 \| JN893224.1 \| N \|  \| Nat \| \| 379 \| Consolida ajacis \| FJ626496.1 \| FJ626582.1 \| AN \|  \| Non-inv \| \| 380 \| Convallaria majalis \| JN896035.1 \| HM640443.1 \| N \|  \| Nat \| \| 381 \| Convolvulus arvensis \| JN894765.1 \| AY100993.1 \| N \|  \| Nat \| \| 382 \| Conyza canadensis \| HM850627.1 \| HM849912.1 \| AN \|  \| Non-inv \| \| 383 \| Conyza sumatrensis \| \| HM849913.1 \| AN \|  \| Inv \| \| 384 \| Corallorhiza trifida \| JN966252.1 \| EU391358.1 \| N \|  \| Nat \| \| 385 \| Coriandrum sativum \| U58557.1 \| HM849916.1 \| AN \|  \| Non-inv \| \| 386 \| Cornus sanguinea \| DQ340472.1 \| FJ395589.1 \| N \|  \| Nat \| \| 387 \| Cornus sericea \| EU749305.1 \| AY725857.1 \| AN \|  \| Non-inv \| \| 388 \| Cornus suecica \| DQ341334.1 \| AF421085.1 \| N \|  \| Nat \| \| 389 \| Coronopus didymus \| JF926656.1 \| JN847819.1 \| AN \|  \| Non-inv \| \| 390 \| Coronopus squamatus \| HM850736.1 \| HM850119.1 \| AR \|  \| Non-inv \| \| 391 \| Corrigiola litoralis \| FN825767 \| FN868311.1 \| N \|  \| Nat \| \| 392 \| Corylus avellana \| AY373445.1 \| HM849918.1 \| N \|  \| Nat \| \| 393 \| Corynephorus canescens \| T.S. \| FN870793.1 \| N \|  \| Nat \| \| 394 \| Cotoneaster bullatus \| \| JQ391110.1 \| AN \|  \| Non-inv \| \| 395 \| Cotoneaster cambricus \| JN894462.1 \| JN891350.1 \| AN \| 1 \| Non-inv \| \| 396 \| Cotoneaster horizontalis \| JQ390947.1 \| JQ391284.1 \| AN \| 1 \| Non-inv \| \| 397 \| Cotoneaster microphyllus agg. \| JQ390958.1 \| JQ391296.1 \| AN \| 1 \| Inv \| \| 398 \| Cotoneaster simonsii \| \| T.S. \| AN \| 1 \| Inv \| \| 399 \| Crambe maritima \| JN584965.1 \| JN893186.1 \| N \|  \| Nat \| \| 400 \| Crassula helmsii \| \| T.S. \| AN \| 1 \| Inv \| \| 401 \| Crassula tillaea \| HM850702.1 \| HM849921.1 \| N \|  \| Nat \| \| 402 \| Crataegus laevigata \| JN893930.1 \| JN890654.1 \| N \|  \| Nat \| \| 403 \| Crataegus monogyna \| AF288099.1 \| FN689370.1 \| N \|  \| Nat \| \| 404 \| Crepis biennis \| JN894644.1 \| JN892650.1 \| N \|  \| Nat \| \| 405 \| Crepis capillaris \| JN894641.1 \| JN893254.1 \| N \|  \| Nat \| \| 406 \| Crepis mollis \| JN894642.1 \| JN892236.1 \| N \|  \| Nat \| \| 407 \| Crepis paludosa \| JN895244.1 \| JN892441.1 \| N \|  \| Nat \| \| 408 \| Crepis praemorsa \| EU363578.1 \|  \| N \|  \| Nat \| \| 409 \| Crepis vesicaria \| \| T.S. \| AN \|  \| Non-inv \| \| 410 \| Crithmum maritimum \| U58558.1 \| HM849924.1 \| N \|  \| Nat \| \| 411 \| Cruciata laevipes \| JN894637.1 \| JN893414.1 \| N \|  \| Nat \| \| 412 \| Cryptogramma crispa \| JF832265.1 \| EF452148.1 \| N \|  \| Nat \| \| 413 \| Cuscuta epithymum \| \| AY558866.1 \| N \|  \| Nat \| \| 414 \| Cuscuta europaea \| EU330282.1 \| EU330277.1 \| N \|  \| Nat \| \| 415 \| Cymbalaria muralis \| HM850958.1 \| HM849929.1 \| AN \|  \| Inv \| \| 416 \| Cynoglossum officinale \| JN895550.1 \| EU599840.1 \| N \|  \| Nat \| \| 417 \| Cynosurus cristatus \| DQ786901.1 \| HM849932.1 \| N \|  \| Nat \| \| 418 \| Cyperus fuscus \| \| AM999806.1 \| N \|  \| Nat \| \| 419 \| Cyperus longus \| JN894347.1 \| HM849940.1 \| N \|  \| Nat \| \| 420 \| Cypripedium calceolus \| AY557208.1 \| EF370100.1 \| N \|  \| Nat \| \| 421 \| Cystopteris fragilis \| JF832266.1 \| JF832062.1 \| N \|  \| Nat \| \| 422 \| Cystopteris montana \| JF832267.1 \| JF832063.1 \| N \|  \| Nat \| \| 423 \| Cytisus scoparius \| JN894661.1 \| HM849943.1 \| N \|  \| Nat \| \| 424 \| Daboecia cantabrica \| U61349.1 \| L12611.2 \| N \|  \| Nat \| \| 425 \| Dactylis glomerata \| AM234595.1 \| HM849945.1 \| N \|  \| Nat \| \| 426 \| Dactylorhiza fuchsii \| JN895597.1 \| JQ933294.1 \| N \|  \| Nat \| \| 427 \| Dactylorhiza incarnata \| JN893964.1 \| JN890700.1 \| N \|  \| Nat \| \| 428 \| Dactylorhiza lapponica \| \| T.S. \| N \|  \| Nat \| \| 429 \| Dactylorhiza maculata \| EF612529.1 \| JN893308.1 \| N \|  \| Nat \| \| 430 \| Dactylorhiza majalis \| AM883562.1 \| JN890784.1 \| N \|  \| Nat \| \| 431 \| Dactylorhiza praetermissa \| JN894795.1 \| JN892130.1 \| N \|  \| Nat \| \| 432 \| Dactylorhiza purpurella \| JN896212.1 \| JN893778.1 \| N \|  \| Nat \| \| 433 \| Dactylorhiza traunsteineri \| \| JN891811.1 \| N \|  \| Nat \| \| 434 \| Damasonium alisma \| HQ456462.1 \| U80678.1 \| N \|  \| Nat \| \| 435 \| Danthonia decumbens \| FN908050.1 \| EU400662.1 \| N \|  \| Nat \| \| 436 \| Daphne laureola \| JN894952.1 \| HM849946.1 \| N \|  \| Nat \| \| 437 \| Datura stramonium \| NC_018117.1 \| NC_018117.1 \| AN \|  \| Non-inv \| \| 438 \| Daucus carota \| NC_008325.1 \| NC_008325.1 \| N \|  \| Nat \| \| 439 \| Deschampsia cespitosa \| AM234546.1 \| EF125152.1 \| N \|  \| Nat \| \| 440 \| Deschampsia flexuosa \| DQ786887.1 \| JN892160.1 \| N \|  \| Nat \| \| 441 \| Deschampsia setacea \| JN895038.1 \| JN892158.1 \| N \|  \| Nat \| \| 442 \| Descurainia sophia \| JF926654.1 \| JN847829.1 \| AR \|  \| Non-inv \| \| 443 \| Dianthus armeria \| FJ404832.1 \| JN892689.1 \| N \|  \| Nat \| \| 444 \| Dianthus deltoides \| JN895428.1 \| JN892692.1 \| N \|  \| Nat \| \| 445 \| Dianthus gratianopolitanus \| \| T.S. \| N \|  \| Nat \| \| 446 \| Diapensia lapponica \| AJ429283.1 \| L12612.2 \| N \|  \| Nat \| \| 447 \| Digitalis purpurea \| JN894393.1 \| HM849951.1 \| N \|  \| Nat \| \| 448 \| Digitaria sanguinalis \| AF164421.1 \| HM849953.1 \| AN \|  \| Non-inv \| \| 449 \| Diphasiastrum alpinum \| \| AJ133250.1 \| N \|  \| Nat \| \| 450 \| Diphasiastrum complanatum \| \| AB574627.1 \| N \|  \| Nat \| \| 451 \| Diplotaxis muralis \| \| T.S. \| AN \|  \| Non-inv \| \| 452 \| Diplotaxis tenuifolia \| JN894531.1 \| JN892620.1 \| AR \|  \| Non-inv \| \| 453 \| Dipsacus pilosus \| JN894949.1 \| JN892434.1 \| N \|  \| Nat \| \| 454 \| Disphyma crassifolium \| HM850879.1 \| AM234789 \| AN \| 1 \| Non-inv \| \| 455 \| Doronicum pardalianches \| EF537918.2 \| GU817757.1 \| AN \|  \| Non-inv \| \| 456 \| Draba aizoides \| JN894950.1 \| JN892732.1 \| N \|  \| Nat \| \| 457 \| Draba incana \| JN895100.1 \| JN892630.1 \| N \|  \| Nat \| \| 458 \| Draba muralis \| \| T.S. \| N \|  \| Nat \| \| 459 \| Drosera anglica \| JN894432.1 \| AB298091.1 \| N \|  \| Nat \| \| 460 \| Drosera intermedia \| \| JN891175.1 \| N \|  \| Nat \| \| 461 \| Drosera rotundifolia \| JN895618.1 \| AB298089.1 \| N \|  \| Nat \| \| 462 \| Dryas octopetala \| JF317424.1 \| JF317483.1 \| N \|  \| Nat \| \| 463 \| Dryopteris aemula \| \| AY268881.1 \| N \|  \| Nat \| \| 464 \| Dryopteris carthusiana \| \| JQ935272.1 \| N \|  \| Nat \| \| 465 \| Dryopteris cristata \| \| U05923.1 \| N \|  \| Nat \| \| 466 \| Dryopteris dilatata \| \| JQ935269.1 \| N \|  \| Nat \| \| 467 \| Dryopteris expansa \| \| EF463179.1 \| N \|  \| Nat \| \| 468 \| Dryopteris filix-mas \| \| EF463180.1 \| N \|  \| Nat \| \| 469 \| Dryopteris oreades \| \| AY268850.1 \| N \|  \| Nat \| \| 470 \| Dryopteris remota \| \| AY268858.1 \| N \|  \| Nat \| \| 471 \| Dryopteris submontana \| \| AF240653.1 \| N \|  \| Nat \| \| 472 \| Echinochloa crus-galli \| HE574054.1 \| HM849963.1 \| AN \|  \| Non-inv \| \| 473 \| Echium plantagineum \| EU599697.1 \| EU599872.1 \| AR \|  \| Non-inv \| \| 474 \| Echium vulgare \| FJ827257.1 \| JN892864.1 \| N \|  \| Nat \| \| 475 \| Elatine hexandra \| HM850928.1 \| HM849969.1 \| N \|  \| Nat \| \| 476 \| Elatine hydropiper \| \| AJ402948.1 \| N \|  \| Nat \| \| 477 \| Eleocharis acicularis \| JN893901.1 \| GU344675.1 \| N \|  \| Nat \| \| 478 \| Eleocharis austriaca \| \| JX644676 \| N \|  \| Nat \| \| 479 \| Eleocharis multicaulis \| JN895515.1 \| HM849970.1 \| N \|  \| Nat \| \| 480 \| Eleocharis palustris \| HM850943.1 \| AM999826.1 \| N \|  \| Nat \| \| 481 \| Eleocharis parvula \| \| JN891238.1 \| N \|  \| Nat \| \| 482 \| Eleocharis quinqueflora \| JN893900.1 \| JN892182.1 \| N \|  \| Nat \| \| 483 \| Eleocharis uniglumis \| JN896143.1 \| JN892550.1 \| N \|  \| Nat \| \| 484 \| Eleogiton fluitans \| JN893904.1 \| HM850077.1 \| N \|  \| Nat \| \| 485 \| Elodea canadensis \| NC_018541.1 \| NC_018541.1 \| AN \| 1 \| Inv \| \| 486 \| Elodea nuttallii \| AB002568.1 \| AB004888.1 \| AN \| 1 \| Inv \| \| 487 \| Elymus caninus \| JN893902.1 \| JN892154.1 \| N \|  \| Nat \| \| 488 \| Elytrigia atherica \| JN893903.1 \| JN891933.1 \| N \|  \| Nat \| \| 489 \| Elytrigia juncea \| JN893896.1 \| JN891931.1 \| N \|  \| Nat \| \| 490 \| Elytrigia repens \| JN894307.1 \| HQ600453.1 \| N \|  \| Nat \| \| 491 \| Empetrum nigrum \| AF519558.1 \| AF421091.1 \| N \|  \| Nat \| \| 492 \| Epilobium alsinifolium \| \| T.S. \| N \|  \| Nat \| \| 493 \| Epilobium anagallidifolium \| \| T.S. \| N \|  \| Nat \| \| 494 \| Epilobium brunnescens \| \| T.S. \| AN \|  \| Inv \| \| 495 \| Epilobium ciliatum \| T.S. \| T.S. \| AN \|  \| Inv \| \| 496 \| Epilobium hirsutum \| JN894073.1 \| HQ590078.1 \| N \|  \| Nat \| \| 497 \| Epilobium lanceolatum \| JN895959.1 \| JN892697.1 \| N \|  \| Nat \| \| 498 \| Epilobium montanum \| JN895009.1 \| JN892702.1 \| N \|  \| Nat \| \| 499 \| Epilobium obscurum \| HM851000.1 \| HM849975.1 \| N \|  \| Nat \| \| 500 \| Epilobium palustre \| JN966285.1 \| JF941484.1 \| N \|  \| Nat \| \| 501 \| Epilobium parviflorum \| JN895757.1 \| HM849976.1 \| N \|  \| Nat \| \| 502 \| Epilobium roseum \| JN895432.1 \| JN892698.1 \| N \|  \| Nat \| \| 503 \| Epilobium tetragonum \| JN894114.1 \| HM849977.1 \| N \|  \| Nat \| \| 504 \| Epipactis atrorubens \| JN894244.1 \| JX088503 \| N \|  \| Nat \| \| 505 \| Epipactis helleborine \| EU490692.1 \| JX094820.1 \| N \|  \| Nat \| \| 506 \| Epipactis leptochila \| JN894967.1 \| FJ454879.1 \| N \|  \| Nat \| \| 507 \| Epipactis palustris \| JN896054.1 \| JX094817.1 \| N \|  \| Nat \| \| 508 \| Epipactis phyllanthes \| JN894394.1 \| JN891262.1 \| N \|  \| Nat \| \| 509 \| Epipactis purpurata \| T.S. \| JX094816.1 \| N \|  \| Nat \| \| 510 \| Epipogium aphyllum \| \| T.S. \| N \|  \| Nat \| \| 511 \| Equisetum arvense \| NC_014699.1 \| NC_014699.1 \| N \|  \| Nat \| \| 512 \| Equisetum fluviatile \| T.S. \| AY226142.1 \| N \|  \| Nat \| \| 513 \| Equisetum hyemale \| HF585136.1 \| DQ646001.1 \| N \|  \| Nat \| \| 514 \| Equisetum palustre \| AM883541.1 \| AY226138.1 \| N \|  \| Nat \| \| 515 \| Equisetum pratense \| \| AY226137.1 \| N \|  \| Nat \| \| 516 \| Equisetum ramosissimum \| JF303895.1 \| AB574688.1 \| AN \|  \| Non-inv \| \| 517 \| Equisetum sylvaticum \| AM883553.1 \| AY226136.1 \| N \|  \| Nat \| \| 518 \| Equisetum telmateia \| AM883540.1 \| AF313580.1 \| N \|  \| Nat \| \| 519 \| Equisetum variegatum \| AM883554.1 \| AY226134.1 \| N \|  \| Nat \| \| 520 \| Eranthis hyemalis \| AJ414342.1 \| EU053912.1 \| AN \|  \| Non-inv \| \| 521 \| Erica ciliaris \|  \| T.S. \| N \|  \| Nat \| \| 522 \| Erica cinerea \| JN895360.1 \| JN890834.1 \| N \|  \| Nat \| \| 523 \| Erica tetralix \| U61340.1 \| AF419825.1 \| N \|  \| Nat \| \| 524 \| Erica vagans \| \| T.S. \| N \|  \| Nat \| \| 525 \| Erigeron acer \| JN896122.1 \| JN892440.1 \| N \|  \| Nat \| \| 526 \| Erigeron borealis \| \| T.S. \| N \|  \| Nat \| \| 527 \| Erigeron karvinskianus \| HM850630.1 \| HM849980.1 \| AN \|  \| Non-inv \| \| 528 \| Erinus alpinus \| AY492139.1 \| T.S. \| AN \|  \| Non-inv \| \| 529 \| Eriocaulon aquaticum \| AY952430.1 \| AF036877.1 \| N \|  \| Nat \| \| 530 \| Eriophorum angustifolium \| JN895094.1 \| JX644681.1 \| N \|  \| Nat \| \| 531 \| Eriophorum gracile \| JN895093.1 \| JX644683.1 \| N \|  \| Nat \| \| 532 \| Eriophorum latifolium \| JN895202.1 \| JN892390.1 \| N \|  \| Nat \| \| 533 \| Eriophorum vaginatum \| JN895096.1 \| AM999830.1 \| N \|  \| Nat \| \| 534 \| Erodium cicutarium \| HM850903.1 \| DQ452882.1 \| N \|  \| Nat \| \| 535 \| Erodium lebelii \| JN895638.1 \| JN892228.1 \| N \|  \| Nat \| \| 536 \| Erodium maritimum \| \| JN892233.1 \| N \|  \| Nat \| \| 537 \| Erodium moschatum \| HM850905.1 \| HM849983.1 \| AR \|  \| Non-inv \| \| 538 \| Erophila verna \| HQ619804.1 \| JN892021.1 \| N \|  \| Nat \| \| 539 \| Erucastrum gallicum \| JN584985.1 \| JX520951.1 \| AN \|  \| Non-inv \| \| 540 \| Eryngium campestre \| JN894266.1 \| JN891088.1 \| AR \|  \| Non-inv \| \| 541 \| Eryngium maritimum \| JN895836.1 \| JN891082.1 \| N \|  \| Nat \| \| 542 \| Erysimum cheiranthoides \| JF926663.1 \| JN847823.1 \| AR \|  \| Non-inv \| \| 543 \| Erysimum cheiri \| JN893987.1 \| JN892013.1 \| AR \|  \| Non-inv \| \| 544 \| Euonymus europaeus \| JN895303.1 \| JN892518.1 \| N \|  \| Nat \| \| 545 \| Eupatorium cannabinum \| JN895241.1 \| JN892438.1 \| N \|  \| Nat \| \| 546 \| Euphorbia amygdaloides \| JN894276.1 \| JN893752.1 \| N \|  \| Nat \| \| 547 \| Euphorbia cyparissias \| HQ593293.1 \| HQ590096.1 \| AN \|  \| Non-inv \| \| 548 \| Euphorbia exigua \| JN894771.1 \| HM849987.1 \| AR \|  \| Non-inv \| \| 549 \| Euphorbia helioscopia \| JN894467.1 \| HM849988.1 \| AR \|  \| Non-inv \| \| 550 \| Euphorbia hyberna \| \| T.S. \| N \|  \| Nat \| \| 551 \| Euphorbia lathyris \| JN894776.1 \| HM849989.1 \| AR \|  \| Non-inv \| \| 552 \| Euphorbia paralias \| JN894287.1 \| JN891106.1 \| N \|  \| Nat \| \| 553 \| Euphorbia peplus \| JN895712.1 \| HM849993.1 \| AR \|  \| Non-inv \| \| 554 \| Euphorbia platyphyllos \| JN894608.1 \| JN891554.1 \| AR \|  \| Non-inv \| \| 555 \| Euphorbia portlandica \| JN894291.1 \| JN891857.1 \| N \|  \| Nat \| \| 556 \| Euphrasia anglica \| JN894606.1 \| JN891986.1 \| NE \|  \| Nat \| \| 557 \| Euphrasia arctica \| JN894612.1 \| JN891984.1 \| N \|  \| Nat \| \| 558 \| Euphrasia cambrica \| JN894613.1 \| JN891559.1 \| NE \|  \| Nat \| \| 559 \| Euphrasia confusa \| JN894611.1 \| JN891989.1 \| N \|  \| Nat \| \| 560 \| Euphrasia micrantha \| JN894610.1 \| JN891556.1 \| N \|  \| Nat \| \| 561 \| Euphrasia nemorosa \| JN894615.1 \| JN892210.1 \| N \|  \| Nat \| \| 562 \| Euphrasia officinalis agg. \| JF900571.1 \| T.S. \| N \|  \| Nat \| \| 563 \| Euphrasia ostenfeldii \| JN896182.1 \| JN892212.1 \| N \|  \| Nat \| \| 564 \| Euphrasia pseudokerneri \| JN894614.1 \| JN892213.1 \| NE \|  \| Nat \| \| 565 \| Euphrasia rivularis \| JN895084.1 \| JN892217.1 \| NE \|  \| Nat \| \| 566 \| Euphrasia rostkoviana \| JN894690.1 \| JN892219.1 \| N \|  \| Nat \| \| 567 \| Euphrasia scottica \| JN894422.1 \| JN891474.1 \| N \|  \| Nat \| \| 568 \| Euphrasia tetraquetra \| JN894547.1 \| JN892675.1 \| N \|  \| Nat \| \| 569 \| Fagopyrum esculentum \| AB093087.1 \| JN187116.1 \| AN \|  \| Non-inv \| \| 570 \| Fagus sylvatica \| JN895059.1 \| L13340.2 \| N \|  \| Nat \| \| 571 \| Fallopia convolvulus \| JN893898.1 \| HM850000.1 \| AR \|  \| Inv \| \| 572 \| Fallopia dumetorum \| HM357920.1 \| FM883613.1 \| N \|  \| Nat \| \| 573 \| Fallopia japonica \| EU024772.1 \| AF297131.1 \| AN \| 1 \| Inv \| \| 574 \| Fallopia sachalinensis \| AY042635.1 \| AF297125.1 \| AN \| 1 \| Inv \| \| 575 \| Festuca altissima \| NC_019648.1 \| NC_019648.1 \| N \|  \| Nat \| \| 576 \| Festuca arenaria \| JN894685.1 \| JN891698.1 \| N \|  \| Nat \| \| 577 \| Festuca armoricana \| \| T.S. \| N \|  \| Nat \| \| 578 \| Festuca arundinacea \| NC_011713.2 \| NC_011713.2 \| N \|  \| Nat \| \| 579 \| Festuca filiformis \| JN894715.1 \| JN891700.1 \| N \|  \| Nat \| \| 580 \| Festuca gigantea \| AM234720.1 \| JN891926.1 \| N \|  \| Nat \| \| 581 \| Festuca longifolia \| \| T.S. \| N \|  \| Nat \| \| 582 \| Festuca ovina \| NC_019649.1 \| NC_019649.1 \| N \|  \| Nat \| \| 583 \| Festuca pratensis \| NC_019650.1 \| NC_019650.1 \| N \|  \| Nat \| \| 584 \| Festuca rubra \| DQ786911.1 \| AJ746261.1 \| N \|  \| Nat \| \| 585 \| Festuca vivipara \| JN894537.1 \| JN891472.1 \| N \|  \| Nat \| \| 586 \| Filago gallica \| HM850631.1 \| HM850006.1 \| AR \|  \| Non-inv \| \| 587 \| Filago minima \| JN895247.1 \| JN892444.1 \| N \|  \| Nat \| \| 588 \| Filago pyramidata \| HM445607.1 \| HE963488.1 \| AR \|  \| Non-inv \| \| 589 \| Filago vulgaris \| JN894237.1 \| JN891050.1 \| N \|  \| Nat \| \| 590 \| Filipendula ulmaria \| \| JN892539.1 \| N \|  \| Nat \| \| 591 \| Filipendula vulgaris \| JN894108.1 \| HM850007.1 \| N \|  \| Nat \| \| 592 \| Foeniculum vulgare \| U58563.1 \| GQ120445.1 \| AR \|  \| Non-inv \| \| 593 \| Fragaria vesca \| AF288102.1 \| HM850009.1 \| N \|  \| Nat \| \| 594 \| Frangula alnus \| AY257532.1 \| EU676982.1 \| N \|  \| Nat \| \| 595 \| Frankenia laevis \| AY514853.1 \| JN892243.1 \| N \|  \| Nat \| \| 596 \| Fraxinus excelsior \| AM933427.1 \| FJ862056.1 \| N \|  \| Nat \| \| 597 \| Fuchsia magellanica \| HM851004.1 \| HM850013.1 \| AN \|  \| Non-inv \| \| 598 \| Fumaria bastardii \| JN894936.1 \| JN892008.1 \| N \|  \| Nat \| \| 599 \| Fumaria capreolata \| JN894064.1 \| HM850014.1 \| N \|  \| Nat \| \| 600 \| Fumaria densiflora \| JN894065.1 \| JN892519.1 \| AR \|  \| Non-inv \| \| 601 \| Fumaria muralis \| JN894061.1 \| HM850015.1 \| N \|  \| Nat \| \| 602 \| Fumaria officinalis \| JN895721.1 \| JN893098.1 \| AR \|  \| Non-inv \| \| 603 \| Fumaria purpurea \| JN894853.1 \| JN891895.1 \| NE \|  \| Nat \| \| 604 \| Gagea bohemica \| JN894849.1 \| JN891892.1 \| N \|  \| Nat \| \| 605 \| Gagea lutea \| AB024389.1 \| AB034752.1 \| N \|  \| Nat \| \| 606 \| Galanthus nivalis \| AY101335.1 \| T.S. \| AN \|  \| Non-inv \| \| 607 \| Galega officinalis \| JQ669610.1 \| T.S. \| AN \|  \| Non-inv \| \| 608 \| Galeopsis angustifolia \| JN893863.1 \| JN890575.1 \| AR \|  \| Non-inv \| \| 609 \| Galeopsis bifida \| JN894038.1 \| JN890791.1 \| N \|  \| Nat \| \| 610 \| Galeopsis speciosa \| JN894042.1 \| JN890794.1 \| AR \|  \| Non-inv \| \| 611 \| Galeopsis tetrahit \| JN966309.1 \| JN890796.1 \| N \|  \| Nat \| \| 612 \| Galinsoga parviflora \| FJ697076.1 \| HM850017.1 \| AN \|  \| Non-inv \| \| 613 \| Galinsoga quadriradiata \| HE970705.1 \| GU817762.1 \| AN \|  \| Non-inv \| \| 614 \| Galium aparine \| HQ384560.1 \| DQ006124.1 \| N \|  \| Nat \| \| 615 \| Galium boreale \| HQ593306.1 \| HQ590109.1 \| N \|  \| Nat \| \| 616 \| Galium mollugo \| JN895947.1 \| AY395538.1 \| N \|  \| Nat \| \| 617 \| Galium odoratum \| JN893879.1 \| JN893406.1 \| N \|  \| Nat \| \| 618 \| Galium palustre \| JN893881.1 \| HM850020.1 \| N \|  \| Nat \| \| 619 \| Galium saxatile \| JN895751.1 \| HM850022.1 \| N \|  \| Nat \| \| 620 \| Galium sterneri \| JN894559.1 \| JN893532.1 \| N \|  \| Nat \| \| 621 \| Galium tricornutum \| \| JN893144.1 \| AR \|  \| Non-inv \| \| 622 \| Galium uliginosum \| JN895565.1 \| JN892892.1 \| N \|  \| Nat \| \| 623 \| Galium verum \| HQ593309.1 \| HQ590115.1 \| N \|  \| Nat \| \| 624 \| Gaultheria shallon \| AF124565.1 \| AF124574.1 \| AN \|  \| Inv \| \| 625 \| Genista anglica \| JN894663.1 \| JN891628.1 \| N \|  \| Nat \| \| 626 \| Genista pilosa \| JN894665.1 \| JN893664.1 \| N \|  \| Nat \| \| 627 \| Genista tinctoria \| JN894664.1 \| JN891632.1 \| N \|  \| Nat \| \| 628 \| Gentiana nivalis \| EF552124.1 \|  \| N \|  \| Nat \| \| 629 \| Gentiana pneumonanthe \| JN894544.1 \| JN891473.1 \| N \|  \| Nat \| \| 630 \| Gentiana verna \| EF552104.1 \| DQ660644.1 \| N \|  \| Nat \| \| 631 \| Gentianella amarella \| JN896137.1 \| JN965558.1 \| N \|  \| Nat \| \| 632 \| Gentianella anglica \| JN894540.1 \| JN893666.1 \| NE \|  \| Nat \| \| 633 \| Gentianella campestris \| JN895553.1 \| JN893668.1 \| N \|  \| Nat \| \| 634 \| Gentianella germanica \| AJ406335.1 \| T.S. \| N \|  \| Nat \| \| 635 \| Gentianella uliginosa \| JN896135.1 \| JN892866.1 \| N \|  \| Nat \| \| 636 \| Geranium columbinum \| HE966934.1 \| JN892286.1 \| N \|  \| Nat \| \| 637 \| Geranium dissectum \| HM850907.1 \| HM850025.1 \| AR \|  \| Non-inv \| \| 638 \| Geranium endressii \| \| T.S. \| AN \|  \| Non-inv \| \| 639 \| Geranium lucidum \| JN896161.1 \| JN893709.1 \| N \|  \| Nat \| \| 640 \| Geranium molle \| HM850908.1 \| HM850026.1 \| N \|  \| Nat \| \| 641 \| Geranium phaeum \| \| T.S. \| AN \|  \| Non-inv \| \| 642 \| Geranium pratense \| JN895981.1 \| JN892282.1 \| N \|  \| Nat \| \| 643 \| Geranium purpureum \| JN895982.1 \| HM850027.1 \| N \|  \| Nat \| \| 644 \| Geranium pusillum \| JN895976.1 \| JN893648.1 \| N \|  \| Nat \| \| 645 \| Geranium pyrenaicum \| \| T.S. \| AN \|  \| Non-inv \| \| 646 \| Geranium robertianum \| JN895998.1 \| HQ590118.1 \| N \|  \| Nat \| \| 647 \| Geranium rotundifolium \| HM850910.1 \| HM850028.1 \| N \|  \| Nat \| \| 648 \| Geranium sanguineum \| JN895640.1 \| JN892995.1 \| N \|  \| Nat \| \| 649 \| Geranium sylvaticum \| \| JN893451.1 \| N \|  \| Nat \| \| 650 \| Geum rivale \| JN895978.1 \| JN893446.1 \| N \|  \| Nat \| \| 651 \| Geum urbanum \| JN894110.1 \| JN890884.1 \| N \|  \| Nat \| \| 652 \| Gladiolus communis \| HQ394284.1 \| T.S. \| AN \|  \| Non-inv \| \| 653 \| Gladiolus illyricus \| HQ394320.1 \| HM640542.1 \| N \|  \| Nat \| \| 654 \| Glaucium flavum \| JN895979.1 \| JN893447.1 \| N \|  \| Nat \| \| 655 \| Glaux maritima \| JN895983.1 \| AF213821.1 \| N \|  \| Nat \| \| 656 \| Glechoma hederacea \| AY840143.1 \| AY570384.1 \| N \|  \| Nat \| \| 657 \| Glyceria declinata \| JN895984.1 \| HM850032.1 \| N \|  \| Nat \| \| 658 \| Glyceria fluitans \| JN894745.1 \| HM850033.1 \| N \|  \| Nat \| \| 659 \| Glyceria maxima \| JN895612.1 \| JN891215.1 \| N \|  \| Nat \| \| 660 \| Glyceria notata \| JN895613.1 \| JN892265.1 \| N \|  \| Nat \| \| 661 \| Gnaphalium supinum \| HM445621.1 \| T.S. \| N \|  \| Nat \| \| 662 \| Gnaphalium sylvaticum \| JN895614.1 \| HE574612.1 \| N \|  \| Nat \| \| 663 \| Gnaphalium uliginosum \| HM445642.1 \| JN892964.1 \| N \|  \| Nat \| \| 664 \| Goodyera repens \| T.S. \| FJ571330.1 \| N \|  \| Nat \| \| 665 \| Groenlandia densa \| JN896144.1 \| HQ901571.1 \| N \|  \| Nat \| \| 666 \| Gymnadenia conopsea \| EF612530.1 \| JN890895.1 \| N \|  \| Nat \| \| 667 \| Gymnocarpium dryopteris \| JF832277.1 \| JF832068.1 \| N \|  \| Nat \| \| 668 \| Gymnocarpium robertianum \| \| HQ676504.1 \| N \|  \| Nat \| \| 669 \| Hammarbya paludosa \| JN894395.1 \| JN891265.1 \| N \|  \| Nat \| \| 670 \| Hedera helix \| U58612.1 \| JN892511.1 \| N \|  \| Nat \| \| 671 \| Helianthemum apenninum \| \| T.S. \| N \|  \| Nat \| \| 672 \| Helianthemum nummularium \| JN895671.1 \| FR865140.1 \| N \|  \| Nat \| \| 673 \| Helianthemum oelandicum \| JN895617.1 \| FJ492027.1 \| N \|  \| Nat \| \| 674 \| Helianthus annuus \| AY215805.1 \| AY215124.1 \| AN \|  \| Non-inv \| \| 675 \| Helianthus tuberosus \| AY009464.1 \| GU817765.1 \| AN \|  \| Non-inv \| \| 676 \| Helictotrichon pratense \| JN894523.1 \| JN891446.1 \| N \|  \| Nat \| \| 677 \| Helictotrichon pubescens \| JN894522.1 \| JN891447.1 \| N \|  \| Nat \| \| 678 \| Helleborus foetidus \| AJ414322.1 \| JN893216.1 \| N \|  \| Nat \| \| 679 \| Heracleum sphondylium \| JN894076.1 \| AY395540.1 \| N \|  \| Nat \| \| 680 \| Herminium monorchis \| JN696439.1 \| JN696424.1 \| N \|  \| Nat \| \| 681 \| Herniaria ciliolata \| \| T.S. \| N \|  \| Nat \| \| 682 \| Herniaria glabra \| \| AF132091.1 \| N \|  \| Nat \| \| 683 \| Hesperis matronalis \| HQ593319.1 \| HQ590129.1 \| AN \|  \| Inv \| \| 684 \| Hierochloe odorata \| JN966328.1 \| AJ784828.1 \| N \|  \| Nat \| \| 685 \| Himantoglossum hircinum \| T.S. \| T.S. \| N \|  \| Nat \| \| 686 \| Hippocrepis comosa \| JN894094.1 \| JN893221.1 \| N \|  \| Nat \| \| 687 \| Hippophae rhamnoides \| \| EU371558.1 \| N \|  \| Nat \| \| 688 \| Hippuris vulgaris \| JN895316.1 \| L36443.1 \| N \|  \| Nat \| \| 689 \| Hirschfeldia incana \| JN584990.1 \| T.S. \| AN \|  \| Inv \| \| 690 \| Holcus lanatus \| HE573931.1 \| HM850053.1 \| N \|  \| Nat \| \| 691 \| Holcus mollis \| AM234554.1 \| JN891448.1 \| N \|  \| Nat \| \| 692 \| Honckenya peploides \| JN896056.1 \| JN965588.1 \| N \|  \| Nat \| \| 693 \| Hordelymus europaeus \| AM234596.1 \| EU376158.1 \| N \|  \| Nat \| \| 694 \| Hordeum jubatum \| AB078106.1 \| Z49841.1 \| AN \|  \| Non-inv \| \| 695 \| Hordeum marinum \| FJ897873.1 \| HM850055.1 \| N \|  \| Nat \| \| 696 \| Hordeum murinum \| HF558528.1 \| HM850056.1 \| AR \|  \| Inv \| \| 697 \| Hordeum secalinum \| AB078135.1 \| AY601672.1 \| N \|  \| Nat \| \| 698 \| Hornungia petraea \| JN893991.1 \| JN890732.1 \| N \|  \| Nat \| \| 699 \| Hottonia palustris \| AY647534.1 \| AF395002.1 \| N \|  \| Nat \| \| 700 \| Humulus lupulus \| AY257528.1 \| AF061992.1 \| N \|  \| Nat \| \| 701 \| Huperzia selago \| DQ465962.1 \| DQ464227.1 \| N \|  \| Nat \| \| 702 \| Hyacinthoides hispanica \| \| T.S. \| AN \|  \| Non-inv \| \| 703 \| Hyacinthoides non-scripta \| \| JN892558.1 \| N \|  \| Nat \| \| 704 \| Hydrilla verticillata \| AB002571.1 \| AB004891.1 \| N \|  \| Nat \| \| 705 \| Hydrocharis morsus-ranae \| JN895450.1 \| HQ901567.1 \| N \|  \| Nat \| \| 706 \| Hydrocotyle vulgaris \| DQ133792.1 \| HM850058.1 \| N \|  \| Nat \| \| 707 \| Hymenophyllum tunbrigense \| \| EU553547.1 \| N \|  \| Nat \| \| 708 \| Hymenophyllum wilsonii \| \| GU200669.1 \| N \|  \| Nat \| \| 709 \| Hyoscyamus niger \| EF438829.1 \| HQ216125.1 \| AR \|  \| Non-inv \| \| 710 \| Hypericum androsaemum \| HQ331618.1 \| HQ332070.1 \| N \|  \| Nat \| \| 711 \| Hypericum calycinum \| AB698446.1 \| T.S. \| AN \|  \| Non-inv \| \| 712 \| Hypericum elodes \| HQ331622.1 \| HM850060.1 \| N \|  \| Nat \| \| 713 \| Hypericum hirsutum \| \| JN892838.1 \| N \|  \| Nat \| \| 714 \| Hypericum humifusum \| HM850931.1 \| HM850063.1 \| N \|  \| Nat \| \| 715 \| Hypericum linariifolium \| HQ331628.1 \| HQ332080.1 \| N \|  \| Nat \| \| 716 \| Hypericum maculatum \| \| JN892622.1 \| N \|  \| Nat \| \| 717 \| Hypericum montanum \| \| JN892624.1 \| N \|  \| Nat \| \| 718 \| Hypericum perforatum \| AB698447.1 \| HQ332081.1 \| N \|  \| Nat \| \| 719 \| Hypericum pulchrum \| \| JN892626.1 \| N \|  \| Nat \| \| 720 \| Hypericum tetrapterum \| HQ331631.1 \| HQ332082.1 \| N \|  \| Nat \| \| 721 \| Hypericum undulatum \| HM850935.1 \| HM850067.1 \| N \|  \| Nat \| \| 722 \| Hypochaeris glabra \| JN895228.1 \| HM850068.1 \| N \|  \| Nat \| \| 723 \| Hypochaeris maculata \| JN895229.1 \| JN892419.1 \| N \|  \| Nat \| \| 724 \| Hypochaeris radicata \| JN894186.1 \| AY395542.1 \| N \|  \| Nat \| \| 725 \| Iberis amara \| GQ424589.1 \| FN594828.1 \| N \|  \| Nat \| \| 726 \| Ilex aquifolium \| AF542607.2 \| FJ394589.1 \| N \|  \| Nat \| \| 727 \| Illecebrum verticillatum \| AY514849.1 \| HM850071.1 \| N \|  \| Nat \| \| 728 \| Impatiens capensis \| AJ429280.1 \| Z83142.1 \| AN \|  \| Inv \| \| 729 \| Impatiens glandulifera \| \| T.S. \| AN \| 1 \| Inv \| \| 730 \| Impatiens noli-tangere \| AF542608.2 \| AB043516.1 \| N \|  \| Nat \| \| 731 \| Impatiens parviflora \| T.S. \| T.S. \| AN \|  \| Inv \| \| 732 \| Inula conyzae \| JN895226.1 \| JN892414.1 \| N \|  \| Nat \| \| 733 \| Inula crithmoides \| JN895809.1 \| JN893641.1 \| N \|  \| Nat \| \| 734 \| Inula helenium \| AF151473.1 \| HQ590141.1 \| AR \|  \| Non-inv \| \| 735 \| Inula salicina \| FR865042.1 \| FR865116.1 \| N \|  \| Nat \| \| 736 \| Iris foetidissima \| FJ197278.1 \| HM850074.1 \| N \|  \| Nat \| \| 737 \| Iris germanica \| HM574636.1 \| HM850075.1 \| AN \|  \| Non-inv \| \| 738 \| Iris pseudacorus \| JF972934.1 \| JF972900.1 \| N \|  \| Nat \| \| 739 \| Isatis tinctoria \| JF926669.1 \| JN847841.1 \| AR \|  \| Non-inv \| \| 740 \| Isoetes echinospora \| \| FJ785184.1 \| N \|  \| Nat \| \| 741 \| Isoetes histrix \| T.S. \| AF404497.1 \| N \|  \| Nat \| \| 742 \| Isoetes lacustris \| \| AJ010855.1 \| N \|  \| Nat \| \| 743 \| Isolepis cernua \| JN895309.1 \| HM850076.1 \| N \|  \| Nat \| \| 744 \| Isolepis setacea \| JN895194.1 \| HM850078.1 \| N \|  \| Nat \| \| 745 \| Jasione montana \| EU713247.1 \| EU713354.1 \| N \|  \| Nat \| \| 746 \| Juglans regia \| HE966942.1 \| GQ436392.1 \| AN \|  \| Non-inv \| \| 747 \| Juncus acutiflorus \| JN895315.1 \| JN892527.1 \| N \|  \| Nat \| \| 748 \| Juncus acutus \| JN896001.1 \| AM999842.1 \| N \|  \| Nat \| \| 749 \| Juncus alpinoarticulatus \| \| JN965593.1 \| N \|  \| Nat \| \| 750 \| Juncus ambiguus \| JN894882.1 \| JN892300.1 \| N \|  \| Nat \| \| 751 \| Juncus articulatus \| JN894759.1 \| AY395543.1 \| N \|  \| Nat \| \| 752 \| Juncus balticus \| \| AY216620.1 \| N \|  \| Nat \| \| 753 \| Juncus bufonius \| JN894885.1 \| HM850085.1 \| N \|  \| Nat \| \| 754 \| Juncus bulbosus \| AY973524.1 \| HM850086.1 \| N \|  \| Nat \| \| 755 \| Juncus capitatus \| HM850948.1 \| HM850087.1 \| N \|  \| Nat \| \| 756 \| Juncus castaneus \| \| AY216623.1 \| N \|  \| Nat \| \| 757 \| Juncus compressus \| AY973525.1 \| AY216625.1 \| N \|  \| Nat \| \| 758 \| Juncus conglomeratus \| JN894124.1 \| HM850088.1 \| N \|  \| Nat \| \| 759 \| Juncus effusus \| AB088803.1 \| HQ182434.1 \| N \|  \| Nat \| \| 760 \| Juncus filiformis \| JN895484.1 \| JN892294.1 \| N \|  \| Nat \| \| 761 \| Juncus foliosus \| JN894279.1 \| JN893102.1 \| N \|  \| Nat \| \| 762 \| Juncus gerardii \| JN894675.1 \| AY216613.1 \| N \|  \| Nat \| \| 763 \| Juncus inflexus \| JN895985.1 \| JN893771.1 \| N \|  \| Nat \| \| 764 \| Juncus maritimus \| JN894909.1 \| HM850090.1 \| N \|  \| Nat \| \| 765 \| Juncus squarrosus \| JN895885.1 \| AY216619.1 \| N \|  \| Nat \| \| 766 \| Juncus subnodulosus \| JN896173.1 \| AY216630.1 \| N \|  \| Nat \| \| 767 \| Juncus tenuis \| HM850952.1 \| HM850091.1 \| AN \|  \| Inv \| \| 768 \| Juncus trifidus \| AY973526.1 \| AY216618.1 \| N \|  \| Nat \| \| 769 \| Juncus triglumis \| JN894676.1 \| AY216605.1 \| N \|  \| Nat \| \| 770 \| Juniperus communis \| EU749466.1 \| AY988260.1 \| N \|  \| Nat \| \| 771 \| Kickxia elatine \| JN894549.1 \| HM850096.1 \| AR \|  \| Non-inv \| \| 772 \| Kickxia spuria \| JN894552.1 \| HM850097.1 \| AR \|  \| Non-inv \| \| 773 \| Knautia arvensis \| JN894671.1 \| JN892432.1 \| N \|  \| Nat \| \| 774 \| Kobresia simpliciuscula \| FJ548154.1 \| FJ548284.1 \| N \|  \| Nat \| \| 775 \| Koeleria macrantha \| JN895104.1 \| JN892250.1 \| N \|  \| Nat \| \| 776 \| Koeleria vallesiana \| JX438077.1 \| T.S. \| N \|  \| Nat \| \| 777 \| Koenigia islandica \| EU840455.1 \| EU840287.1 \| N \|  \| Nat \| \| 778 \| Laburnum anagyroides \| FR869998.1 \| Z70077.1 \| AN \|  \| Non-inv \| \| 779 \| Lactuca serriola \| GU109315.1 \| HM850098.1 \| AR \|  \| Inv \| \| 780 \| Lactuca virosa \| JN896266.1 \| JN893836.1 \| N \|  \| Nat \| \| 781 \| Lagarosiphon major \| \| U80703.1 \| AN \|  \| Non-inv \| \| 782 \| Lamiastrum galeobdolon \| JN894213.1 \| HM850102.1 \| N \|  \| Nat \| \| 783 \| Lamium album \| JN894207.1 \| FJ395588.1 \| AR \|  \| Inv \| \| 784 \| Lamium amplexicaule \| JN894206.1 \| AB266225.1 \| AR \|  \| Non-inv \| \| 785 \| Lamium confertum \| JN894205.1 \| JN891286.1 \| AR \|  \| Non-inv \| \| 786 \| Lamium maculatum \| AF531780.1 \| Z37402.1 \| AN \|  \| Non-inv \| \| 787 \| Lamium purpureum \| JF779876.1 \| HM850103.1 \| AR \|  \| Non-inv \| \| 788 \| Larix decidua \| NC_016058.1 \| NC_016058.1 \| AN \|  \| Non-inv \| \| 789 \| Larix kaempferi \| JQ512436.1 \| AB045038.1 \| AN \|  \| Non-inv \| \| 790 \| Lathraea squamaria \| KC542164.1 \| JN890561.1 \| N \|  \| Nat \| \| 791 \| Lathyrus japonicus \| JX505800.1 \| JN661183.1 \| N \|  \| Nat \| \| 792 \| Lathyrus latifolius \| AF522085.1 \| HE963531.1 \| AN \|  \| Non-inv \| \| 793 \| Lathyrus linifolius \| JN894099.1 \| JN892522.1 \| N \|  \| Nat \| \| 794 \| Lathyrus nissolia \| JN895317.1 \| JN892530.1 \| N \|  \| Nat \| \| 795 \| Lathyrus palustris \| JN893916.1 \| JN891868.1 \| N \|  \| Nat \| \| 796 \| Lathyrus pratensis \| JX505811.1 \| AY395544.1 \| N \|  \| Nat \| \| 797 \| Lathyrus sylvestris \| JN894841.1 \| JN890641.1 \| N \|  \| Nat \| \| 798 \| Lathyrus tuberosus \| \| JN661188.1 \| AN \|  \| Non-inv \| \| 799 \| Laurus nobilis \| AF244407.1 \| HM850111.1 \| AN \|  \| Non-inv \| \| 800 \| Lavatera arborea \| EU346779.1 \| T.S. \| N \|  \| Nat \| \| 801 \| Lavatera cretica \| EU346783.1 \|  \| AN \|  \| Non-inv \| \| 802 \| Leersia oryzoides \| FN908060.1 \| FN870396.1 \| N \|  \| Nat \| \| 803 \| Legousia hybrida \| EU713327.1 \| EU713434.1 \| AR \|  \| Non-inv \| \| 804 \| Lemna gibba \| AY034197.1 \| AY034235.1 \| N \|  \| Nat \| \| 805 \| Lemna minor \| NC_010109.1 \| NC_010109.1 \| N \|  \| Nat \| \| 806 \| Lemna minuta \| AY034186.1 \| AY034224.1 \| AN \|  \| Inv \| \| 807 \| Lemna trisulca \| AY034199.1 \| AY034237.1 \| N \|  \| Nat \| \| 808 \| Leontodon autumnalis \| JN894632.1 \| JN890754.1 \| N \|  \| Nat \| \| 809 \| Leontodon hispidus \| JN894007.1 \| AY395545.1 \| N \|  \| Nat \| \| 810 \| Leontodon saxatilis \| JN894008.1 \| HM850115.1 \| N \|  \| Nat \| \| 811 \| Lepidium campestre \| AF144359.1 \| HQ590157.1 \| AR \|  \| Non-inv \| \| 812 \| Lepidium draba \| HM850735.1 \| HM850118.1 \| AN \|  \| Inv \| \| 813 \| Lepidium heterophyllum \| JN894631.1 \| JN891578.1 \| N \|  \| Nat \| \| 814 \| Lepidium latifolium \| JN894164.1 \| JN890954.1 \| N \|  \| Nat \| \| 815 \| Lepidium ruderale \| JN894791.1 \| JF942219.1 \| AR \|  \| Non-inv \| \| 816 \| Leucanthemum vulgare \| HQ593344.1 \| AY395546.1 \| N \|  \| Nat \| \| 817 \| Leucojum aestivum \| AY101314.1 \| T.S. \| N \|  \| Nat \| \| 818 \| Leucojum vernum \| \| T.S. \| AN \|  \| Non-inv \| \| 819 \| Leycesteria formosa \| AF446902.1 \| AJ420872.1 \| AN \|  \| Inv \| \| 820 \| Leymus arenarius \| JN894789.1 \| GU140017.1 \| N \|  \| Nat \| \| 821 \| Ligusticum scoticum \| \| T.S. \| N \|  \| Nat \| \| 822 \| Ligustrum ovalifolium \| JF830536.1 \| HM850124.1 \| AN \|  \| Inv \| \| 823 \| Ligustrum vulgare \| HQ384543.1 \| HQ384901.1 \| N \|  \| Nat \| \| 824 \| Lilium martagon \| AB030872.1 \| T.S. \| AN \|  \| Non-inv \| \| 825 \| Limonium bellidifolium \| \| T.S. \| N \|  \| Nat \| \| 826 \| Limonium binervosum \| \| JN893427.1 \| N \|  \| Nat \| \| 827 \| Limonium humile \| JN894792.1 \| JN893200.1 \| N \|  \| Nat \| \| 828 \| Limonium normannicum \| \| T.S. \| N \|  \| Nat \| \| 829 \| Limonium vulgare \| JN895287.1 \| Y16904.1 \| N \|  \| Nat \| \| 830 \| Limosella aquatica \| FN773544.1 \| JN890684.1 \| N \|  \| Nat \| \| 831 \| Limosella australis \| JN894758.1 \| JN891756.1 \| N \|  \| Nat \| \| 832 \| Linaria pelisseriana \| T.S. \| T.S. \| AN \|  \| Non-inv \| \| 833 \| Linaria purpurea \| \| T.S. \| AN \|  \| Non-inv \| \| 834 \| Linaria repens \| JN894787.1 \| JN891755.1 \| AR \|  \| Non-inv \| \| 835 \| Linaria vulgaris \| HQ593345.1 \| HQ590160.1 \| N \|  \| Nat \| \| 836 \| Linnaea borealis \| AF446911.1 \| AJ428899.1 \| N \|  \| Nat \| \| 837 \| Linum bienne \| HM544102.1 \| FJ169568.1 \| N \|  \| Nat \| \| 838 \| Linum catharticum \| HM544103.1 \| FJ169570.1 \| N \|  \| Nat \| \| 839 \| Linum perenne \| AB038182.1 \| FJ169582.1 \| N \|  \| Nat \| \| 840 \| Linum usitatissimum \| HM544115.1 \| FJ169596.1 \| AN \|  \| Non-inv \| \| 841 \| Liparis loeselii \| AY907157.1 \| JN893838.1 \| N \|  \| Nat \| \| 842 \| Listera cordata \| JN894435.1 \| JN965648.1 \| N \|  \| Nat \| \| 843 \| Listera ovata \| JN896261.1 \| JX051379.1 \| N \|  \| Nat \| \| 844 \| Lithospermum arvense \| EU599676.1 \|  \| AR \|  \| Non-inv \| \| 845 \| Lithospermum officinale \| JN894350.1 \| JN893110.1 \| N \|  \| Nat \| \| 846 \| Lithospermum purpureocaeruleum \| JN895510.1 \| JN892813.1 \| N \|  \| Nat \| \| 847 \| Littorella uniflora \| FN773546.1 \| HM850128.1 \| N \|  \| Nat \| \| 848 \| Lloydia serotina \| JN417355.1 \| Z77294.1 \| N \|  \| Nat \| \| 849 \| Lobelia dortmanna \| JN895292.1 \| DQ356162.1 \| N \|  \| Nat \| \| 850 \| Lobelia urens \| HM850769.1 \| HM850130.1 \| N \|  \| Nat \| \| 851 \| Lobularia maritima \| AP009375.1 \| AP009375.1 \| AN \|  \| Non-inv \| \| 852 \| Loiseleuria procumbens \| U61352.1 \| U49288.1 \| N \|  \| Nat \| \| 853 \| Lolium multiflorum \| NC_019651.1 \| NC_019651.1 \| AN \|  \| Non-inv \| \| 854 \| Lolium perenne \| NC_009950.1 \| NC_009950.1 \| N \|  \| Nat \| \| 855 \| Lolium temulentum \| JN895696.1 \| JN893064.1 \| AR \|  \| Non-inv \| \| 856 \| Lonicera periclymenum \| JN895397.1 \| JN893710.1 \| N \|  \| Nat \| \| 857 \| Lonicera xylosteum \| AM503819.2 \| T.S. \| AN \|  \| Non-inv \| \| 858 \| Lotus angustissimus \| HM851122.1 \| HM850136.1 \| N \|  \| Nat \| \| 859 \| Lotus corniculatus \| HM049505.1 \| HM850139.1 \| N \|  \| Nat \| \| 860 \| Lotus glaber \| JN893921.1 \| JN892506.1 \| N \|  \| Nat \| \| 861 \| Lotus pedunculatus \| JN893890.1 \| HM850142.1 \| N \|  \| Nat \| \| 862 \| Lotus subbiflorus \| JN893889.1 \| JN892504.1 \| N \|  \| Nat \| \| 863 \| Ludwigia palustris \| T.S. \| JX100743.1 \| N \|  \| Nat \| \| 864 \| Lupinus arboreus \| \| Z70054.1 \| AN \|  \| Non-inv \| \| 865 \| Lupinus polyphyllus \| \| Z70052.1 \| AN \|  \| Non-inv \| \| 866 \| Luronium natans \| HQ456465.1 \| U80680.1 \| N \|  \| Nat \| \| 867 \| Luzula arcuata \| \| AY216651.1 \| N \|  \| Nat \| \| 868 \| Luzula campestris \| JN895289.1 \| AY395548.1 \| N \|  \| Nat \| \| 869 \| Luzula forsteri \| JN896075.1 \| JN893716.1 \| N \|  \| Nat \| \| 870 \| Luzula multiflora \| JN895525.1 \| AJ419945.1 \| N \|  \| Nat \| \| 871 \| Luzula pilosa \| JN895350.1 \| AY216653.1 \| N \|  \| Nat \| \| 872 \| Luzula spicata \| \| AY216645.1 \| N \|  \| Nat \| \| 873 \| Luzula sylvatica \| JN896189.1 \| AY216637.1 \| N \|  \| Nat \| \| 874 \| Lychnis alpina \| FJ589569.1 \|  \| N \|  \| Nat \| \| 875 \| Lychnis flos-cuculi \| JN896000.1 \| AY395549.1 \| N \|  \| Nat \| \| 876 \| Lychnis viscaria \| FJ589570.1 \| JN892501.1 \| N \|  \| Nat \| \| 877 \| Lycium \| EF438915.1 \| GQ436614.1 \| AN \|  \| Non-inv \| \| 878 \| Lycium barbarum \| AB036630.1 \| JF942335.1 \| AN \|  \| Non-inv \| \| 879 \| Lycium chinense \| AB036637.1 \| AB051022.1 \| AN \|  \| Non-inv \| \| 880 \| Lycopersicon esculentum \| NC_007898.2 \| NC_007898.2 \| AN \|  \| Non-inv \| \| 881 \| Lycopodiella inundata \| \| Y07938.1 \| N \|  \| Nat \| \| 882 \| Lycopodium annotinum \| \| AJ133247.1 \| N \|  \| Nat \| \| 883 \| Lycopodium clavatum \| \| Y07936.1 \| N \|  \| Nat \| \| 884 \| Lycopus europaeus \| AY840154.1 \| HM850150.1 \| N \|  \| Nat \| \| 885 \| Lysichiton americanus \| AM920549.1 \| AM905728.1 \| AN \|  \| Inv \| \| 886 \| Lysimachia nemorum \| JN895785.1 \| JN891670.1 \| N \|  \| Nat \| \| 887 \| Lysimachia nummularia \| JN895958.1 \| AY395550.1 \| N \|  \| Nat \| \| 888 \| Lysimachia punctata \| \| T.S. \| AN \|  \| Non-inv \| \| 889 \| Lysimachia vulgaris \| JN895201.1 \| AF421095.1 \| N \|  \| Nat \| \| 890 \| Lythrum portula \| HM850988.1 \| HM850155.1 \| N \|  \| Nat \| \| 891 \| Lythrum salicaria \| HQ593354.1 \| AF421496.1 \| N \|  \| Nat \| \| 892 \| Mahonia aquifolium \| HQ619798.1 \| HQ619734.1 \| AN \|  \| Non-inv \| \| 893 \| Malus domestica \| AF309172.1 \| X69750.1 \| AR \|  \| Non-inv \| \| 894 \| Malus sylvestris sens.str. \| JQ391013.1 \| JQ391358.1 \| N \|  \| Nat \| \| 895 \| Malva moschata \| EU346792.1 \| JN891496.1 \| N \|  \| Nat \| \| 896 \| Malva neglecta \| EU346788.1 \| HQ590176.1 \| AR \|  \| Non-inv \| \| 897 \| Malva pusilla \| \| GU981729.1 \| AN \|  \| Non-inv \| \| 898 \| Malva sylvestris \| EU346787.1 \| JN891505.1 \| AR \|  \| Inv \| \| 899 \| Marrubium vulgare \| JN895787.1 \| U28875.1 \| N \|  \| Nat \| \| 900 \| Matricaria discoidea \| HM850671.1 \| HM850160.1 \| AN \|  \| Inv \| \| 901 \| Matricaria recutita \| JN894233.1 \| JN893454.1 \| AR \|  \| Inv \| \| 902 \| Matteuccia struthiopteris \| JF303936.1 \| U62032.1 \| AN \|  \| Non-inv \| \| 903 \| Matthiola incana \| AF144361.1 \| HM850161.1 \| AN \|  \| Non-inv \| \| 904 \| Meconopsis cambrica \| JN895590.1 \| JN634856.1 \| N \|  \| Nat \| \| 905 \| Medicago arabica \| HM159554.1 \| HM850163.1 \| N \|  \| Nat \| \| 906 \| Medicago lupulina \| HM159569.1 \| AY395551.1 \| N \|  \| Nat \| \| 907 \| Medicago minima \| HM159571.1 \| HE963557.1 \| N \|  \| Nat \| \| 908 \| Medicago polymorpha \| AF522104.2 \| HM850165.1 \| N \|  \| Nat \| \| 909 \| Medicago sativa \| AF169289.1 \| Z70173.1 \| N \|  \| Nat \| \| 910 \| Melampyrum arvense \| AM503820.1 \| AM503849.2 \| AN \|  \| Non-inv \| \| 911 \| Melampyrum cristatum \| AM503821.2 \| AM503850.2 \| N \|  \| Nat \| \| 912 \| Melampyrum pratense \| JN894763.1 \| X83721.1 \| N \|  \| Nat \| \| 913 \| Melampyrum sylvaticum \| AF051991.1 \| AM503854.2 \| N \|  \| Nat \| \| 914 \| Melica nutans \| JN895594.1 \| HQ600444.1 \| N \|  \| Nat \| \| 915 \| Melica uniflora \| HE573933.1 \| AJ746263.1 \| N \|  \| Nat \| \| 916 \| Melilotus albus \| AF522110.2 \| HQ590182.1 \| AN \|  \| Non-inv \| \| 917 \| Melilotus altissimus \| JN894066.1 \| JN890824.1 \| AR \|  \| Non-inv \| \| 918 \| Melilotus indicus \| AF522111.2 \| JX505487.1 \| AN \|  \| Non-inv \| \| 919 \| Melilotus officinalis \| HE970723.1 \| JQ933405.1 \| AN \|  \| Inv \| \| 920 \| Melissa officinalis \| HM850795.1 \| HM850169.1 \| AN \|  \| Non-inv \| \| 921 \| Melittis melissophyllum \| JN894404.1 \| JN893655.1 \| N \|  \| Nat \| \| 922 \| Mentha aquatica \| HM850796.1 \| GU344680.1 \| N \|  \| Nat \| \| 923 \| Mentha arvensis \| JN896123.1 \| HQ590183.1 \| N \|  \| Nat \| \| 924 \| Mentha pulegium \| GU381687.1 \| HM850171.1 \| N \|  \| Nat \| \| 925 \| Mentha spicata \| GU381684.1 \| JN891039.1 \| AR \|  \| Non-inv \| \| 926 \| Mentha suaveolens \| GU381685.1 \| U28876.1 \| N \|  \| Nat \| \| 927 \| Menyanthes trifoliata \| EF173062.1 \| L14006.2 \| N \|  \| Nat \| \| 928 \| Mercurialis annua \| HM850922.1 \| HM850173.1 \| AR \|  \| Inv \| \| 929 \| Mercurialis perennis \| JN894824.1 \| AY794944.1 \| N \|  \| Nat \| \| 930 \| Mertensia maritima \| JQ388539.1 \| JN893660.1 \| N \|  \| Nat \| \| 931 \| Mespilus germanica \| DQ860467.1 \| DQ860497.1 \| AR \|  \| Non-inv \| \| 932 \| Meum athamanticum \| JN894474.1 \| JN890689.1 \| N \|  \| Nat \| \| 933 \| Mibora minima \| DQ786927.1 \| JN893345.1 \| N \|  \| Nat \| \| 934 \| Milium effusum \| JN895904 \| HQ600468.1 \| N \|  \| Nat \| \| 935 \| Milium vernale \| JN895904.1 \|  \| N \|  \| Nat \| \| 936 \| Mimulus guttatus \| AY667471.1 \| T.S. \| AN \|  \| Inv \| \| 937 \| Mimulus luteus \| \| T.S. \| AN \|  \| Non-inv \| \| 938 \| Minuartia hybrida \| JN895268.1 \| JN892476.1 \| N \|  \| Nat \| \| 939 \| Minuartia rubella \| JN966374.1 \| JN965675.1 \| N \|  \| Nat \| \| 940 \| Minuartia verna \| JN895264.1 \| JQ933411.1 \| N \|  \| Nat \| \| 941 \| Misopates orontium \| JN894173.1 \| HM850180.1 \| AR \|  \| Non-inv \| \| 942 \| Moehringia trinervia \| AY042615.1 \| JN892472.1 \| N \|  \| Nat \| \| 943 \| Moenchia erecta \| FJ404853.1 \| JN892478.1 \| N \|  \| Nat \| \| 944 \| Molinia caerulea \| HE586092.1 \| AY632367.1 \| N \|  \| Nat \| \| 945 \| Moneses uniflora \| JN966381.1 \| JN965683.1 \| N \|  \| Nat \| \| 946 \| Monotropa hypopitys \| JN895975.1 \| T.S. \| N \|  \| Nat \| \| 947 \| Montia fontana \| JN895972.1 \| JN891226.1 \| N \|  \| Nat \| \| 948 \| Mycelis muralis \| JN895973.1 \| JN891043.1 \| N \|  \| Nat \| \| 949 \| Myosotis alpestris \| AY092860.1 \|  \| N \|  \| Nat \| \| 950 \| Myosotis arvensis \| HM850871.1 \| HM850186.1 \| AR \|  \| Inv \| \| 951 \| Myosotis discolor \| JN895728.1 \| AY395552.1 \| N \|  \| Nat \| \| 952 \| Myosotis laxa \| JN895883.1 \| HQ590187.1 \| N \|  \| Nat \| \| 953 \| Myosotis ramosissima \| JN894440.1 \| JN891320.1 \| N \|  \| Nat \| \| 954 \| Myosotis scorpioides \| JN895929.1 \| GU344681.1 \| N \|  \| Nat \| \| 955 \| Myosotis secunda \| JN895415.1 \| HM850189.1 \| N \|  \| Nat \| \| 956 \| Myosotis sicula \| EU599672.1 \| EU599848.1 \| N \|  \| Nat \| \| 957 \| Myosotis stolonifera \| HM850875.1 \| HM850190.1 \| N \|  \| Nat \| \| 958 \| Myosotis sylvatica \| FJ395375.1 \| HQ590188.1 \| N \|  \| Nat \| \| 959 \| Myosoton aquaticum \| JN894058.1 \| JN893174.1 \| N \|  \| Nat \| \| 960 \| Myrica gale \| AY191715.1 \| JN965685.1 \| N \|  \| Nat \| \| 961 \| Myriophyllum alterniflorum \| EF178978.1 \| HM850192.1 \| N \|  \| Nat \| \| 962 \| Myriophyllum aquaticum \| AY335978.1 \| JX100757.1 \| AN \| 1 \| Inv \| \| 963 \| Myriophyllum spicatum \| EF178976.1 \| GU344679.1 \| N \|  \| Nat \| \| 964 \| Myriophyllum verticillatum \| EF178983.1 \| GU344678.1 \| N \|  \| Nat \| \| 965 \| Myrrhis odorata \| U58569.1 \| T.S. \| AN \|  \| Inv \| \| 966 \| Najas flexilis \| HM240461.1 \| HQ901569.1 \| N \|  \| Nat \| \| 967 \| Najas marina \| HM240477.1 \| U80705.1 \| N \|  \| Nat \| \| 968 \| Narcissus pseudonarcissus \| JX464573.1 \| JN890813.1 \| N \|  \| Nat \| \| 969 \| Nardus stricta \| EU434289.1 \| HM850196.1 \| N \|  \| Nat \| \| 970 \| Narthecium ossifragum \| AB679482.1 \| AY149348.1 \| N \|  \| Nat \| \| 971 \| Neottia nidus-avis \| \| JN893608.1 \| N \|  \| Nat \| \| 972 \| Nepeta cataria \| HQ593369.1 \| JX520953.1 \| AR \|  \| Non-inv \| \| 973 \| Nuphar lutea \| AF117100.1 \| DQ182338.1 \| N \|  \| Nat \| \| 974 \| Nuphar pumila \| AF117088.1 \|  \| N \|  \| Nat \| \| 975 \| Nymphaea alba \| NC_006050.1 \| NC_006050.1 \| N \|  \| Nat \| \| 976 \| Nymphoides peltata \| JF926442.1 \| EF173110.1 \| N \|  \| Nat \| \| 977 \| Odontites vernus \| JN894169.1 \| JN890584.1 \| N \|  \| Nat \| \| 978 \| Oenanthe aquatica \| JN893874.1 \| JN890585.1 \| N \|  \| Nat \| \| 979 \| Oenanthe crocata \| JN894482.1 \| JN892220.1 \| N \|  \| Nat \| \| 980 \| Oenanthe fistulosa \| JN893875.1 \| JN890586.1 \| N \|  \| Nat \| \| 981 \| Oenanthe lachenalii \| JN895124.1 \| JN892758.1 \| N \|  \| Nat \| \| 982 \| Oenanthe pimpinelloides \| JN895125.1 \| JN892280.1 \| N \|  \| Nat \| \| 983 \| Oenothera biennis \| NC_010361.1 \| NC_010361.1 \| AN \|  \| Non-inv \| \| 984 \| Oenothera glazioviana \| NC_010360.1 \| NC_010360.1 \| AN \|  \| Inv \| \| 985 \| Ononis reclinata \| JN895657.1 \| JN890825.1 \| N \|  \| Nat \| \| 986 \| Ononis repens \| JN894068.1 \| JN892992.1 \| N \|  \| Nat \| \| 987 \| Ononis spinosa \| JN894101.1 \| JN890828.1 \| N \|  \| Nat \| \| 988 \| Onopordum acanthium \| JN894100.1 \| JN891890.1 \| AR \|  \| Non-inv \| \| 989 \| Ophioglossum lusitanicum \| \| DQ646002.1 \| N \|  \| Nat \| \| 990 \| Ophioglossum vulgatum \| AB716735.1 \| AY138414.1 \| N \|  \| Nat \| \| 991 \| Ophrys apifera \| JN893962.1 \| JN890698.1 \| N \|  \| Nat \| \| 992 \| Ophrys fuciflora \| GQ248170.1 \| GQ248658.1 \| N \|  \| Nat \| \| 993 \| Ophrys insectifera \| JN894888.1 \| JN891942.1 \| N \|  \| Nat \| \| 994 \| Orchis laxiflora \| T.S. \| T.S. \| N \|  \| Nat \| \| 995 \| Orchis mascula \| JN896032.1 \| JN893527.1 \| N \|  \| Nat \| \| 996 \| Orchis militaris \| T.S. \| T.S. \| N \|  \| Nat \| \| 997 \| Orchis morio \| T.S. \| T.S. \| N \|  \| Nat \| \| 998 \| Orchis purpurea \| \| T.S. \| N \|  \| Nat \| \| 999 \| Orchis simia \| T.S. \| T.S. \| N \|  \| Nat \| \| 1000 \| Orchis ustulata \| \| T.S. \| N \|  \| Nat \| \| 1001 \| Oreopteris limbosperma \| \| HQ676506.1 \| N \|  \| Nat \| \| 1002 \| Origanum vulgare \| AY840165.1 \| HM850213.1 \| N \|  \| Nat \| \| 1003 \| Ornithogalum angustifolium \| \| T.S. \| AN \|  \| Non-inv \| \| 1004 \| Ornithogalum pyrenaicum \| \| AM992655.2 \| N \|  \| Nat \| \| 1005 \| Ornithopus perpusillus \| JN894070.1 \| HM850216.1 \| N \|  \| Nat \| \| 1006 \| Ornithopus pinnatus \| HM851145.1 \| HM850217.1 \| N \|  \| Nat \| \| 1007 \| Orobanche artemisiae-campestris \| \| AY582184.1 \| N \|  \| Nat \| \| 1008 \| Orobanche caryophyllacea \| AF051992.1 \| AY582187.1 \| N \|  \| Nat \| \| 1009 \| Orobanche elatior \| JN894103.1 \| JN890873.1 \| N \|  \| Nat \| \| 1010 \| Orobanche hederae \| AF051995.1 \| HE963574.1 \| N \|  \| Nat \| \| 1011 \| Orobanche minor \| JN895235.1 \|  \| N \|  \| Nat \| \| 1012 \| Orobanche purpurea \| \| JN892425.1 \| N \|  \| Nat \| \| 1013 \| Orobanche rapum-genistae \| JN893918.1 \|  \| N \|  \| Nat \| \| 1014 \| Orthilia secunda \| JN894293.1 \| AF419838.1 \| N \|  \| Nat \| \| 1015 \| Osmunda regalis \| HF585137.1 \| AB024948.1 \| N \|  \| Nat \| \| 1016 \| Oxalis acetosella \| JN895689.1 \| FJ670181.1 \| N \|  \| Nat \| \| 1017 \| Oxalis articulata \| HM851016.1 \| HM850220.1 \| AN \|  \| Non-inv \| \| 1018 \| Oxalis corniculata \| AB233839.1 \| AB233943.1 \| AN \|  \| Inv \| \| 1019 \| Oxalis debilis \| HM851018.1 \| HM850222.1 \| AN \|  \| Non-inv \| \| 1020 \| Oxalis exilis \|  \| T.S. \| AN \|  \| Inv \| \| 1021 \| Oxalis incarnata \| HQ619822.1 \| HQ619761.1 \| AN \|  \| Non-inv \| \| 1022 \| Oxalis latifolia \| EU002186.1 \| EU002282.1 \| AN \|  \| Non-inv \| \| 1023 \| Oxalis pes-caprae \| HM851020.1 \| AM235044.1 \| AN \|  \| Non-inv \| \| 1024 \| Oxalis stricta \| AY935936.1 \| KC481641.1 \| AN \|  \| Non-inv \| \| 1025 \| Oxyria digyna \| EU840459.1 \| JF942751.1 \| N \|  \| Nat \| \| 1026 \| Oxytropis campestris \| JQ669616.1 \| T.S. \| N \|  \| Nat \| \| 1027 \| Papaver argemone \| JN893922.1 \| JN890644.1 \| AR \|  \| Non-inv \| \| 1028 \| Papaver dubium \| JN893923.1 \| HM850229.1 \| AR \|  \| Inv \| \| 1029 \| Papaver hybridum \| JN896018.1 \| JN893380.1 \| AR \|  \| Non-inv \| \| 1030 \| Papaver rhoeas \| FJ626525.1 \| FJ626614.1 \| AR \|  \| Inv \| \| 1031 \| Papaver somniferum \| JN895410.1 \| HM850232.1 \| AR \|  \| Inv \| \| 1032 \| Parapholis incurva \| DQ786931.1 \| EF125154.1 \| N \|  \| Nat \| \| 1033 \| Parapholis strigosa \| JN896014.1 \| JN893606.1 \| N \|  \| Nat \| \| 1034 \| Parentucellia viscosa \| AY849606.1 \| AY849865.1 \| N \|  \| Nat \| \| 1035 \| Parietaria judaica \| JN896013.1 \| HM850236.1 \| N \|  \| Nat \| \| 1036 \| Paris quadrifolia \| JN417382.1 \| JN417472.1 \| N \|  \| Nat \| \| 1037 \| Parnassia palustris \| JN896020.1 \| AY935731.1 \| N \|  \| Nat \| \| 1038 \| Pastinaca sativa \| JN895118.1 \| JN893745.1 \| N \|  \| Nat \| \| 1039 \| Pedicularis palustris \| JN896186.1 \| JN893744.1 \| N \|  \| Nat \| \| 1040 \| Pedicularis sylvatica \| AF531781.1 \| JN891318.1 \| N \|  \| Nat \| \| 1041 \| Pentaglottis sempervirens \| \| T.S. \| AN \|  \| Inv \| \| 1042 \| Persicaria amphibia \| KC342453.1 \| AY395553.1 \| N \|  \| Nat \| \| 1043 \| Persicaria bistorta \| JN896187.1 \| JN893814.1 \| N \|  \| Nat \| \| 1044 \| Persicaria hydropiper \| HM357924.1 \| EF653779.1 \| N \|  \| Nat \| \| 1045 \| Persicaria lapathifolia \| HM357922.1 \| HM357899.1 \| N \|  \| Nat \| \| 1046 \| Persicaria maculosa \| EU749345.1 \| HQ435350.1 \| N \|  \| Nat \| \| 1047 \| Persicaria minor \| JN896184.1 \| FM883633.1 \| N \|  \| Nat \| \| 1048 \| Persicaria mitis \| JN896240.1 \| JN893822.1 \| N \|  \| Nat \| \| 1049 \| Persicaria vivipara \| EU840456.1 \| FM883608.1 \| N \|  \| Nat \| \| 1050 \| Persicaria wallichii \| \| FM883640.1 \| AN \|  \| Non-inv \| \| 1051 \| Petasites albus \| GU817504.1 \| T.S. \| AN \|  \| Non-inv \| \| 1052 \| Petasites fragrans \| \| HM850247.1 \| AN \|  \| Inv \| \| 1053 \| Petasites hybridus \| JN895682.1 \| JN892562.1 \| N \|  \| Nat \| \| 1054 \| Petrorhagia prolifera \| \| T.S. \| AN \|  \| Non-inv \| \| 1055 \| Petroselinum crispum \| U58575.1 \| HM850248.1 \| AR \|  \| Non-inv \| \| 1056 \| Petroselinum segetum \| JN894984.1 \| JN892076.1 \| N \|  \| Nat \| \| 1057 \| Peucedanum ostruthium \| \| JN892079.1 \| AR \|  \| Non-inv \| \| 1058 \| Peucedanum palustre \| \| T.S. \| N \|  \| Nat \| \| 1059 \| Phalaris arundinacea \| AF164396.1 \| AJ784827.1 \| N \|  \| Nat \| \| 1060 \| Phalaris canariensis \| JX438068.1 \| T.S. \| AN \|  \| Non-inv \| \| 1061 \| Phalaris minor \| \| T.S. \| AN \|  \| Non-inv \| \| 1062 \| Phegopteris connectilis \| JF303932.1 \| JF832080.1 \| N \|  \| Nat \| \| 1063 \| Phleum alpinum \| T.S. \| T.S. \| N \|  \| Nat \| \| 1064 \| Phleum arenarium \| JN895255.1 \| JN893617.1 \| N \|  \| Nat \| \| 1065 \| Phleum bertolonii \| JN894629.1 \| JN893804.1 \| N \|  \| Nat \| \| 1066 \| Phleum phleoides \| AM234552.1 \| T.S. \| N \|  \| Nat \| \| 1067 \| Phleum pratense \| AF164397.1 \| HM850252.1 \| N \|  \| Nat \| \| 1068 \| Phragmites australis \| JN894196.1 \| EF423005.1 \| N \|  \| Nat \| \| 1069 \| Phyllitis scolopendrium \| \| AF240645.1 \| N \|  \| Nat \| \| 1070 \| Phyllodoce caerulea \| U61318.1 \| AF419829.1 \| N \|  \| Nat \| \| 1071 \| Physospermum cornubiense \| \| T.S. \| N \|  \| Nat \| \| 1072 \| Phyteuma orbiculare \| \| T.S. \| N \|  \| Nat \| \| 1073 \| Picea abies \| AY289610.1 \| JQ512566.1 \| AN \|  \| Non-inv \| \| 1074 \| Picea sitchensis \| NC_011152.3 \| NC_011152.3 \| AN \|  \| Non-inv \| \| 1075 \| Picris echioides \| JN896098.1 \| HM850259.1 \| AR \|  \| Inv \| \| 1076 \| Picris hieracioides \| JN896100.1 \| JN890580.1 \| N \|  \| Nat \| \| 1077 \| Pilosella aurantiaca \| HQ593320.1 \| HQ590130.1 \| AN \|  \| Non-inv \| \| 1078 \| Pilosella officinarum \| JN895044.1 \| HQ590133.1 \| N \|  \| Nat \| \| 1079 \| Pilosella peleteriana \| JN894562.1 \| JN893548.1 \| N \|  \| Nat \| \| 1080 \| Pilularia globulifera \| T.S. \| AY612681.1 \| N \|  \| Nat \| \| 1081 \| Pimpinella saxifraga \| U58576.1 \| U50229.1 \| N \|  \| Nat \| \| 1082 \| Pinguicula grandiflora \| AF531791.1 \| AF482525.1 \| N \|  \| Nat \| \| 1083 \| Pinguicula lusitanica \| DQ010661.1 \| JN893189.1 \| N \|  \| Nat \| \| 1084 \| Pinguicula vulgaris \| AF531807.1 \| JN965730.1 \| N \|  \| Nat \| \| 1085 \| Pinus contorta \| AB080921.1 \| NC_011153.4 \| AN \|  \| Non-inv \| \| 1086 \| Pinus nigra \| JQ512469.1 \| JQ512593.1 \| AN \|  \| Non-inv \| \| 1087 \| Pinus sylvestris \| AB097781.1 \| AB019809.1 \| N \|  \| Nat \| \| 1088 \| Plantago coronopus \| JN895151.1 \| HM850263.1 \| N \|  \| Nat \| \| 1089 \| Plantago lanceolata \| JN895253.1 \| L36454.1 \| N \|  \| Nat \| \| 1090 \| Plantago major \| JN895254.1 \| HM850266.1 \| N \|  \| Nat \| \| 1091 \| Plantago maritima \| JN966426.1 \| JN965733.1 \| N \|  \| Nat \| \| 1092 \| Plantago media \| AY667474.1 \| AJ389596.1 \| N \|  \| Nat \| \| 1093 \| Platanthera bifolia \| T.S. \| T.S. \| N \|  \| Nat \| \| 1094 \| Platanthera chlorantha \| EF612531.1 \| JN891570.1 \| N \|  \| Nat \| \| 1095 \| Poa alpina \| DQ786933.1 \| JN965738.1 \| N \|  \| Nat \| \| 1096 \| Poa angustifolia \| JN894113.1 \| JN893843.1 \| N \|  \| Nat \| \| 1097 \| Poa annua \| FN908068.1 \| FN870404.1 \| N \|  \| Nat \| \| 1098 \| Poa bulbosa \| AM234594.1 \| JN892190.1 \| N \|  \| Nat \| \| 1099 \| Poa chaixii \|  \| T.S. \| AN \|  \| Non-inv \| \| 1100 \| Poa compressa \| EU749333.1 \| EU676942.1 \| N \|  \| Nat \| \| 1101 \| Poa glauca \| JN894112.1 \| JN893698.1 \| N \|  \| Nat \| \| 1102 \| Poa humilis \| JN894299.1 \| JN893728.1 \| N \|  \| Nat \| \| 1103 \| Poa infirma \| JX438058.1 \|  \| N \|  \| Nat \| \| 1104 \| Poa nemoralis \| JN894298.1 \| JN892396.1 \| N \|  \| Nat \| \| 1105 \| Poa palustris \| FN668472.1 \| JN965750.1 \| AN \|  \| Non-inv \| \| 1106 \| Poa pratensis sens.lat. \| AF164402.1 \| HQ600438.1 \| N \|  \| Nat \| \| 1107 \| Poa trivialis \| JN895206.1 \| HM850270.1 \| N \|  \| Nat \| \| 1108 \| Polemonium caeruleum \| EU628512.1 \| T.S. \| N \|  \| Nat \| \| 1109 \| Polygala calcarea \| \| AM234194.1 \| N \|  \| Nat \| \| 1110 \| Polygala serpyllifolia \| \| EU644685.1 \| N \|  \| Nat \| \| 1111 \| Polygala vulgaris \| JN894300.1 \| AM234193.1 \| N \|  \| Nat \| \| 1112 \| Polygonatum multiflorum \| EF133696.1 \| JN891841.1 \| N \|  \| Nat \| \| 1113 \| Polygonatum odoratum \| AB017316.1 \| JN893756.1 \| N \|  \| Nat \| \| 1114 \| Polygonatum verticillatum \| JF955582.1 \| JF943497.1 \| N \|  \| Nat \| \| 1115 \| Polygonum arenastrum \| JN895284.1 \| JN893586.1 \| AR \|  \| Non-inv \| \| 1116 \| Polygonum aviculare \| HM357913.1 \| HM850273.1 \| N \|  \| Nat \| \| 1117 \| Polygonum maritimum \| HM851079.1 \| HM850274.1 \| N \|  \| Nat \| \| 1118 \| Polygonum oxyspermum \| JN894295.1 \| JN893596.1 \| N \|  \| Nat \| \| 1119 \| Polygonum rurivagum \| JN894296.1 \| FM883642.1 \| AR \|  \| Non-inv \| \| 1120 \| Polypodium interjectum \| \| HE963606.1 \| N \|  \| Nat \| \| 1121 \| Polypodium vulgare \| \| JF832081.1 \| N \|  \| Nat \| \| 1122 \| Polypogon monspeliensis \| DQ786937.1 \| HM850275.1 \| N \|  \| Nat \| \| 1123 \| Polystichum aculeatum \| \| HQ676509.1 \| N \|  \| Nat \| \| 1124 \| Polystichum lonchitis \| \| AF537247.1 \| N \|  \| Nat \| \| 1125 \| Polystichum setiferum \| \| AF537254.1 \| N \|  \| Nat \| \| 1126 \| Populus alba \| AP008956.1 \| AP008956.1 \| AN \|  \| Inv \| \| 1127 \| Populus nigra sens.lat. \| AB038186.1 \| HM850278.1 \| N \|  \| Nat \| \| 1128 \| Populus tremula \| AJ506087.1 \| AJ418827.1 \| N \|  \| Nat \| \| 1129 \| Potamogeton alpinus \| JN894621.1 \| AB196845.1 \| N \|  \| Nat \| \| 1130 \| Potamogeton berchtoldii \| JN894618.1 \| FJ956852.1 \| N \|  \| Nat \| \| 1131 \| Potamogeton coloratus \| JN895690.1 \| GU344673.1 \| N \|  \| Nat \| \| 1132 \| Potamogeton compressus \| JN894782.1 \| AB196846.1 \| N \|  \| Nat \| \| 1133 \| Potamogeton crispus \| JN894781.1 \| U80722.1 \| N \|  \| Nat \| \| 1134 \| Potamogeton filiformis \| JN894783.1 \| FJ956863.1 \| N \|  \| Nat \| \| 1135 \| Potamogeton friesii \| JN894784.1 \| JN891792.1 \| N \|  \| Nat \| \| 1136 \| Potamogeton gramineus \| \| U80723.1 \| N \|  \| Nat \| \| 1137 \| Potamogeton lucens \| JF955617.1 \| DQ859173.1 \| N \|  \| Nat \| \| 1138 \| Potamogeton natans \| JN894670.1 \| AB196946.1 \| N \|  \| Nat \| \| 1139 \| Potamogeton nodosus \| HM851047.1 \| HM850281.1 \| N \|  \| Nat \| \| 1140 \| Potamogeton obtusifolius \| JN894667.1 \| AB196947.1 \| N \|  \| Nat \| \| 1141 \| Potamogeton pectinatus \| JN894668.1 \| AB196953.1 \| N \|  \| Nat \| \| 1142 \| Potamogeton perfoliatus \| JN895042.1 \| EU741052.1 \| N \|  \| Nat \| \| 1143 \| Potamogeton polygonifolius \| JN896105.1 \| HM850282.1 \| N \|  \| Nat \| \| 1144 \| Potamogeton praelongus \| \| AB196952.1 \| N \|  \| Nat \| \| 1145 \| Potamogeton pusillus \| HM851049.1 \| HM850283.1 \| N \|  \| Nat \| \| 1146 \| Potamogeton trichoides \| JN893850.1 \| JN890548.1 \| N \|  \| Nat \| \| 1147 \| Potentilla anglica \| JN896009.1 \| HM850284.1 \| N \|  \| Nat \| \| 1148 \| Potentilla anserina \| AF288113.1 \| JN893768.1 \| N \|  \| Nat \| \| 1149 \| Potentilla argentea \| \| T.S. \| N \|  \| Nat \| \| 1150 \| Potentilla crantzii \| JN896011.1 \| JN893502.1 \| N \|  \| Nat \| \| 1151 \| Potentilla erecta \| HM850688.1 \| HM850285.1 \| N \|  \| Nat \| \| 1152 \| Potentilla fruticosa \| GQ434189.1 \| JF941363.1 \| N \|  \| Nat \| \| 1153 \| Potentilla neumanniana \| JN896181.1 \| JN893739.1 \| N \|  \| Nat \| \| 1154 \| Potentilla palustris \| JN896178.1 \| JN890807.1 \| N \|  \| Nat \| \| 1155 \| Potentilla reptans \| HM850690.1 \| HM850287.1 \| N \|  \| Nat \| \| 1156 \| Potentilla rupestris \| JN893882.1 \| JN892820.1 \| N \|  \| Nat \| \| 1157 \| Potentilla sterilis \| JN895651.1 \| JN893010.1 \| N \|  \| Nat \| \| 1158 \| Primula elatior \| DQ378361.1 \| T.S. \| N \|  \| Nat \| \| 1159 \| Primula farinosa \| DQ378345.1 \|  \| N \|  \| Nat \| \| 1160 \| Primula scotica \| \| T.S. \| NE \|  \| Nat \| \| 1161 \| Primula veris \| AY647530.1 \| AF394981.1 \| N \|  \| Nat \| \| 1162 \| Primula vulgaris \| DQ378362.1 \| JN891810.1 \| N \|  \| Nat \| \| 1163 \| Prunella vulgaris \| FJ395426.1 \| AY395556.1 \| N \|  \| Nat \| \| 1164 \| Prunus avium \| JN895117.1 \| HQ235393.1 \| N \|  \| Nat \| \| 1165 \| Prunus cerasifera \| HQ619834.1 \| AF227900.1 \| AN \|  \| Non-inv \| \| 1166 \| Prunus cerasus \| JN894830.1 \| HQ235416.1 \| AR \|  \| Non-inv \| \| 1167 \| Prunus domestica \| JN894829.1 \| HQ235429.1 \| AR \|  \| Non-inv \| \| 1168 \| Prunus laurocerasus \| AF288116.1 \| U06809.1 \| AN \|  \| Inv \| \| 1169 \| Prunus lusitanica \| HQ235182.1 \| HQ235466.1 \| AN \|  \| Non-inv \| \| 1170 \| Prunus padus \| JN895843.1 \| HQ235500.1 \| N \|  \| Nat \| \| 1171 \| Prunus spinosa \| JN895321.1 \| AF227904.1 \| N \|  \| Nat \| \| 1172 \| Pseudofumaria lutea \| \| T.S. \| AN \|  \| Inv \| \| 1173 \| Pseudorchis albida \| \| JN892451.1 \| N \|  \| Nat \| \| 1174 \| Pseudotsuga menziesii \| AF143439.1 \| AY664856.1 \| AN \|  \| Non-inv \| \| 1175 \| Pteridium aquilinum \| FR865060.1 \| AY300097.1 \| N \|  \| Nat \| \| 1176 \| Puccinellia distans \| DQ786938.1 \| L14621.1 \| N \|  \| Nat \| \| 1177 \| Puccinellia fasciculata \| AM234588.1 \| JN892615.1 \| N \|  \| Nat \| \| 1178 \| Puccinellia maritima \| JN895281.1 \| JN892616.1 \| N \|  \| Nat \| \| 1179 \| Puccinellia rupestris \| JN895275.1 \| JN892483.1 \| N \|  \| Nat \| \| 1180 \| Pulicaria dysenterica \| JN894563.1 \| JN891494.1 \| N \|  \| Nat \| \| 1181 \| Pulmonaria obscura \| EU599700.1 \| EU599876.1 \| N \|  \| Nat \| \| 1182 \| Pulmonaria officinalis \| HQ619799.1 \| HQ619735.1 \| AN \|  \| Non-inv \| \| 1183 \| Pulsatilla vulgaris \| \| T.S. \| N \|  \| Nat \| \| 1184 \| Pyrola minor \| JN894990.1 \| JN891118.1 \| N \|  \| Nat \| \| 1185 \| Pyrola rotundifolia \| U61328.1 \| L12622.2 \| N \|  \| Nat \| \| 1186 \| Pyrus communis sens.str. \| JQ391044.1 \| JQ391382.1 \| AR \|  \| Non-inv \| \| 1187 \| Quercus cerris \| AB125034.1 \| AB125017.1 \| AN \|  \| Inv \| \| 1188 \| Quercus ilex \| AB125037.1 \| AB125020.1 \| AN \|  \| Inv \| \| 1189 \| Quercus petraea \| AB125041.1 \| AB125024.1 \| N \|  \| Nat \| \| 1190 \| Quercus robur \| AB125042.1 \| AB125025.1 \| N \|  \| Nat \| \| 1191 \| Radiola linoides \| HM544118.1 \| FJ169598.1 \| N \|  \| Nat \| \| 1192 \| Ranunculus acris \| AY954199.1 \| AY395557.1 \| N \|  \| Nat \| \| 1193 \| Ranunculus aquatilis \| JN893994.1 \| JN965798.1 \| N \|  \| Nat \| \| 1194 \| Ranunculus arvensis \| HQ650551.1 \| JN892029.1 \| AR \|  \| Non-inv \| \| 1195 \| Ranunculus auricomus \| FM242739.1 \| JN893758.1 \| N \|  \| Nat \| \| 1196 \| Ranunculus baudotii \| JN895830.1 \| JN891012.1 \| N \|  \| Nat \| \| 1197 \| Ranunculus bulbosus \| HQ619831.1 \| HM850293.1 \| N \|  \| Nat \| \| 1198 \| Ranunculus circinatus \| HQ894448.1 \| GU344677.1 \| N \|  \| Nat \| \| 1199 \| Ranunculus ficaria \| JN895319.1 \| EU053919.1 \| N \|  \| Nat \| \| 1200 \| Ranunculus flammula \| AY954204.1 \| HM850295.1 \| N \|  \| Nat \| \| 1201 \| Ranunculus fluitans \| AY954129.1 \| JN891886.1 \| N \|  \| Nat \| \| 1202 \| Ranunculus hederaceus \| JN895320.1 \| JN892536.1 \| N \|  \| Nat \| \| 1203 \| Ranunculus lingua \| AY954206.1 \| JN892742.1 \| N \|  \| Nat \| \| 1204 \| Ranunculus muricatus \| AY954191.1 \| HM850296.1 \| AN \|  \| Non-inv \| \| 1205 \| Ranunculus omiophyllus \| JN895679.1 \| JN893041.1 \| N \|  \| Nat \| \| 1206 \| Ranunculus ophioglossifolius \| AY954207.1 \|  \| N \|  \| Nat \| \| 1207 \| Ranunculus paludosus \| AY954155.1 \|  \| N \|  \| Nat \| \| 1208 \| Ranunculus parviflorus \| AY954202.1 \| HM850297.1 \| N \|  \| Nat \| \| 1209 \| Ranunculus peltatus \| JN894996.1 \| JN892102.1 \| N \|  \| Nat \| \| 1210 \| Ranunculus penicillatus \| JN894289.1 \| JN892100.1 \| N \|  \| Nat \| \| 1211 \| Ranunculus repens \| AY954182.1 \| HM850298.1 \| N \|  \| Nat \| \| 1212 \| Ranunculus reptans \| AY954205.1 \|  \| N \|  \| Nat \| \| 1213 \| Ranunculus sceleratus \| GU257993.1 \| AB517148.1 \| N \|  \| Nat \| \| 1214 \| Ranunculus trichophyllus \| AY954133.1 \| EU053922.1 \| N \|  \| Nat \| \| 1215 \| Ranunculus tripartitus \| JN895146.1 \| JN892310.1 \| N \|  \| Nat \| \| 1216 \| Raphanus raphanistrum \| JN584998.1 \| HM850299.1 \| N \|  \| Nat \| \| 1217 \| Reseda luteola \| FJ212206.1 \| HM850302.1 \| AR \|  \| Inv \| \| 1218 \| Rhamnus cathartica \| AY257533.1 \| L13189.2 \| N \|  \| Nat \| \| 1219 \| Rhinanthus minor \| KC542182.1 \| AY395558.1 \| N \|  \| Nat \| \| 1220 \| Rhododendron ponticum \| AB012732.1 \| T.S. \| AN \| 1 \| Inv \| \| 1221 \| Rhynchospora alba \| JN894695.1 \| AM999856.1 \| N \|  \| Nat \| \| 1222 \| Rhynchospora fusca \| \| AM999859.1 \| N \|  \| Nat \| \| 1223 \| Ribes alpinum \| HQ593410.1 \| T.S. \| N \|  \| Nat \| \| 1224 \| Ribes nigrum \| HE967476.1 \| HE963630.1 \| AN \|  \| Non-inv \| \| 1225 \| Ribes spicatum \| \| T.S. \| N \|  \| Nat \| \| 1226 \| Ribes uva-crispa \| T.S. \| T.S. \| AN \|  \| Non-inv \| \| 1227 \| Robinia pseudoacacia \| HM049518.1 \| HM850308.1 \| AN \|  \| Inv \| \| 1228 \| Romulea columnae \| HM850494.1 \| HM850310.1 \| N \|  \| Nat \| \| 1229 \| Rorippa amphibia \| AF174530.1 \| AF020327.1 \| N \|  \| Nat \| \| 1230 \| Rorippa islandica \| DQ406770.1 \| JN892055.1 \| N \|  \| Nat \| \| 1231 \| Rorippa microphylla \| JN896047.1 \| HQ590243.1 \| N \|  \| Nat \| \| 1232 \| Rorippa nasturtium-aquaticum \| AP009376.1 \| AP009376.1 \| N \|  \| Nat \| \| 1233 \| Rorippa palustris \| AF144355.1 \| JN965821.1 \| N \|  \| Nat \| \| 1234 \| Rorippa sylvestris \| JN894865.1 \| AF020328.1 \| N \|  \| Nat \| \| 1235 \| Rosa agrestis \| JN894864.1 \| JN891915.1 \| N \|  \| Nat \| \| 1236 \| Rosa arvensis \| AB048598.1 \| JN891164.1 \| N \|  \| Nat \| \| 1237 \| Rosa caesia \| JN895601.1 \| JN892951.1 \| N \|  \| Nat \| \| 1238 \| Rosa canina agg. \| AB011980.1 \| FN689381.1 \| N \|  \| Nat \| \| 1239 \| Rosa micrantha \| JN894863.1 \| HE963634.1 \| N \|  \| Nat \| \| 1240 \| Rosa mollis \| JN894862.1 \| JN892943.1 \| N \|  \| Nat \| \| 1241 \| Rosa obtusifolia \| JN895795.1 \| JN893206.1 \| N \|  \| Nat \| \| 1242 \| Rosa pimpinellifolia \| AB011976.1 \| JN893205.1 \| N \|  \| Nat \| \| 1243 \| Rosa rubiginosa \| AB011981.1 \| JN893209.1 \| N \|  \| Nat \| \| 1244 \| Rosa rugosa \| AB039296.1 \| T.S. \| AN \| 1 \| Inv \| \| 1245 \| Rosa sherardii \| JN894317.1 \| JN893202.1 \| N \|  \| Nat \| \| 1246 \| Rosa stylosa \| JN894318.1 \| JN890852.1 \| N \|  \| Nat \| \| 1247 \| Rosa tomentosa \| JN894315.1 \| JN890846.1 \| N \|  \| Nat \| \| 1248 \| Rubia peregrina \| JN894316.1 \| JN892897.1 \| N \|  \| Nat \| \| 1249 \| Rubus caesius \| JN895641.1 \| JN890848.1 \| N \|  \| Nat \| \| 1250 \| Rubus chamaemorus \| AY366358.1 \| JN965827.1 \| N \|  \| Nat \| \| 1251 \| Rubus fruticosus agg. \| JN566091.1 \| JN893026.1 \| N \|  \| Nat \| \| 1252 \| Rubus idaeus \| JN894962.1 \| U06825.1 \| N \|  \| Nat \| \| 1253 \| Rubus saxatilis \| \| JN891152.1 \| N \|  \| Nat \| \| 1254 \| Rubus spectabilis \| \| T.S. \| AN \|  \| Non-inv \| \| 1255 \| Rumex acetosa \| JN895940.1 \| AY395559.1 \| N \|  \| Nat \| \| 1256 \| Rumex acetosella \| JN893966.1 \| HM850316.1 \| N \|  \| Nat \| \| 1257 \| Rumex conglomeratus \| JN895741.1 \| HM850319.1 \| N \|  \| Nat \| \| 1258 \| Rumex crispus \| EU840458.1 \| HM850320.1 \| N \|  \| Nat \| \| 1259 \| Rumex hydrolapathum \| JN895278.1 \| JN893122.1 \| N \|  \| Nat \| \| 1260 \| Rumex longifolius \| \| T.S. \| N \|  \| Nat \| \| 1261 \| Rumex maritimus \| JN894596.1 \| JN892880.1 \| N \|  \| Nat \| \| 1262 \| Rumex obtusifolius \| JN895557.1 \| HM850322.1 \| N \|  \| Nat \| \| 1263 \| Rumex palustris \| JN895664.1 \| JN892877.1 \| N \|  \| Nat \| \| 1264 \| Rumex pulcher \| HM851087.1 \| HM850323.1 \| N \|  \| Nat \| \| 1265 \| Rumex rupestris \| JN894961.1 \| JN892044.1 \| N \|  \| Nat \| \| 1266 \| Rumex sanguineus \| FJ395432.1 \| JN892658.1 \| N \|  \| Nat \| \| 1267 \| Ruppia cirrhosa \| AB728682.1 \| JN113275.1 \| N \|  \| Nat \| \| 1268 \| Ruppia maritima \| JN893851.1 \| HM850324.1 \| N \|  \| Nat \| \| 1269 \| Ruscus aculeatus \| JQ276421.1 \| JQ273926.1 \| N \|  \| Nat \| \| 1270 \| Sagina apetala \| HM850780.1 \| HM850327.1 \| N \|  \| Nat \| \| 1271 \| Sagina maritima \| JN895769.1 \| HM850328.1 \| N \|  \| Nat \| \| 1272 \| Sagina nivalis \| \| T.S. \| N \|  \| Nat \| \| 1273 \| Sagina nodosa \| JN895768.1 \| JN890856.1 \| N \|  \| Nat \| \| 1274 \| Sagina procumbens \| JN895587.1 \| HM850329.1 \| N \|  \| Nat \| \| 1275 \| Sagina saginoides \| \| T.S. \| N \|  \| Nat \| \| 1276 \| Sagina subulata \| JN895586.1 \| JN892923.1 \| N \|  \| Nat \| \| 1277 \| Sagittaria sagittifolia \| JN894086.1 \| GU344672.1 \| N \|  \| Nat \| \| 1278 \| Salicornia dolichostachya \| DQ468650.1 \| AY270125.1 \| N \|  \| Nat \| \| 1279 \| Salicornia europaea \| JN894093.1 \| HM131777.1 \| N \|  \| Nat \| \| 1280 \| Salicornia fragilis \| AY996312.1 \| JN890858.1 \| N \|  \| Nat \| \| 1281 \| Salicornia nitens \| JN895421.1 \| JN892676.1 \| N \|  \| Nat \| \| 1282 \| Salicornia obscura \| DQ468651.1 \|  \| N \|  \| Nat \| \| 1283 \| Salicornia pusilla \| JN894092.1 \| JN893164.1 \| N \|  \| Nat \| \| 1284 \| Salicornia ramosissima \| AY996317.1 \| JN890862.1 \| N \|  \| Nat \| \| 1285 \| Salix alba \| EU790677.1 \| AB012780.1 \| AR \|  \| Inv \| \| 1286 \| Salix arbuscula \| T.S. \| T.S. \| N \|  \| Nat \| \| 1287 \| Salix aurita \| JN893910.1 \| JN892174.1 \| N \|  \| Nat \| \| 1288 \| Salix caprea \| JN893911.1 \| FR694871.1 \| N \|  \| Nat \| \| 1289 \| Salix cinerea \| JN895010.1 \| JN893030.1 \| N \|  \| Nat \| \| 1290 \| Salix fragilis \| JN894892.1 \| AJ418841.1 \| AR \|  \| Inv \| \| 1291 \| Salix herbacea \| EU790671.1 \| JN891952.1 \| N \|  \| Nat \| \| 1292 \| Salix lanata \| JN966591.1 \| JN965911.1 \| N \|  \| Nat \| \| 1293 \| Salix lapponum \| \| GU373339.1 \| N \|  \| Nat \| \| 1294 \| Salix myrsinifolia \| GU373362.1 \| GU373340.1 \| N \|  \| Nat \| \| 1295 \| Salix myrsinites \| GU373368.1 \| T.S. \| N \|  \| Nat \| \| 1296 \| Salix pentandra \| EU790685.1 \| AB012791.1 \| N \|  \| Nat \| \| 1297 \| Salix phylicifolia \| \| GU373349.1 \| N \|  \| Nat \| \| 1298 \| Salix purpurea \| JN894735.1 \| JN891726.1 \| N \|  \| Nat \| \| 1299 \| Salix repens \| JN893909.1 \| JN891724.1 \| N \|  \| Nat \| \| 1300 \| Salix reticulata \| EF135592.1 \| AJ235793.1 \| N \|  \| Nat \| \| 1301 \| Salix triandra \| EU790687.1 \| FJ788587.1 \| AR \|  \| Non-inv \| \| 1302 \| Salix viminalis \| JN893913.1 \| HE610663 \| AR \|  \| Non-inv \| \| 1303 \| Salsola kali \| AY514843.1 \| HM850332.1 \| N \|  \| Nat \| \| 1304 \| Salvia verbenaca \| JN896169.1 \| HM850333.1 \| N \|  \| Nat \| \| 1305 \| Sambucus ebulus \| JN896168.1 \| JN893720.1 \| AR \|  \| Non-inv \| \| 1306 \| Sambucus nigra \| AY310458.1 \| HM850334.1 \| N \|  \| Nat \| \| 1307 \| Sambucus racemosa \| FN668835.1 \| AF446928.1 \| AN \|  \| Non-inv \| \| 1308 \| Samolus valerandi \| JN895196.1 \| HM850335.1 \| N \|  \| Nat \| \| 1309 \| Sanguisorba minor \| AB073694.1 \| HM850336.1 \| N \|  \| Nat \| \| 1310 \| Sanguisorba officinalis \| AB073696.1 \| AY395560.1 \| N \|  \| Nat \| \| 1311 \| Sanicula europaea \| JN894982.1 \| DQ133820.1 \| N \|  \| Nat \| \| 1312 \| Saponaria officinalis \| HM850784.1 \| HM850338.1 \| AR \|  \| Non-inv \| \| 1313 \| Sarcocornia perennis \| AY996302.1 \| JQ412418.1 \| N \|  \| Nat \| \| 1314 \| Saussurea alpina \| \| T.S. \| N \|  \| Nat \| \| 1315 \| Saxifraga aizoides \| \| JN965982.1 \| N \|  \| Nat \| \| 1316 \| Saxifraga cernua \| L34140.1 \| U06215.1 \| N \|  \| Nat \| \| 1317 \| Saxifraga cespitosa \| JN966661.1 \| JN965985.1 \| N \|  \| Nat \| \| 1318 \| Saxifraga granulata \| \| JN892372.1 \| N \|  \| Nat \| \| 1319 \| Saxifraga hirsuta \| \| T.S. \| N \|  \| Nat \| \| 1320 \| Saxifraga hypnoides \| JN894659.1 \| JN891619.1 \| N \|  \| Nat \| \| 1321 \| Saxifraga nivalis \| JN895150 \| JN892315 \| N \|  \| Nat \| \| 1322 \| Saxifraga oppositifolia \| L34143.1 \| U06217.1 \| N \|  \| Nat \| \| 1323 \| Saxifraga rivularis \| \| T.S. \| N \|  \| Nat \| \| 1324 \| Saxifraga spathularis \| \| T.S. \| N \|  \| Nat \| \| 1325 \| Saxifraga stellaris \| AF115493.1 \| KC749991.1 \| N \|  \| Nat \| \| 1326 \| Saxifraga tridactylites \| JN894658.1 \| JN892110.1 \| N \|  \| Nat \| \| 1327 \| Scabiosa columbaria \| JN894429.1 \| AF446948.1 \| N \|  \| Nat \| \| 1328 \| Scandix pecten-veneris \| U58578.1 \| JN892790.1 \| AR \|  \| Non-inv \| \| 1329 \| Scheuchzeria palustris \| GQ452338.1 \| U03728.1 \| N \|  \| Nat \| \| 1330 \| Schoenoplectus lacustris \| JN895489.1 \| AM999865.1 \| N \|  \| Nat \| \| 1331 \| Schoenoplectus tabernaemontani \| JN895494.1 \| GQ130365.1 \| N \|  \| Nat \| \| 1332 \| Schoenoplectus triqueter \| \| GQ130367.1 \| N \|  \| Nat \| \| 1333 \| Schoenus nigricans \| JN896217.1 \| AM999870.1 \| N \|  \| Nat \| \| 1334 \| Scilla autumnalis \| T.S. \| JN893435.1 \| N \|  \| Nat \| \| 1335 \| Scilla verna \|  \| JN893558.1 \| N \|  \| Nat \| \| 1336 \| Scirpoides holoschoenus \| HE967485.1 \| AM999871.1 \| N \|  \| Nat \| \| 1337 \| Scirpus sylvaticus \| JN895496.1 \| AM999875.1 \| N \|  \| Nat \| \| 1338 \| Scleranthus annuus \| JN895583.1 \| AY270145.1 \| N \|  \| Nat \| \| 1339 \| Scleranthus perennis \| AY514847.1 \| JN892918.1 \| N \|  \| Nat \| \| 1340 \| Scrophularia auriculata \| JN895492.1 \| HM850343.1 \| N \|  \| Nat \| \| 1341 \| Scrophularia nodosa \| JN896243.1 \| JN892046.1 \| N \|  \| Nat \| \| 1342 \| Scrophularia scorodonia \| HM850963.1 \| HM850344.1 \| AN \|  \| Non-inv \| \| 1343 \| Scrophularia vernalis \| \| T.S. \| AN \|  \| Non-inv \| \| 1344 \| Scutellaria galericulata \| JN895418.1 \| JN892670.1 \| N \|  \| Nat \| \| 1345 \| Scutellaria minor \| JN895419.1 \| HM850345.1 \| N \|  \| Nat \| \| 1346 \| Sedum acre \|  \| HQ590268.1 \| N \|  \| Nat \| \| 1347 \| Sedum album \| JN895776.1 \| JN891488.1 \| AR \|  \| Non-inv \| \| 1348 \| Sedum anglicum \| \| JN891838.1 \| N \|  \| Nat \| \| 1349 \| Sedum forsterianum \| \| JN892578.1 \| N \|  \| Nat \| \| 1350 \| Sedum rosea \| JN895351.1 \| JN891610.1 \| N \|  \| Nat \| \| 1351 \| Sedum rupestre \| AF115667.1 \| HQ590269.1 \| AN \|  \| Non-inv \| \| 1352 \| Sedum telephium \| JN895352.1 \| HQ590138.1 \| N \|  \| Nat \| \| 1353 \| Selaginella selaginoides \| \| AF419048.1 \| N \|  \| Nat \| \| 1354 \| Sempervivum tectorum \| AF115671.1 \| T.S. \| AN \|  \| Non-inv \| \| 1355 \| Senecio aquaticus \| JN894078.1 \| HM850080.1 \| N \|  \| Nat \| \| 1356 \| Senecio cambrensis \| JN894727.1 \| JN891718.1 \| NE \|  \| Nat \| \| 1357 \| Senecio cineraria \| AF460012.1 \| HM850081.1 \| AN \|  \| Non-inv \| \| 1358 \| Senecio erucifolius \| JN895354.1 \| JN892024.1 \| N \|  \| Nat \| \| 1359 \| Senecio jacobaea \| JN895164.1 \| GU817769.1 \| N \|  \| Nat \| \| 1360 \| Senecio paludosus \| JN895164.1 \|  \| N \|  \| Nat \| \| 1361 \| Senecio squalidus \| JN789637.1 \|  \| AN \|  \| Inv \| \| 1362 \| Senecio sylvaticus \| JN895353.1 \| HM850346.1 \| N \|  \| Nat \| \| 1363 \| Senecio viscosus \| AF459996.2 \|  \| AN \|  \| Inv \| \| 1364 \| Senecio vulgaris \| AF151509.1 \| JN892571 \| N \|  \| Nat \| \| 1365 \| Seriphidium maritimum \| \| T.S. \| N \|  \| Nat \| \| 1366 \| Serratula tinctoria \| JN894180.1 \| JN893275.1 \| N \|  \| Nat \| \| 1367 \| Seseli libanotis \| \| T.S. \| N \|  \| Nat \| \| 1368 \| Sesleria caerulea \| DQ786942.1 \| EF125156.1 \| N \|  \| Nat \| \| 1369 \| Setaria viridis \| JX520944.1 \| KC164339.1 \| AN \|  \| Non-inv \| \| 1370 \| Sherardia arvensis \| JN894494.1 \| HM850351.1 \| N \|  \| Nat \| \| 1371 \| Sibbaldia procumbens \| \| T.S. \| N \|  \| Nat \| \| 1372 \| Sibthorpia europaea \| JN896239.1 \| JN893789 \| N \|  \| Nat \| \| 1373 \| Silaum silaus \| JN894498.1 \| JN892713 \| N \|  \| Nat \| \| 1374 \| Silene acaulis \| EF547235.1 \| JN891509 \| N \|  \| Nat \| \| 1375 \| Silene conica \| NC_016729.1 \| NC_016729.1 \| N \|  \| Nat \| \| 1376 \| Silene dioica \| EF646912.1 \| EF646927.1 \| N \|  \| Nat \| \| 1377 \| Silene gallica \| FJ589528.1 \| HM850354.1 \| AR \|  \| Non-inv \| \| 1378 \| Silene latifolia \| EF647048.1 \| EF647098.1 \| AR \|  \| Non-inv \| \| 1379 \| Silene noctiflora \| NC_016728.1 \| NC_016728.1 \| AR \|  \| Non-inv \| \| 1380 \| Silene nutans \| EF547241.1 \| JN891737.1 \| N \|  \| Nat \| \| 1381 \| Silene otites \| FJ589547.1 \| T.S. \| N \|  \| Nat \| \| 1382 \| Silene uniflora \| FJ589565.1 \| HM850355.1 \| N \|  \| Nat \| \| 1383 \| Silene vulgaris \| NC_016727.1 \| NC_016727.1 \| N \|  \| Nat \| \| 1384 \| Silybum marianum \| AY013551.1 \| HM850356.1 \| AR \|  \| Non-inv \| \| 1385 \| Sinapis alba \| JF926674.1 \| HM849823.1 \| AR \|  \| Non-inv \| \| 1386 \| Sinapis arvensis \| JN895631.1 \| HQ590272.1 \| AR \|  \| Inv \| \| 1387 \| Sison amomum \| JN895633.1 \| JQ933482.1 \| N \|  \| Nat \| \| 1388 \| Sisymbrium altissimum \| JN585004.1 \| JN847834.1 \| AN \|  \| Non-inv \| \| 1389 \| Sisymbrium officinale \| JN895634.1 \| HM850358.1 \| AR \|  \| Inv \| \| 1390 \| Sisymbrium orientale \| \| T.S. \| AN \|  \| Inv \| \| 1391 \| Sisyrinchium bermudiana \| JN565618.1 \|  \| N \|  \| Nat \| \| 1392 \| Sium latifolium \| \| T.S. \| N \|  \| Nat \| \| 1393 \| Smyrnium olusatrum \| JN895635.1 \| JN892715.1 \| AR \|  \| Inv \| \| 1394 \| Solanum dulcamara \| JN894601.1 \| HM850363.1 \| N \|  \| Nat \| \| 1395 \| Solanum sarrachoides \| T.S. \| T.S. \| AN \|  \| Non-inv \| \| 1396 \| Solanum tuberosum \| NC_008096.2 \| NC_008096.2 \| AN \|  \| Non-inv \| \| 1397 \| Soleirolia soleirolii \| \| T.S. \| AN \|  \| Inv \| \| 1398 \| Solidago canadensis \| EU749415.1 \| EU677022.1 \| AN \|  \| Inv \| \| 1399 \| Solidago gigantea \| HQ593451.1 \| HM850369.1 \| AN \|  \| Non-inv \| \| 1400 \| Solidago virgaurea \| JN895829.1 \| JN891242.1 \| N \|  \| Nat \| \| 1401 \| Sonchus arvensis \| DQ840453.1 \| JN891244.1 \| N \|  \| Nat \| \| 1402 \| Sonchus asper \| DQ840454.1 \| HM850372.1 \| N \|  \| Nat \| \| 1403 \| Sonchus oleraceus \| DQ840449.1 \| EU385018.1 \| N \|  \| Nat \| \| 1404 \| Sonchus palustris \| DQ022984.1 \| T.S. \| N \|  \| Nat \| \| 1405 \| Sorbus anglica \| JN894457.1 \| JN891905.1 \| NE \|  \| Nat \| \| 1406 \| Sorbus aria \| JN895195.1 \| JN891344.1 \| N \|  \| Nat \| \| 1407 \| Sorbus arranensis \| \| T.S. \| NE \|  \| Nat \| \| 1408 \| Sorbus aucuparia \| JN896162.1 \| HQ590284.1 \| N \|  \| Nat \| \| 1409 \| Sorbus bristoliensis \| \| T.S. \| NE \|  \| Nat \| \| 1410 \| Sorbus devoniensis \| \| T.S. \| NE \|  \| Nat \| \| 1411 \| Sorbus domestica \| JN895827.1 \| JN893246.1 \| N \|  \| Nat \| \| 1412 \| Sorbus eminens \| JN895324.1 \| JN891098.1 \| NE \|  \| Nat \| \| 1413 \| Sorbus hibernica \| JN895675.1 \| JN891820.1 \| NE \|  \| Nat \| \| 1414 \| Sorbus intermedia \| \| T.S. \| AN \|  \| Non-inv \| \| 1415 \| Sorbus lancastriensis \| \| T.S. \| NE \|  \| Nat \| \| 1416 \| Sorbus leptophylla \| JN895826.1 \| JN893528.1 \| NE \|  \| Nat \| \| 1417 \| Sorbus leyana \| JN895263.1 \| JN893286.1 \| NE \|  \| Nat \| \| 1418 \| Sorbus minima \| JN895261.1 \| JN891827.1 \| NE \|  \| Nat \| \| 1419 \| Sorbus porrigentiformis \| JN895825.1 \| JN891598.1 \| NE \|  \| Nat \| \| 1420 \| Sorbus pseudofennica \| \| T.S. \| NE \|  \| Nat \| \| 1421 \| Sorbus rupicola \| JN894653.1 \| JN891596.1 \| N \|  \| Nat \| \| 1422 \| Sorbus subcuneata \| \| T.S. \| NE \|  \| Nat \| \| 1423 \| Sorbus torminalis \| JN895323.1 \| JN891605.1 \| N \|  \| Nat \| \| 1424 \| Sorbus vexans \| \| T.S. \| NE \|  \| Nat \| \| 1425 \| Sparganium angustifolium \| JN895154.1 \| JN893590.1 \| N \|  \| Nat \| \| 1426 \| Sparganium emersum \| HQ593456.1 \| GU344676.1 \| N \|  \| Nat \| \| 1427 \| Sparganium erectum \| JQ435570.1 \| JN893201.1 \| N \|  \| Nat \| \| 1428 \| Sparganium natans \| JN894174.1 \| JN966012.1 \| N \|  \| Nat \| \| 1429 \| Spartina anglica \| HE573972.1 \| AM849382.1 \| NE \|  \| Nat \| \| 1430 \| Spergula arvensis \| JN894814.1 \| HM850378.1 \| N \|  \| Nat \| \| 1431 \| Spergularia bocconei \| HM850776.1 \| HM850380.1 \| AN \|  \| Non-inv \| \| 1432 \| Spergularia marina \| JN895293.1 \| HM850381.1 \| N \|  \| Nat \| \| 1433 \| Spergularia media \| JN894902.1 \| JN891830.1 \| N \|  \| Nat \| \| 1434 \| Spergularia rubra \| JN895148.1 \| JN893434.1 \| N \|  \| Nat \| \| 1435 \| Spergularia rupicola \| JN894504.1 \| JN892196.1 \| N \|  \| Nat \| \| 1436 \| Spiranthes romanzoffiana \| T.S. \| FJ571354.1 \| N \|  \| Nat \| \| 1437 \| Spiranthes spiralis \| JN894278.1 \| FJ571355.1 \| N \|  \| Nat \| \| 1438 \| Spirodela polyrhiza \| NC_015891.1 \| NC_015891.1 \| N \|  \| Nat \| \| 1439 \| Stachys alpina \| JN894809.1 \| JN892674.1 \| AN \|  \| Non-inv \| \| 1440 \| Stachys arvensis \| JN895212.1 \| HM850384.1 \| AR \|  \| Non-inv \| \| 1441 \| Stachys officinalis \| JN895211.1 \| AF502015.1 \| N \|  \| Nat \| \| 1442 \| Stachys palustris \| JN895214.1 \| JN891835.1 \| N \|  \| Nat \| \| 1443 \| Stachys sylvatica \| FJ395437.1 \| AF502022.1 \| N \|  \| Nat \| \| 1444 \| Stellaria graminea \| JN893905.1 \| FJ395572.1 \| N \|  \| Nat \| \| 1445 \| Stellaria holostea \| JN894785.1 \| FJ395575.1 \| N \|  \| Nat \| \| 1446 \| Stellaria media \| JQ844147.1 \| M62570.1 \| N \|  \| Nat \| \| 1447 \| Stellaria neglecta \| JN893860.1 \| JN892188.1 \| N \|  \| Nat \| \| 1448 \| Stellaria nemorum \| AY936298.1 \| JN893484.1 \| N \|  \| Nat \| \| 1449 \| Stellaria pallida \| JN893858.1 \| JN890562.1 \| N \|  \| Nat \| \| 1450 \| Stellaria palustris \| \| JN890558.1 \| N \|  \| Nat \| \| 1451 \| Stellaria uliginosa \| HM850778.1 \| HM850385.1 \| N \|  \| Nat \| \| 1452 \| Suaeda maritima \| DQ468647.1 \| AY270137.1 \| N \|  \| Nat \| \| 1453 \| Suaeda vera \| AY042658.1 \| JN893487.1 \| N \|  \| Nat \| \| 1454 \| Subularia aquatica \| \| T.S. \| N \|  \| Nat \| \| 1455 \| Succisa pratensis \| JN896004.1 \| JN893486.1 \| N \|  \| Nat \| \| 1456 \| Symphoricarpos albus \| AY310459.1 \| L11682.1 \| AN \|  \| Inv \| \| 1457 \| Symphytum officinale \| JN896115.1 \| JN893637.1 \| N \|  \| Nat \| \| 1458 \| Symphytum orientale \| \| T.S. \| AN \|  \| Non-inv \| \| 1459 \| Syringa vulgaris \| JN591007.1 \| DQ673303.1 \| AN \|  \| Non-inv \| \| 1460 \| Tamus communis \| JN895566.1 \| AF307474.1 \| N \|  \| Nat \| \| 1461 \| Tanacetum parthenium \| JN895338.1 \| HM850390.1 \| AR \|  \| Non-inv \| \| 1462 \| Tanacetum vulgare \| JN894898.1 \| JN891960.1 \| N \|  \| Nat \| \| 1463 \| Taraxacum \| HM850656.1 \| HM850393.1 \| N \|  \| Nat \| \| 1464 \| Taxus baccata \| JN895853 \| JN891269 \| N \|  \| Nat \| \| 1465 \| Teesdalia nudicaulis \| GQ424601.1 \| HE616650.1 \| N \|  \| Nat \| \| 1466 \| Tellima grandiflora \| L34149.1 \| U06222.1 \| AN \|  \| Non-inv \| \| 1467 \| Teucrium chamaedrys \| FR865047.1 \| FR865125.1 \| AN \|  \| Non-inv \| \| 1468 \| Teucrium scordium \| JN895070.1 \| JN893048.1 \| N \|  \| Nat \| \| 1469 \| Teucrium scorodonia \| JN896167.1 \| HM850397.1 \| N \|  \| Nat \| \| 1470 \| Thalictrum alpinum \| JN895143.1 \| JX258332.1 \| N \|  \| Nat \| \| 1471 \| Thalictrum flavum \| JN895149.1 \| JN892312.1 \| N \|  \| Nat \| \| 1472 \| Thalictrum minus \| JN895477.1 \| JN893472.1 \| N \|  \| Nat \| \| 1473 \| Thelypteris palustris \| JF832292.1 \| U05947.1 \| N \|  \| Nat \| \| 1474 \| Thesium humifusum \| JN894197.1 \| JN892108.1 \| N \|  \| Nat \| \| 1475 \| Thlaspi arvense \| AF144360.1 \| JQ933501.1 \| AR \|  \| Non-inv \| \| 1476 \| Thlaspi caerulescens \| JN893942.1 \| FN594826.1 \| N \|  \| Nat \| \| 1477 \| Thlaspi perfoliatum \| AF144362.1 \|  \| N \|  \| Nat \| \| 1478 \| Thuja plicata \| AF152216.1 \| AF127428.2 \| AN \|  \| Non-inv \| \| 1479 \| Thymus polytrichus \| JN893961.1 \| JN892956.1 \| N \|  \| Nat \| \| 1480 \| Thymus pulegioides \| JN894907.1 \| JN892333.1 \| N \|  \| Nat \| \| 1481 \| Thymus serpyllum \| AY840173.1 \| T.S. \| N \|  \| Nat \| \| 1482 \| Tilia cordata \| JN894340.1 \| JN893524.1 \| N \|  \| Nat \| \| 1483 \| Tilia platyphyllos \| JN894338.1 \| JN890677.1 \| N \|  \| Nat \| \| 1484 \| Tofieldia pusilla \| AB451566.1 \| AJ286562.1 \| N \|  \| Nat \| \| 1485 \| Tolmiea menziesii \| AB161146.1 \| U06223.1 \| AN \|  \| Non-inv \| \| 1486 \| Tordylium maximum \| \| T.S. \| AN \|  \| Non-inv \| \| 1487 \| Torilis arvensis \| JN895444.1 \| HM850403.1 \| AR \|  \| Non-inv \| \| 1488 \| Torilis japonica \| JN895299.1 \| FJ395562.1 \| N \|  \| Nat \| \| 1489 \| Torilis nodosa \| JN893952.1 \| HM850404.1 \| N \|  \| Nat \| \| 1490 \| Tragopogon pratensis \| JN895071.1 \| AY395563.1 \| N \|  \| Nat \| \| 1491 \| Trichomanes speciosum \| T.S. \| T.S. \| N \|  \| Nat \| \| 1492 \| Trichophorum cespitosum \| JN893954.1 \| AJ811003.1 \| N \|  \| Nat \| \| 1493 \| Trientalis europaea \| \| U96655.1 \| N \|  \| Nat \| \| 1494 \| Trifolium arvense \| JN894251.1 \| HM850409.1 \| N \|  \| Nat \| \| 1495 \| Trifolium bocconei \| \| T.S. \| N \|  \| Nat \| \| 1496 \| Trifolium campestre \| JN894254.1 \| JX505488.1 \| N \|  \| Nat \| \| 1497 \| Trifolium dubium \| AF522121.1 \| HM850412.1 \| N \|  \| Nat \| \| 1498 \| Trifolium fragiferum \| AF522122.1 \| HM850413.1 \| N \|  \| Nat \| \| 1499 \| Trifolium glomeratum \| JX505831.1 \| HM850414.1 \| N \|  \| Nat \| \| 1500 \| Trifolium hybridum \| AF522125.1 \| T.S. \| AN \|  \| Non-inv \| \| 1501 \| Trifolium incarnatum \| AF522126.1 \| HM850415.1 \| N \|  \| Nat \| \| 1502 \| Trifolium medium \| JN894074.1 \| JN890836.1 \| N \|  \| Nat \| \| 1503 \| Trifolium micranthum \| JN894443.1 \| JN891323.1 \| N \|  \| Nat \| \| 1504 \| Trifolium ornithopodioides \| JN894445.1 \| HM850418.1 \| N \|  \| Nat \| \| 1505 \| Trifolium pratense \| JN894441.1 \| HM850419.1 \| N \|  \| Nat \| \| 1506 \| Trifolium repens \| AF522131.1 \| HM850420.1 \| N \|  \| Nat \| \| 1507 \| Trifolium scabrum \| JN895610.1 \| HM850422.1 \| N \|  \| Nat \| \| 1508 \| Trifolium squamosum \| JN895608.1 \| HM850423.1 \| N \|  \| Nat \| \| 1509 \| Trifolium striatum \| AF522134.1 \| HM850425.1 \| N \|  \| Nat \| \| 1510 \| Trifolium strictum \| JN895605.1 \| JN893214.1 \| N \|  \| Nat \| \| 1511 \| Trifolium subterraneum \| NC_011828.1 \| NC_011828.1 \| N \|  \| Nat \| \| 1512 \| Trifolium suffocatum \| JN895800.1 \| HM850427.1 \| N \|  \| Nat \| \| 1513 \| Triglochin maritimum \| AB088782.1 \| GQ452333.1 \| N \|  \| Nat \| \| 1514 \| Triglochin palustris \| GQ452340.1 \| GQ452334.1 \| N \|  \| Nat \| \| 1515 \| Trinia glauca \| T.S. \| T.S. \| N \|  \| Nat \| \| 1516 \| Tripleurospermum maritimum \| JN895475.1 \| JN892563.1 \| N \|  \| Nat \| \| 1517 \| Trisetum flavescens \| JN895838.1 \| AY395565.1 \| N \|  \| Nat \| \| 1518 \| Trollius europaeus \| AY515236.1 \| JN893474.1 \| N \|  \| Nat \| \| 1519 \| Tsuga heterophylla \| EF395598.1 \| X63659.1 \| AN \|  \| Non-inv \| \| 1520 \| Tuberaria guttata \| DQ092971.1 \| FJ225853.1 \| N \|  \| Nat \| \| 1521 \| Tussilago farfara \| JN896028.1 \| JN893522.1 \| N \|  \| Nat \| \| 1522 \| Typha angustifolia \| AY952419.1 \| AY952434.1 \| N \|  \| Nat \| \| 1523 \| Typha latifolia \| NC_013823.1 \| NC_013823.1 \| N \|  \| Nat \| \| 1524 \| Ulex europaeus \| JQ669586.1 \| HM850431.1 \| N \|  \| Nat \| \| 1525 \| Ulex gallii \| JN895798.1 \| JN893750.1 \| N \|  \| Nat \| \| 1526 \| Ulex minor \| HM851133.1 \| HM850432.1 \| N \|  \| Nat \| \| 1527 \| Ulmus glabra \| JN896191.1 \| JN893234.1 \| N \|  \| Nat \| \| 1528 \| Ulmus minor \| JN895764.1 \| JN893087.1 \| N \|  \| Nat \| \| 1529 \| Umbilicus rupestris \| JN895756.1 \| JN892754.1 \| N \|  \| Nat \| \| 1530 \| Urtica dioica \| GU266610.1 \| AF500361.1 \| N \|  \| Nat \| \| 1531 \| Urtica urens \| EU002192.1 \| HM850437.1 \| AR \|  \| Non-inv \| \| 1532 \| Utricularia australis \| AF531823.1 \| JN893264.1 \| N \|  \| Nat \| \| 1533 \| Utricularia intermedia sens.lat. \| AF531839.1 \| JN890779.1 \| N \|  \| Nat \| \| 1534 \| Utricularia minor \| JN894028.1 \| JN890777.1 \| N \|  \| Nat \| \| 1535 \| Utricularia stygia \| JN894027.1 \| JN890776.1 \| N \|  \| Nat \| \| 1536 \| Utricularia vulgaris sens.str. \| AF531831.1 \| JN890555.1 \| N \|  \| Nat \| \| 1537 \| Vaccinium microcarpum \| JN966392.1 \| JN965697.1 \| N \|  \| Nat \| \| 1538 \| Vaccinium myrtillus \| AF382810.1 \| AY145447.1 \| N \|  \| Nat \| \| 1539 \| Vaccinium oxycoccos \| JN895357.1 \| JN893514.1 \| N \|  \| Nat \| \| 1540 \| Vaccinium uliginosum \| AF419717.1 \| AF421107.1 \| N \|  \| Nat \| \| 1541 \| Vaccinium vitis-idaea \| AF382819.1 \| AF419837.1 \| N \|  \| Nat \| \| 1542 \| Valeriana dioica \| AY362531.1 \| AY362489.1 \| N \|  \| Nat \| \| 1543 \| Valeriana officinalis \| AY310467.1 \| AY362490.1 \| N \|  \| Nat \| \| 1544 \| Valeriana pyrenaica \| AY310491.1 \| AF421107.1 \| AN \|  \| Non-inv \| \| 1545 \| Valerianella carinata \| JN895708.1 \| JN892721.1 \| AR \|  \| Non-inv \| \| 1546 \| Valerianella dentata \| JN896023.1 \| JN892733.1 \| AR \|  \| Non-inv \| \| 1547 \| Valerianella eriocarpa \| AY310466.1 \| JN892850.1 \| AN \|  \| Non-inv \| \| 1548 \| Valerianella locusta \| AY310465.1 \| AF446954.1 \| N \|  \| Nat \| \| 1549 \| Valerianella rimosa \| GU188969.1 \| JN892853.1 \| AR \|  \| Non-inv \| \| 1550 \| Verbascum lychnitis \| \| T.S. \| N \|  \| Nat \| \| 1551 \| Verbascum nigrum \| JN896238.1 \| JN893706.1 \| N \|  \| Nat \| \| 1552 \| Verbascum thapsus \| HQ593484.1 \| L36452.1 \| N \|  \| Nat \| \| 1553 \| Verbascum virgatum \| HM850966.1 \| HM850442.1 \| AN \|  \| Non-inv \| \| 1554 \| Verbena officinalis \| JN894906.1 \| HM850444.1 \| AR \|  \| Non-inv \| \| 1555 \| Veronica agrestis \| JN893993.1 \| HM850446.1 \| AR \|  \| Non-inv \| \| 1556 \| Veronica alpina \| \| T.S. \| N \|  \| Nat \| \| 1557 \| Veronica anagallis-aquatica \| JN894700.1 \| AY034021.1 \| N \|  \| Nat \| \| 1558 \| Veronica arvensis \| AF052003.1 \| HM850447.1 \| N \|  \| Nat \| \| 1559 \| Veronica beccabunga \| JN894414.1 \| JN891292.1 \| N \|  \| Nat \| \| 1560 \| Veronica catenata \| JN894415.1 \| HM850448.1 \| N \|  \| Nat \| \| 1561 \| Veronica chamaedrys \| FJ395446.1 \| FJ395581.1 \| N \|  \| Nat \| \| 1562 \| Veronica filiformis \| \| FM207429.1 \| AN \|  \| Inv \| \| 1563 \| Veronica fruticans \| \| T.S. \| N \|  \| Nat \| \| 1564 \| Veronica hederifolia \| JN894703.1 \| JN891678.1 \| AR \|  \| Inv \| \| 1565 \| Veronica montana \| JN894194.1 \| JN891296.1 \| N \|  \| Nat \| \| 1566 \| Veronica officinalis \| JN895208.1 \| HM850450.1 \| N \|  \| Nat \| \| 1567 \| Veronica persica \| HQ384536.1 \| HM850452.1 \| AN \|  \| Inv \| \| 1568 \| Veronica polita \| \| HM850453.1 \| AN \|  \| Non-inv \| \| 1569 \| Veronica scutellata \| JN894218.1 \| JN891680.1 \| N \|  \| Nat \| \| 1570 \| Veronica serpyllifolia \| HM851040.1 \| HM850454.1 \| N \|  \| Nat \| \| 1571 \| Veronica spicata \| JN894225.1 \| JN892244.1 \| N \|  \| Nat \| \| 1572 \| Veronica verna \| \| T.S. \| N \|  \| Nat \| \| 1573 \| Viburnum lantana \| JN895394.1 \| HQ591736.1 \| N \|  \| Nat \| \| 1574 \| Viburnum opulus \| JN896159.1 \| HQ591752.1 \| N \|  \| Nat \| \| 1575 \| Vicia bithynica \| JN895988.1 \| HM850458.1 \| N \|  \| Nat \| \| 1576 \| Vicia cracca \| JN894489.1 \| JN661198.1 \| N \|  \| Nat \| \| 1577 \| Vicia faba \| JX505862.1 \| JN661200.1 \| AN \|  \| Non-inv \| \| 1578 \| Vicia hirsuta \| AF522157.1 \| HM850459.1 \| N \|  \| Nat \| \| 1579 \| Vicia lathyroides \| JN895990.1 \| JN893461.1 \| N \|  \| Nat \| \| 1580 \| Vicia lutea \| AF522159.1 \| JN661202.1 \| N \|  \| Nat \| \| 1581 \| Vicia orobus \| JN895986.1 \| JN893682.1 \| N \|  \| Nat \| \| 1582 \| Vicia parviflora \| JX505906.1 \| HM850460.1 \| N \|  \| Nat \| \| 1583 \| Vicia sativa \| AF522160.1 \| JN661204.1 \| N \|  \| Nat \| \| 1584 \| Vicia sepium \| JN896140.1 \| JN893678.1 \| N \|  \| Nat \| \| 1585 \| Vicia sylvatica \| JX505904.1 \| JX505519.1 \| N \|  \| Nat \| \| 1586 \| Vicia tetrasperma \| HM026384.1 \| HM850463.1 \| N \|  \| Nat \| \| 1587 \| Vicia villosa \| AF522161.1 \| HM850464.1 \| AN \|  \| Non-inv \| \| 1588 \| Vinca major \| HM850836.1 \| HM850466.1 \| AN \|  \| Non-inv \| \| 1589 \| Vinca minor \| DQ660553.1 \| HQ384908.1 \| AR \|  \| Inv \| \| 1590 \| Viola arvensis \| HQ593498.1 \| HQ590331.1 \| AR \|  \| Non-inv \| \| 1591 \| Viola canina \| JN894337.1 \| JN893570.1 \| N \|  \| Nat \| \| 1592 \| Viola lactea \| JN895878.1 \| JN892152.1 \| N \|  \| Nat \| \| 1593 \| Viola lutea \| JN894505.1 \| JN892428.1 \| N \|  \| Nat \| \| 1594 \| Viola odorata \| JN895231.1 \| HM850467.1 \| N \|  \| Nat \| \| 1595 \| Viola palustris \| JN895189.1 \| HM850468.1 \| N \|  \| Nat \| \| 1596 \| Viola persicifolia \| \| T.S. \| N \|  \| Nat \| \| 1597 \| Viola reichenbachiana \| JN895230.1 \| JN893278.1 \| N \|  \| Nat \| \| 1598 \| Viola riviniana \| JN894328.1 \| JN892436.1 \| N \|  \| Nat \| \| 1599 \| Viola tricolor \| JN894507.1 \| JN891420.1 \| N \|  \| Nat \| \| 1600 \| Viscum album \| JN895000.1 \| L26078.1 \| N \|  \| Nat \| \| 1601 \| Vulpia bromoides \| JN894508.1 \| HM850470.1 \| N \|  \| Nat \| \| 1602 \| Vulpia ciliata \| JN894723.1 \| EF125157.1 \| N \|  \| Nat \| \| 1603 \| Vulpia fasciculata \| JN894722.1 \| JN892996.1 \| N \|  \| Nat \| \| 1604 \| Vulpia myuros \| AF164403.1 \| FN870411.1 \| AR \|  \| Non-inv \| \| 1605 \| Vulpia unilateralis \| HE646590.1 \| T.S. \| AN \|  \| Non-inv \| \| 1606 \| Wahlenbergia hederacea \| EU713293.1 \| EU713400.1 \| N \|  \| Nat \| \| 1607 \| Wolffia arrhiza \| AY034216.1 \| AY034254.1 \| N \|  \| Nat \| \| 1608 \| Woodsia ilvensis \| JF832293.1 \| JF832086.1 \| N \|  \| Nat \| \| 1609 \| Zannichellia palustris \| JN034096.1 \| U03725.1 \| N \|  \| Nat \| \| 1610 \| Zostera angustifolia \| \| HQ901570.1 \| N \|  \| Nat \| \| 1611 \| Zostera marina \| AB096164.1 \| U80734.1 \| N \|  \| Nat \| \| 1612 \| Zostera noltei \| JN894022.1 \| JN890770.1 \| N \|  \| Nat \| |
| --- | --- | --- | --- | --- | --- | --- | --- | --- | --- | --- | --- | --- | --- | --- | --- | --- | --- | --- | --- | --- | --- | --- | --- | --- | --- | --- | --- | --- | --- | --- | --- | --- | --- | --- | --- | --- | --- | --- | --- | --- | --- | --- | --- | --- | --- | --- | --- | --- | --- | --- | --- | --- | --- | --- | --- | --- | --- | --- | --- | --- | --- | --- | --- | --- | --- | --- | --- | --- | --- | --- | --- | --- | --- | --- | --- | --- | --- | --- | --- | --- | --- | --- | --- | --- | --- | --- | --- | --- | --- | --- | --- | --- | --- | --- | --- | --- | --- | --- | --- | --- | --- | --- | --- | --- | --- | --- | --- | --- | --- | --- | --- | --- | --- | --- | --- | --- | --- | --- | --- | --- | --- | --- | --- | --- | --- | --- | --- | --- | --- | --- | --- | --- | --- | --- | --- | --- | --- | --- | --- | --- | --- | --- | --- | --- | --- | --- | --- | --- | --- | --- | --- | --- | --- | --- | --- | --- | --- | --- | --- | --- | --- | --- | --- | --- | --- | --- | --- | --- | --- | --- | --- | --- | --- | --- | --- | --- | --- | --- | --- | --- | --- | --- | --- | --- | --- | --- | --- | --- | --- | --- | --- | --- | --- | --- | --- | --- | --- | --- | --- | --- | --- | --- | --- | --- | --- | --- | --- | --- | --- | --- | --- | --- | --- | --- | --- | --- | --- | --- | --- | --- | --- | --- | --- | --- | --- | --- | --- | --- | --- | --- | --- | --- | --- | --- | --- | --- | --- | --- | --- | --- | --- | --- | --- | --- | --- | --- | --- | --- | --- | --- | --- | --- | --- | --- | --- | --- | --- | --- | --- | --- | --- | --- | --- | --- | --- | --- | --- | --- | --- | --- | --- | --- | --- | --- | --- | --- | --- | --- | --- | --- | --- | --- | --- | --- | --- | --- | --- | --- | --- | --- | --- | --- | --- | --- | --- | --- | --- | --- | --- | --- | --- | --- | --- | --- | --- | --- | --- | --- | --- | --- | --- | --- | --- | --- | --- | --- | --- | --- | --- | --- | --- | --- | --- | --- | --- | --- | --- | --- | --- | --- | --- | --- | --- | --- | --- | --- | --- | --- | --- | --- | --- | --- | --- | --- | --- | --- | --- | --- | --- | --- | --- | --- | --- | --- | --- | --- | --- | --- | --- | --- | --- | --- | --- | --- | --- | --- | --- | --- | --- | --- | --- | --- | --- | --- | --- | --- | --- | --- | --- | --- | --- | --- | --- | --- | --- | --- | --- | --- | --- | --- | --- | --- | --- | --- | --- | --- | --- | --- | --- | --- | --- | --- | --- | --- | --- | --- | --- | --- | --- | --- | --- | --- | --- | --- | --- | --- | --- | --- | --- | --- | --- | --- | --- | --- | --- | --- | --- | --- | --- | --- | --- | --- | --- | --- | --- | --- | --- | --- | --- | --- | --- | --- | --- | --- | --- | --- | --- | --- | --- | --- | --- | --- | --- | --- | --- | --- | --- | --- | --- | --- | --- | --- | --- | --- | --- | --- | --- | --- | --- | --- | --- | --- | --- | --- | --- | --- | --- | --- | --- | --- | --- | --- | --- | --- | --- | --- | --- | --- | --- | --- | --- | --- | --- | --- | --- | --- | --- | --- | --- | --- | --- | --- | --- | --- | --- | --- | --- | --- | --- | --- | --- | --- | --- | --- | --- | --- | --- | --- | --- | --- | --- | --- | --- | --- | --- | --- | --- | --- | --- | --- | --- | --- | --- | --- | --- | --- | --- | --- | --- | --- | --- | --- | --- | --- | --- | --- | --- | --- | --- | --- | --- | --- | --- | --- | --- | --- | --- | --- | --- | --- | --- | --- | --- | --- | --- | --- | --- | --- | --- | --- | --- | --- | --- | --- | --- | --- | --- | --- | --- | --- | --- | --- | --- | --- | --- | --- | --- | --- | --- | --- | --- | --- | --- | --- | --- | --- | --- | --- | --- | --- | --- | --- | --- | --- | --- | --- | --- | --- | --- | --- | --- | --- | --- | --- | --- | --- | --- | --- | --- | --- | --- | --- | --- | --- | --- | --- | --- | --- | --- | --- | --- | --- | --- | --- | --- | --- | --- | --- | --- | --- | --- | --- | --- | --- | --- | --- | --- | --- | --- | --- | --- | --- | --- | --- | --- | --- | --- | --- | --- | --- | --- | --- | --- | --- | --- | --- | --- | --- | --- | --- | --- | --- | --- | --- | --- | --- | --- | --- | --- | --- | --- | --- | --- | --- | --- | --- | --- | --- | --- | --- | --- | --- | --- | --- | --- | --- | --- | --- | --- | --- | --- | --- | --- | --- | --- | --- | --- | --- | --- | --- | --- | --- | --- | --- | --- | --- | --- | --- | --- | --- | --- | --- | --- | --- | --- | --- | --- | --- | --- | --- | --- | --- | --- | --- | --- | --- | --- | --- | --- | --- | --- | --- | --- | --- | --- | --- | --- | --- | --- | --- | --- | --- | --- | --- | --- | --- | --- | --- | --- | --- | --- | --- | --- | --- | --- | --- | --- | --- | --- | --- | --- | --- | --- | --- | --- | --- | --- | --- | --- | --- | --- | --- | --- | --- | --- | --- | --- | --- | --- | --- | --- | --- | --- | --- | --- | --- | --- | --- | --- | --- | --- | --- | --- | --- | --- | --- | --- | --- | --- | --- | --- | --- | --- | --- | --- | --- | --- | --- | --- | --- | --- | --- | --- | --- | --- | --- | --- | --- | --- | --- | --- | --- | --- | --- | --- | --- | --- | --- | --- | --- | --- | --- | --- | --- | --- | --- | --- | --- | --- | --- | --- | --- | --- | --- | --- | --- | --- | --- | --- | --- | --- | --- | --- | --- | --- | --- | --- | --- | --- | --- | --- | --- | --- | --- | --- | --- | --- | --- | --- | --- | --- | --- | --- | --- | --- | --- | --- | --- | --- | --- | --- | --- | --- | --- | --- | --- | --- | --- | --- | --- | --- | --- | --- | --- | --- | --- | --- | --- | --- | --- | --- | --- | --- | --- | --- | --- | --- | --- | --- | --- | --- | --- | --- | --- | --- | --- | --- | --- | --- | --- | --- | --- | --- | --- | --- | --- | --- | --- | --- | --- | --- | --- | --- | --- | --- | --- | --- | --- | --- | --- | --- | --- | --- | --- | --- | --- | --- | --- | --- | --- | --- | --- | --- | --- | --- | --- | --- | --- | --- | --- | --- | --- | --- | --- | --- | --- | --- | --- | --- | --- | --- | --- | --- | --- | --- | --- | --- | --- | --- | --- | --- | --- | --- | --- | --- | --- | --- | --- | --- | --- | --- | --- | --- | --- | --- | --- | --- | --- | --- | --- | --- | --- | --- | --- | --- | --- | --- | --- | --- | --- | --- | --- | --- | --- | --- | --- | --- | --- | --- | --- | --- | --- | --- | --- | --- | --- | --- | --- | --- | --- | --- | --- | --- | --- | --- | --- | --- | --- | --- | --- | --- | --- | --- | --- | --- | --- | --- | --- | --- | --- | --- | --- | --- | --- | --- | --- | --- | --- | --- | --- | --- | --- | --- | --- | --- | --- | --- | --- | --- | --- | --- | --- | --- | --- | --- | --- | --- | --- | --- | --- | --- | --- | --- | --- | --- | --- | --- | --- | --- | --- | --- | --- | --- | --- | --- | --- | --- | --- | --- | --- | --- | --- | --- | --- | --- | --- | --- | --- | --- | --- | --- | --- | --- | --- | --- | --- | --- | --- | --- | --- | --- | --- | --- | --- | --- | --- | --- | --- | --- | --- | --- | --- | --- | --- | --- | --- | --- | --- | --- | --- | --- | --- | --- | --- | --- | --- | --- | --- | --- | --- | --- | --- | --- | --- | --- | --- | --- | --- | --- | --- | --- | --- | --- | --- | --- | --- | --- | --- | --- | --- | --- | --- | --- | --- | --- | --- | --- | --- | --- | --- | --- | --- | --- | --- | --- | --- | --- | --- | --- | --- | --- | --- | --- | --- | --- | --- | --- | --- | --- | --- | --- | --- | --- | --- | --- | --- | --- | --- | --- | --- | --- | --- | --- | --- | --- | --- | --- | --- | --- | --- | --- | --- | --- | --- | --- | --- | --- | --- | --- | --- | --- | --- | --- | --- | --- | --- | --- | --- | --- | --- | --- | --- | --- | --- | --- | --- | --- | --- | --- | --- | --- | --- | --- | --- | --- | --- | --- | --- | --- | --- | --- | --- | --- | --- | --- | --- | --- | --- | --- | --- | --- | --- | --- | --- | --- | --- | --- | --- | --- | --- | --- | --- | --- | --- | --- | --- | --- | --- | --- | --- | --- | --- | --- | --- | --- | --- | --- | --- | --- | --- | --- | --- | --- | --- | --- | --- | --- | --- | --- | --- | --- | --- | --- | --- | --- | --- | --- | --- | --- | --- | --- | --- | --- | --- | --- | --- | --- | --- | --- | --- | --- | --- | --- | --- | --- | --- | --- | --- | --- | --- | --- | --- | --- | --- | --- | --- | --- | --- | --- | --- | --- | --- | --- | --- | --- | --- | --- | --- | --- | --- | --- | --- | --- | --- | --- | --- | --- | --- | --- | --- | --- | --- | --- | --- | --- | --- | --- | --- | --- | --- | --- | --- | --- | --- | --- | --- | --- | --- | --- | --- | --- | --- | --- | --- | --- | --- | --- | --- | --- | --- | --- | --- | --- | --- | --- | --- | --- | --- | --- | --- | --- | --- | --- | --- | --- | --- | --- | --- | --- | --- | --- | --- | --- | --- | --- | --- | --- | --- | --- | --- | --- | --- | --- | --- | --- | --- | --- | --- | --- | --- | --- | --- | --- | --- | --- | --- | --- | --- | --- | --- | --- | --- | --- | --- | --- | --- | --- | --- | --- | --- | --- | --- | --- | --- | --- | --- | --- | --- | --- | --- | --- | --- | --- | --- | --- | --- | --- | --- | --- | --- | --- | --- | --- | --- | --- | --- | --- | --- | --- | --- | --- | --- | --- | --- | --- | --- | --- | --- | --- | --- | --- | --- | --- | --- | --- | --- | --- | --- | --- | --- | --- | --- | --- | --- | --- | --- | --- | --- | --- | --- | --- | --- | --- | --- | --- | --- | --- | --- | --- | --- | --- | --- | --- | --- | --- | --- | --- | --- | --- | --- | --- | --- | --- | --- | --- | --- | --- | --- | --- | --- | --- | --- | --- | --- | --- | --- | --- | --- | --- | --- | --- | --- | --- | --- | --- | --- | --- | --- | --- | --- | --- | --- | --- | --- | --- | --- | --- | --- | --- | --- | --- | --- | --- | --- | --- | --- | --- | --- | --- | --- | --- | --- | --- | --- | --- | --- | --- | --- | --- | --- | --- | --- | --- | --- | --- | --- | --- | --- | --- | --- | --- | --- | --- | --- | --- | --- | --- | --- | --- | --- | --- | --- | --- | --- | --- | --- | --- | --- | --- | --- | --- | --- | --- | --- | --- | --- | --- | --- | --- | --- | --- | --- | --- | --- | --- | --- | --- | --- | --- | --- | --- | --- | --- | --- | --- | --- | --- | --- | --- | --- | --- | --- | --- | --- | --- | --- | --- | --- | --- | --- | --- | --- | --- | --- | --- | --- | --- | --- | --- | --- | --- | --- | --- | --- | --- | --- | --- | --- | --- | --- | --- | --- | --- | --- | --- | --- | --- | --- | --- | --- | --- | --- | --- | --- | --- | --- | --- | --- | --- | --- | --- | --- | --- | --- | --- | --- | --- | --- | --- | --- | --- | --- | --- | --- | --- | --- | --- | --- | --- | --- | --- | --- | --- | --- | --- | --- | --- | --- | --- | --- | --- | --- | --- | --- | --- | --- | --- | --- | --- | --- | --- | --- | --- | --- | --- | --- | --- | --- | --- | --- | --- | --- | --- | --- | --- | --- | --- | --- | --- | --- | --- | --- | --- | --- | --- | --- | --- | --- | --- | --- | --- | --- | --- | --- | --- | --- | --- | --- | --- | --- | --- | --- | --- | --- | --- | --- | --- | --- | --- | --- | --- | --- | --- | --- | --- | --- | --- | --- | --- | --- | --- | --- | --- | --- | --- | --- | --- | --- | --- | --- | --- | --- | --- | --- | --- | --- | --- | --- | --- | --- | --- | --- | --- | --- | --- | --- | --- | --- | --- | --- | --- | --- | --- | --- | --- | --- | --- | --- | --- | --- | --- | --- | --- | --- | --- | --- | --- | --- | --- | --- | --- | --- | --- | --- | --- | --- | --- | --- | --- | --- | --- | --- | --- | --- | --- | --- | --- | --- | --- | --- | --- | --- | --- | --- | --- | --- | --- | --- | --- | --- | --- | --- | --- | --- | --- | --- | --- | --- | --- | --- | --- | --- | --- | --- | --- | --- | --- | --- | --- | --- | --- | --- | --- | --- | --- | --- | --- | --- | --- | --- | --- | --- | --- | --- | --- | --- | --- | --- | --- | --- | --- | --- | --- | --- | --- | --- | --- | --- | --- | --- | --- | --- | --- | --- | --- | --- | --- | --- | --- | --- | --- | --- | --- | --- | --- | --- | --- | --- | --- | --- | --- | --- | --- | --- | --- | --- | --- | --- | --- | --- | --- | --- | --- | --- | --- | --- | --- | --- | --- | --- | --- | --- | --- | --- | --- | --- | --- | --- | --- | --- | --- | --- | --- | --- | --- | --- | --- | --- | --- | --- | --- | --- | --- | --- | --- | --- | --- | --- | --- | --- | --- | --- | --- | --- | --- | --- | --- | --- | --- | --- | --- | --- | --- | --- | --- | --- | --- | --- | --- | --- | --- | --- | --- | --- | --- | --- | --- | --- | --- | --- | --- | --- | --- | --- | --- | --- | --- | --- | --- | --- | --- | --- | --- | --- | --- | --- | --- | --- | --- | --- | --- | --- | --- | --- | --- | --- | --- | --- | --- | --- | --- | --- | --- | --- | --- | --- | --- | --- | --- | --- | --- | --- | --- | --- | --- | --- | --- | --- | --- | --- | --- | --- | --- | --- | --- | --- | --- | --- | --- | --- | --- | --- | --- | --- | --- | --- | --- | --- | --- | --- | --- | --- | --- | --- | --- | --- | --- | --- | --- | --- | --- | --- | --- | --- | --- | --- | --- | --- | --- | --- | --- | --- | --- | --- | --- | --- | --- | --- | --- | --- | --- | --- | --- | --- | --- | --- | --- | --- | --- | --- | --- | --- | --- | --- | --- | --- | --- | --- | --- | --- | --- | --- | --- | --- | --- | --- | --- | --- | --- | --- | --- | --- | --- | --- | --- | --- | --- | --- | --- | --- | --- | --- | --- | --- | --- | --- | --- | --- | --- | --- | --- | --- | --- | --- | --- | --- | --- | --- | --- | --- | --- | --- | --- | --- | --- | --- | --- | --- | --- | --- | --- | --- | --- | --- | --- | --- | --- | --- | --- | --- | --- | --- | --- | --- | --- | --- | --- | --- | --- | --- | --- | --- | --- | --- | --- | --- | --- | --- | --- | --- | --- | --- | --- | --- | --- | --- | --- | --- | --- | --- | --- | --- | --- | --- | --- | --- | --- | --- | --- | --- | --- | --- | --- | --- | --- | --- | --- | --- | --- | --- | --- | --- | --- | --- | --- | --- | --- | --- | --- | --- | --- | --- | --- | --- | --- | --- | --- | --- | --- | --- | --- | --- | --- | --- | --- | --- | --- | --- | --- | --- | --- | --- | --- | --- | --- | --- | --- | --- | --- | --- | --- | --- | --- | --- | --- | --- | --- | --- | --- | --- | --- | --- | --- | --- | --- | --- | --- | --- | --- | --- | --- | --- | --- | --- | --- | --- | --- | --- | --- | --- | --- | --- | --- | --- | --- | --- | --- | --- | --- | --- | --- | --- | --- | --- | --- | --- | --- | --- | --- | --- | --- | --- | --- | --- | --- | --- | --- | --- | --- | --- | --- | --- | --- | --- | --- | --- | --- | --- | --- | --- | --- | --- | --- | --- | --- | --- | --- | --- | --- | --- | --- | --- | --- | --- | --- | --- | --- | --- | --- | --- | --- | --- | --- | --- | --- | --- | --- | --- | --- | --- | --- | --- | --- | --- | --- | --- | --- | --- | --- | --- | --- | --- | --- | --- | --- | --- | --- | --- | --- | --- | --- | --- | --- | --- | --- | --- | --- | --- | --- | --- | --- | --- | --- | --- | --- | --- | --- | --- | --- | --- | --- | --- | --- | --- | --- | --- | --- | --- | --- | --- | --- | --- | --- | --- | --- | --- | --- | --- | --- | --- | --- | --- | --- | --- | --- | --- | --- | --- | --- | --- | --- | --- | --- | --- | --- | --- | --- | --- | --- | --- | --- | --- | --- | --- | --- | --- | --- | --- | --- | --- | --- | --- | --- | --- | --- | --- | --- | --- | --- | --- | --- | --- | --- | --- | --- | --- | --- | --- | --- | --- | --- | --- | --- | --- | --- | --- | --- | --- | --- | --- | --- | --- | --- | --- | --- | --- | --- | --- | --- | --- | --- | --- | --- | --- | --- | --- | --- | --- | --- | --- | --- | --- | --- | --- | --- | --- | --- | --- | --- | --- | --- | --- | --- | --- | --- | --- | --- | --- | --- | --- | --- | --- | --- | --- | --- | --- | --- | --- | --- | --- | --- | --- | --- | --- | --- | --- | --- | --- | --- | --- | --- | --- | --- | --- | --- | --- | --- | --- | --- | --- | --- | --- | --- | --- | --- | --- | --- | --- | --- | --- | --- | --- | --- | --- | --- | --- | --- | --- | --- | --- | --- | --- | --- | --- | --- | --- | --- | --- | --- | --- | --- | --- | --- | --- | --- | --- | --- | --- | --- | --- | --- | --- | --- | --- | --- | --- | --- | --- | --- | --- | --- | --- | --- | --- | --- | --- | --- | --- | --- | --- | --- | --- | --- | --- | --- | --- | --- | --- | --- | --- | --- | --- | --- | --- | --- | --- | --- | --- | --- | --- | --- | --- | --- | --- | --- | --- | --- | --- | --- | --- | --- | --- | --- | --- | --- | --- | --- | --- | --- | --- | --- | --- | --- | --- | --- | --- | --- | --- | --- | --- | --- | --- | --- | --- | --- | --- | --- | --- | --- | --- | --- | --- | --- | --- | --- | --- | --- | --- | --- | --- | --- | --- | --- | --- | --- | --- | --- | --- | --- | --- | --- | --- | --- | --- | --- | --- | --- | --- | --- | --- | --- | --- | --- | --- | --- | --- | --- | --- | --- | --- | --- | --- | --- | --- | --- | --- | --- | --- | --- | --- | --- | --- | --- | --- | --- | --- | --- | --- | --- | --- | --- | --- | --- | --- | --- | --- | --- | --- | --- | --- | --- | --- | --- | --- | --- | --- | --- | --- | --- | --- | --- | --- | --- | --- | --- | --- | --- | --- | --- | --- | --- | --- | --- | --- | --- | --- | --- | --- | --- | --- | --- | --- | --- | --- | --- | --- | --- | --- | --- | --- | --- | --- | --- | --- | --- | --- | --- | --- | --- | --- | --- | --- | --- | --- | --- | --- | --- | --- | --- | --- | --- | --- | --- | --- | --- | --- | --- | --- | --- | --- | --- | --- | --- | --- | --- | --- | --- | --- | --- | --- | --- | --- | --- | --- | --- | --- | --- | --- | --- | --- | --- | --- | --- | --- | --- | --- | --- | --- | --- | --- | --- | --- | --- | --- | --- | --- | --- | --- | --- | --- | --- | --- | --- | --- | --- | --- | --- | --- | --- | --- | --- | --- | --- | --- | --- | --- | --- | --- | --- | --- | --- | --- | --- | --- | --- | --- | --- | --- | --- | --- | --- | --- | --- | --- | --- | --- | --- | --- | --- | --- | --- | --- | --- | --- | --- | --- | --- | --- | --- | --- | --- | --- | --- | --- | --- | --- | --- | --- | --- | --- | --- | --- | --- | --- | --- | --- | --- | --- | --- | --- | --- | --- | --- | --- | --- | --- | --- | --- | --- | --- | --- | --- | --- | --- | --- | --- | --- | --- | --- | --- | --- | --- | --- | --- | --- | --- | --- | --- | --- | --- | --- | --- | --- | --- | --- | --- | --- | --- | --- | --- | --- | --- | --- | --- | --- | --- | --- | --- | --- | --- | --- | --- | --- | --- | --- | --- | --- | --- | --- | --- | --- | --- | --- | --- | --- | --- | --- | --- | --- | --- | --- | --- | --- | --- | --- | --- | --- | --- | --- | --- | --- | --- | --- | --- | --- | --- | --- | --- | --- | --- | --- | --- | --- | --- | --- | --- | --- | --- | --- | --- | --- | --- | --- | --- | --- | --- | --- | --- | --- | --- | --- | --- | --- | --- | --- | --- | --- | --- | --- | --- | --- | --- | --- | --- | --- | --- | --- | --- | --- | --- | --- | --- | --- | --- | --- | --- | --- | --- | --- | --- | --- | --- | --- | --- | --- | --- | --- | --- | --- | --- | --- | --- | --- | --- | --- | --- | --- | --- | --- | --- | --- | --- | --- | --- | --- | --- | --- | --- | --- | --- | --- | --- | --- | --- | --- | --- | --- | --- | --- | --- | --- | --- | --- | --- | --- | --- | --- | --- | --- | --- | --- | --- | --- | --- | --- | --- | --- | --- | --- | --- | --- | --- | --- | --- | --- | --- | --- | --- | --- | --- | --- | --- | --- | --- | --- | --- | --- | --- | --- | --- | --- | --- | --- | --- | --- | --- | --- | --- | --- | --- | --- | --- | --- | --- | --- | --- | --- | --- | --- | --- | --- | --- | --- | --- | --- | --- | --- | --- | --- | --- | --- | --- | --- | --- | --- | --- | --- | --- | --- | --- | --- | --- | --- | --- | --- | --- | --- | --- | --- | --- | --- | --- | --- | --- | --- | --- | --- | --- | --- | --- | --- | --- | --- | --- | --- | --- | --- | --- | --- | --- | --- | --- | --- | --- | --- | --- | --- | --- | --- | --- | --- | --- | --- | --- | --- | --- | --- | --- | --- | --- | --- | --- | --- | --- | --- | --- | --- | --- | --- | --- | --- | --- | --- | --- | --- | --- | --- | --- | --- | --- | --- | --- | --- | --- | --- | --- | --- | --- | --- | --- | --- | --- | --- | --- | --- | --- | --- | --- | --- | --- | --- | --- | --- | --- | --- | --- | --- | --- | --- | --- | --- | --- | --- | --- | --- | --- | --- | --- | --- | --- | --- | --- | --- | --- | --- | --- | --- | --- | --- | --- | --- | --- | --- | --- | --- | --- | --- | --- | --- | --- | --- | --- | --- | --- | --- | --- | --- | --- | --- | --- | --- | --- | --- | --- | --- | --- | --- | --- | --- | --- | --- | --- | --- | --- | --- | --- | --- | --- | --- | --- | --- | --- | --- | --- | --- | --- | --- | --- | --- | --- | --- | --- | --- | --- | --- | --- | --- | --- | --- | --- | --- | --- | --- | --- | --- | --- | --- | --- | --- | --- | --- | --- | --- | --- | --- | --- | --- | --- | --- | --- | --- | --- | --- | --- | --- | --- | --- | --- | --- | --- | --- | --- | --- | --- | --- | --- | --- | --- | --- | --- | --- | --- | --- | --- | --- | --- | --- | --- | --- | --- | --- | --- | --- | --- | --- | --- | --- | --- | --- | --- | --- | --- | --- | --- | --- | --- | --- | --- | --- | --- | --- | --- | --- | --- | --- | --- | --- | --- | --- | --- | --- | --- | --- | --- | --- | --- | --- | --- | --- | --- | --- | --- | --- | --- | --- | --- | --- | --- | --- | --- | --- | --- | --- | --- | --- | --- | --- | --- | --- | --- | --- | --- | --- | --- | --- | --- | --- | --- | --- | --- | --- | --- | --- | --- | --- | --- | --- | --- | --- | --- | --- | --- | --- | --- | --- | --- | --- | --- | --- | --- | --- | --- | --- | --- | --- | --- | --- | --- | --- | --- | --- | --- | --- | --- | --- | --- | --- | --- | --- | --- | --- | --- | --- | --- | --- | --- | --- | --- | --- | --- | --- | --- | --- | --- | --- | --- | --- | --- | --- | --- | --- | --- | --- | --- | --- | --- | --- | --- | --- | --- | --- | --- | --- | --- | --- | --- | --- | --- | --- | --- | --- | --- | --- | --- | --- | --- | --- | --- | --- | --- | --- | --- | --- | --- | --- | --- | --- | --- | --- | --- | --- | --- | --- | --- | --- | --- | --- | --- | --- | --- | --- | --- | --- | --- | --- | --- | --- | --- | --- | --- | --- | --- | --- | --- | --- | --- | --- | --- | --- | --- | --- | --- | --- | --- | --- | --- | --- | --- | --- | --- | --- | --- | --- | --- | --- | --- | --- | --- | --- | --- | --- | --- | --- | --- | --- | --- | --- | --- | --- | --- | --- | --- | --- | --- | --- | --- | --- | --- | --- | --- | --- | --- | --- | --- | --- | --- | --- | --- | --- | --- | --- | --- | --- | --- | --- | --- | --- | --- | --- | --- | --- | --- | --- | --- | --- | --- | --- | --- | --- | --- | --- | --- | --- | --- | --- | --- | --- | --- | --- | --- | --- | --- | --- | --- | --- | --- | --- | --- | --- | --- | --- | --- | --- | --- | --- | --- | --- | --- | --- | --- | --- | --- | --- | --- | --- | --- | --- | --- | --- | --- | --- | --- | --- | --- | --- | --- | --- | --- | --- | --- | --- | --- | --- | --- | --- | --- | --- | --- | --- | --- | --- | --- | --- | --- | --- | --- | --- | --- | --- | --- | --- | --- | --- | --- | --- | --- | --- | --- | --- | --- | --- | --- | --- | --- | --- | --- | --- | --- | --- | --- | --- | --- | --- | --- | --- | --- | --- | --- | --- | --- | --- | --- | --- | --- | --- | --- | --- | --- | --- | --- | --- | --- | --- | --- | --- | --- | --- | --- | --- | --- | --- | --- | --- | --- | --- | --- | --- | --- | --- | --- | --- | --- | --- | --- | --- | --- | --- | --- | --- | --- | --- | --- | --- | --- | --- | --- | --- | --- | --- | --- | --- | --- | --- | --- | --- | --- | --- | --- | --- | --- | --- | --- | --- | --- | --- | --- | --- | --- | --- | --- | --- | --- | --- | --- | --- | --- | --- | --- | --- | --- | --- | --- | --- | --- | --- | --- | --- | --- | --- | --- | --- | --- | --- | --- | --- | --- | --- | --- | --- | --- | --- | --- | --- | --- | --- | --- | --- | --- | --- | --- | --- | --- | --- | --- | --- | --- | --- | --- | --- | --- | --- | --- | --- | --- | --- | --- | --- | --- | --- | --- | --- | --- | --- | --- | --- | --- | --- | --- | --- | --- | --- | --- | --- | --- | --- | --- | --- | --- | --- | --- | --- | --- | --- | --- | --- | --- | --- | --- | --- | --- | --- | --- | --- | --- | --- | --- | --- | --- | --- | --- | --- | --- | --- | --- | --- | --- | --- | --- | --- | --- | --- | --- | --- | --- | --- | --- | --- | --- | --- | --- | --- | --- | --- | --- | --- | --- | --- | --- | --- | --- | --- | --- | --- | --- | --- | --- | --- | --- | --- | --- | --- | --- | --- | --- | --- | --- | --- | --- | --- | --- | --- | --- | --- | --- | --- | --- | --- | --- | --- | --- | --- | --- | --- | --- | --- | --- | --- | --- | --- | --- | --- | --- | --- | --- | --- | --- | --- | --- | --- | --- | --- | --- | --- | --- | --- | --- | --- | --- | --- | --- | --- | --- | --- | --- | --- | --- | --- | --- | --- | --- | --- | --- | --- | --- | --- | --- | --- | --- | --- | --- | --- | --- | --- | --- | --- | --- | --- | --- | --- | --- | --- | --- | --- | --- | --- | --- | --- | --- | --- | --- | --- | --- | --- | --- | --- | --- | --- | --- | --- | --- | --- | --- | --- | --- | --- | --- | --- | --- | --- | --- | --- | --- | --- | --- | --- | --- | --- | --- | --- | --- | --- | --- | --- | --- | --- | --- | --- | --- | --- | --- | --- | --- | --- | --- | --- | --- | --- | --- | --- | --- | --- | --- | --- | --- | --- | --- | --- | --- | --- | --- | --- | --- | --- | --- | --- | --- | --- | --- | --- | --- | --- | --- | --- | --- | --- | --- | --- | --- | --- | --- | --- | --- | --- | --- | --- | --- | --- | --- | --- | --- | --- | --- | --- | --- | --- | --- | --- | --- | --- | --- | --- | --- | --- | --- | --- | --- | --- | --- | --- | --- | --- | --- | --- | --- | --- | --- | --- | --- | --- | --- | --- | --- | --- | --- | --- | --- | --- | --- | --- | --- | --- | --- | --- | --- | --- | --- | --- | --- | --- | --- | --- | --- | --- | --- | --- | --- | --- | --- | --- | --- | --- | --- | --- | --- | --- | --- | --- | --- | --- | --- | --- | --- | --- | --- | --- | --- | --- | --- | --- | --- | --- | --- | --- | --- | --- | --- | --- | --- | --- | --- | --- | --- | --- | --- | --- | --- | --- | --- | --- | --- | --- | --- | --- | --- | --- | --- | --- | --- | --- | --- | --- | --- | --- | --- | --- | --- | --- | --- | --- | --- | --- | --- | --- | --- | --- | --- | --- | --- | --- | --- | --- | --- | --- | --- | --- | --- | --- | --- | --- | --- | --- | --- | --- | --- | --- | --- | --- | --- | --- | --- | --- | --- | --- | --- | --- | --- | --- | --- | --- | --- | --- | --- | --- | --- | --- | --- | --- | --- | --- | --- | --- | --- | --- | --- | --- | --- | --- | --- | --- | --- | --- | --- | --- | --- | --- | --- | --- | --- | --- | --- | --- | --- | --- | --- | --- | --- | --- | --- | --- | --- | --- | --- | --- | --- | --- | --- | --- | --- | --- | --- | --- | --- | --- | --- | --- | --- | --- | --- | --- | --- | --- | --- | --- | --- | --- | --- | --- | --- | --- | --- | --- | --- | --- | --- | --- | --- | --- | --- | --- | --- | --- | --- | --- | --- | --- | --- | --- | --- | --- | --- | --- | --- | --- | --- | --- | --- | --- | --- | --- | --- | --- | --- | --- | --- | --- | --- | --- | --- | --- | --- | --- | --- | --- | --- | --- | --- | --- | --- | --- | --- | --- | --- | --- | --- | --- | --- | --- | --- | --- | --- | --- | --- | --- | --- | --- | --- | --- | --- | --- | --- | --- | --- | --- | --- | --- | --- | --- | --- | --- | --- | --- | --- | --- | --- | --- | --- | --- | --- | --- | --- | --- | --- | --- | --- | --- | --- | --- | --- | --- | --- | --- | --- | --- | --- | --- | --- | --- | --- | --- | --- | --- | --- | --- | --- | --- | --- | --- | --- | --- | --- | --- | --- | --- | --- | --- | --- | --- | --- | --- | --- | --- | --- | --- | --- | --- | --- | --- | --- | --- | --- | --- | --- | --- | --- | --- | --- | --- | --- | --- | --- | --- | --- | --- | --- | --- | --- | --- | --- | --- | --- | --- | --- | --- | --- | --- | --- | --- | --- | --- | --- | --- | --- | --- | --- | --- | --- | --- | --- | --- | --- | --- | --- | --- | --- | --- | --- | --- | --- | --- | --- | --- | --- | --- | --- | --- | --- | --- | --- | --- | --- | --- | --- | --- | --- | --- | --- | --- | --- | --- | --- | --- | --- | --- | --- | --- | --- | --- | --- | --- | --- | --- | --- | --- | --- | --- | --- | --- | --- | --- | --- | --- | --- | --- | --- | --- | --- | --- | --- | --- | --- | --- | --- | --- | --- | --- | --- | --- | --- | --- | --- | --- | --- | --- | --- | --- | --- | --- | --- | --- | --- | --- | --- | --- | --- | --- | --- | --- | --- | --- | --- | --- | --- | --- | --- | --- | --- | --- | --- | --- | --- | --- | --- | --- | --- | --- | --- | --- | --- | --- | --- | --- | --- | --- | --- | --- | --- | --- | --- | --- | --- | --- | --- | --- | --- | --- | --- | --- | --- | --- | --- | --- | --- | --- | --- | --- | --- | --- | --- | --- | --- | --- | --- | --- | --- | --- | --- | --- | --- | --- | --- | --- | --- | --- | --- | --- | --- | --- | --- | --- | --- | --- | --- | --- | --- | --- | --- | --- | --- | --- | --- | --- | --- | --- | --- | --- | --- | --- | --- | --- | --- | --- | --- | --- | --- | --- | --- | --- | --- | --- | --- | --- | --- | --- | --- | --- | --- | --- | --- | --- | --- | --- | --- | --- | --- | --- | --- | --- | --- | --- | --- | --- | --- | --- | --- | --- | --- | --- | --- | --- | --- | --- | --- | --- | --- | --- | --- | --- | --- | --- | --- | --- | --- | --- | --- | --- | --- | --- | --- | --- | --- | --- | --- | --- | --- | --- | --- | --- | --- | --- | --- | --- | --- | --- | --- | --- | --- | --- | --- | --- | --- | --- | --- | --- | --- | --- | --- | --- | --- | --- | --- | --- | --- | --- | --- | --- | --- | --- | --- | --- | --- | --- | --- | --- | --- | --- | --- | --- | --- | --- | --- | --- | --- | --- | --- | --- | --- | --- | --- | --- | --- | --- | --- | --- | --- | --- | --- | --- | --- | --- | --- | --- | --- | --- | --- | --- | --- | --- | --- | --- | --- | --- | --- | --- | --- | --- | --- | --- | --- | --- | --- | --- | --- | --- | --- | --- | --- | --- | --- | --- | --- | --- | --- | --- | --- | --- | --- | --- | --- | --- | --- | --- | --- | --- | --- | --- | --- | --- | --- | --- | --- | --- | --- | --- | --- | --- | --- | --- | --- | --- | --- | --- | --- | --- | --- | --- | --- | --- | --- | --- | --- | --- | --- | --- | --- | --- | --- | --- | --- | --- | --- | --- | --- | --- | --- | --- | --- | --- | --- | --- | --- | --- | --- | --- | --- | --- | --- | --- | --- | --- | --- | --- | --- | --- | --- | --- | --- | --- | --- | --- | --- | --- | --- | --- | --- | --- | --- | --- | --- | --- | --- | --- | --- | --- | --- | --- | --- | --- | --- | --- | --- | --- | --- | --- | --- | --- | --- | --- | --- | --- | --- | --- | --- | --- | --- | --- | --- | --- | --- | --- | --- | --- | --- | --- | --- | --- | --- | --- | --- | --- | --- | --- | --- | --- | --- | --- | --- | --- | --- | --- | --- | --- | --- | --- | --- | --- | --- | --- | --- | --- | --- | --- | --- | --- | --- | --- | --- | --- | --- | --- | --- | --- | --- | --- | --- | --- | --- | --- | --- | --- | --- | --- | --- | --- | --- | --- | --- | --- | --- | --- | --- | --- | --- | --- | --- | --- | --- | --- | --- | --- | --- | --- | --- | --- | --- | --- | --- | --- | --- | --- | --- | --- | --- | --- | --- | --- | --- | --- | --- | --- | --- | --- | --- | --- | --- | --- | --- | --- | --- | --- | --- | --- | --- | --- | --- | --- | --- | --- | --- | --- | --- | --- | --- | --- | --- | --- | --- | --- | --- | --- | --- | --- | --- | --- | --- | --- | --- | --- | --- | --- | --- | --- | --- | --- | --- | --- | --- | --- | --- | --- | --- | --- | --- | --- | --- | --- | --- | --- | --- | --- | --- | --- | --- | --- | --- | --- | --- | --- | --- | --- | --- | --- | --- | --- | --- | --- | --- | --- | --- | --- | --- | --- | --- | --- | --- | --- | --- | --- | --- | --- | --- | --- | --- | --- | --- | --- | --- | --- | --- | --- | --- | --- | --- | --- | --- | --- | --- | --- | --- | --- | --- | --- | --- | --- | --- | --- | --- | --- | --- | --- | --- | --- | --- | --- | --- | --- | --- | --- | --- | --- | --- | --- | --- | --- | --- | --- | --- | --- | --- | --- | --- | --- | --- | --- | --- | --- | --- | --- | --- | --- | --- | --- | --- | --- | --- | --- | --- | --- | --- | --- | --- | --- | --- | --- | --- | --- | --- | --- | --- | --- | --- | --- | --- | --- | --- | --- | --- | --- | --- | --- | --- | --- | --- | --- | --- | --- | --- | --- | --- | --- | --- | --- | --- | --- | --- | --- | --- | --- | --- | --- | --- | --- | --- | --- | --- | --- | --- | --- | --- | --- | --- | --- | --- | --- | --- | --- | --- | --- | --- | --- | --- | --- | --- | --- | --- | --- | --- | --- | --- | --- | --- | --- | --- | --- | --- | --- | --- | --- | --- | --- | --- | --- | --- | --- | --- | --- | --- | --- | --- | --- | --- | --- | --- | --- | --- | --- | --- | --- | --- | --- | --- | --- | --- | --- | --- | --- | --- | --- | --- | --- | --- | --- | --- | --- | --- | --- | --- | --- | --- | --- | --- | --- | --- | --- | --- | --- | --- | --- | --- | --- | --- | --- | --- | --- | --- | --- | --- | --- | --- | --- | --- | --- | --- | --- | --- | --- | --- | --- | --- | --- | --- | --- | --- | --- | --- | --- | --- | --- | --- | --- | --- | --- | --- | --- | --- | --- | --- | --- | --- | --- | --- | --- | --- | --- | --- | --- | --- | --- | --- | --- | --- | --- | --- | --- | --- | --- | --- | --- | --- | --- | --- | --- | --- | --- | --- | --- | --- | --- | --- | --- | --- | --- | --- | --- | --- | --- | --- | --- | --- | --- | --- | --- | --- | --- | --- | --- | --- | --- | --- | --- | --- | --- | --- | --- | --- | --- | --- | --- | --- | --- | --- | --- | --- | --- | --- | --- | --- | --- | --- | --- | --- | --- | --- | --- | --- | --- | --- | --- | --- | --- | --- | --- | --- | --- | --- | --- | --- | --- | --- | --- | --- | --- | --- | --- | --- | --- | --- | --- | --- | --- | --- | --- | --- | --- | --- | --- | --- | --- | --- | --- | --- | --- | --- | --- | --- | --- | --- | --- | --- | --- | --- | --- | --- | --- | --- | --- | --- | --- | --- | --- | --- | --- | --- | --- | --- | --- | --- | --- | --- | --- | --- | --- | --- | --- | --- | --- | --- | --- | --- | --- | --- | --- | --- | --- | --- | --- | --- | --- | --- | --- | --- | --- | --- | --- | --- | --- | --- | --- | --- | --- | --- | --- | --- | --- | --- | --- | --- | --- | --- | --- | --- | --- | --- | --- | --- | --- | --- | --- | --- | --- | --- | --- | --- | --- | --- | --- | --- | --- | --- | --- | --- | --- | --- | --- | --- | --- | --- | --- | --- | --- | --- | --- | --- | --- | --- | --- | --- | --- | --- | --- | --- | --- | --- | --- | --- | --- | --- | --- | --- | --- | --- | --- | --- | --- | --- | --- | --- | --- | --- | --- | --- | --- | --- | --- | --- | --- | --- | --- | --- | --- | --- | --- | --- | --- | --- | --- | --- | --- | --- | --- | --- | --- | --- | --- | --- | --- | --- | --- | --- | --- | --- | --- | --- | --- | --- | --- | --- | --- | --- | --- | --- | --- | --- | --- | --- | --- | --- | --- | --- | --- | --- | --- | --- | --- | --- | --- | --- | --- | --- | --- | --- | --- | --- | --- | --- | --- | --- | --- | --- | --- | --- | --- | --- | --- | --- | --- | --- | --- | --- | --- | --- | --- | --- | --- | --- | --- | --- | --- | --- | --- | --- | --- | --- | --- | --- | --- | --- | --- | --- | --- | --- | --- | --- | --- | --- | --- | --- | --- | --- | --- | --- | --- | --- | --- | --- | --- | --- | --- | --- | --- | --- | --- | --- | --- | --- | --- | --- | --- | --- | --- | --- | --- | --- | --- | --- | --- | --- | --- | --- | --- | --- | --- | --- | --- | --- | --- | --- | --- | --- | --- | --- | --- | --- | --- | --- | --- | --- | --- | --- | --- | --- | --- | --- | --- | --- | --- | --- | --- | --- | --- | --- | --- | --- | --- | --- | --- | --- | --- | --- | --- | --- | --- | --- | --- | --- | --- | --- | --- | --- | --- | --- | --- | --- | --- | --- | --- | --- | --- | --- | --- | --- | --- | --- | --- | --- | --- | --- | --- | --- | --- | --- | --- | --- | --- | --- | --- | --- | --- | --- | --- | --- | --- | --- | --- | --- | --- | --- | --- | --- | --- | --- | --- | --- | --- | --- | --- | --- | --- | --- | --- | --- | --- | --- | --- | --- | --- | --- | --- | --- | --- | --- | --- | --- | --- | --- | --- | --- | --- | --- | --- | --- | --- | --- | --- | --- | --- | --- | --- | --- | --- | --- | --- | --- | --- | --- | --- | --- | --- | --- | --- | --- | --- | --- | --- | --- | --- | --- | --- | --- | --- | --- | --- | --- | --- | --- | --- | --- | --- | --- | --- | --- | --- | --- | --- | --- | --- | --- | --- | --- | --- | --- | --- | --- | --- | --- | --- | --- | --- | --- | --- | --- | --- | --- | --- | --- | --- | --- | --- | --- | --- | --- | --- | --- | --- | --- | --- | --- | --- | --- | --- | --- | --- | --- | --- | --- | --- | --- | --- | --- | --- | --- | --- | --- | --- | --- | --- | --- | --- | --- | --- | --- | --- | --- | --- | --- | --- | --- | --- | --- | --- | --- | --- | --- | --- | --- | --- | --- | --- | --- | --- | --- | --- | --- | --- | --- | --- | --- | --- | --- | --- | --- | --- | --- | --- | --- | --- | --- | --- | --- | --- | --- | --- | --- | --- | --- | --- | --- | --- | --- | --- | --- | --- | --- | --- | --- | --- | --- | --- | --- | --- | --- | --- | --- | --- | --- | --- | --- | --- | --- | --- | --- | --- | --- | --- | --- | --- | --- | --- | --- | --- | --- | --- | --- | --- | --- | --- | --- | --- | --- | --- | --- | --- | --- | --- | --- | --- | --- | --- | --- | --- | --- | --- | --- | --- | --- | --- | --- | --- | --- | --- | --- | --- | --- | --- | --- | --- | --- | --- | --- | --- | --- | --- | --- | --- | --- | --- | --- | --- | --- | --- | --- | --- | --- | --- | --- | --- | --- | --- | --- | --- | --- | --- | --- | --- | --- | --- | --- | --- | --- | --- | --- | --- | --- | --- | --- | --- | --- | --- | --- | --- | --- | --- | --- | --- | --- | --- | --- | --- | --- | --- | --- | --- | --- | --- | --- | --- | --- | --- | --- | --- | --- | --- | --- | --- | --- | --- | --- | --- | --- | --- | --- | --- | --- | --- | --- | --- | --- | --- | --- | --- | --- | --- | --- | --- | --- | --- | --- | --- | --- | --- | --- | --- | --- | --- | --- | --- | --- | --- | --- | --- | --- | --- | --- | --- | --- | --- | --- | --- | --- | --- | --- | --- | --- | --- | --- | --- | --- | --- | --- | --- | --- | --- | --- | --- | --- | --- | --- | --- | --- | --- | --- | --- | --- | --- | --- | --- | --- | --- | --- | --- | --- | --- | --- | --- | --- | --- | --- | --- | --- | --- | --- | --- | --- | --- | --- | --- | --- | --- | --- | --- | --- | --- | --- | --- | --- | --- | --- | --- | --- | --- | --- | --- | --- | --- | --- | --- | --- | --- | --- | --- | --- | --- | --- | --- | --- | --- | --- | --- | --- | --- | --- | --- | --- | --- | --- | --- | --- | --- | --- | --- | --- | --- | --- | --- | --- | --- | --- | --- | --- | --- | --- | --- | --- | --- | --- | --- | --- | --- | --- | --- | --- | --- | --- | --- | --- | --- | --- | --- | --- | --- | --- | --- | --- | --- | --- | --- | --- | --- | --- | --- | --- | --- | --- | --- | --- | --- | --- | --- | --- | --- | --- | --- | --- | --- | --- | --- | --- | --- | --- | --- | --- | --- | --- | --- | --- | --- | --- | --- | --- | --- | --- | --- | --- | --- | --- | --- | --- | --- | --- | --- | --- | --- | --- | --- | --- | --- | --- | --- | --- | --- | --- | --- | --- | --- | --- | --- | --- | --- | --- | --- | --- | --- | --- | --- | --- | --- | --- | --- | --- | --- | --- | --- | --- | --- | --- | --- | --- | --- | --- | --- | --- | --- | --- | --- | --- | --- | --- | --- | --- | --- | --- | --- | --- | --- | --- | --- | --- | --- | --- | --- | --- | --- | --- | --- | --- | --- | --- | --- | --- | --- | --- | --- | --- | --- | --- | --- | --- | --- | --- | --- | --- | --- | --- | --- | --- | --- | --- | --- | --- | --- | --- | --- | --- | --- | --- | --- | --- | --- | --- | --- | --- | --- | --- | --- | --- | --- | --- | --- | --- | --- | --- | --- | --- | --- | --- | --- | --- | --- | --- | --- | --- | --- | --- | --- | --- | --- | --- | --- | --- | --- | --- | --- | --- | --- | --- | --- | --- | --- | --- | --- | --- | --- | --- | --- | --- | --- | --- | --- | --- | --- | --- | --- | --- | --- | --- | --- | --- | --- | --- | --- | --- | --- | --- | --- | --- | --- | --- | --- | --- | --- | --- | --- | --- | --- | --- | --- | --- | --- | --- | --- | --- | --- | --- | --- | --- | --- | --- | --- | --- | --- | --- | --- | --- | --- | --- | --- | --- | --- | --- | --- | --- | --- | --- | --- | --- | --- | --- | --- | --- | --- | --- | --- | --- | --- | --- | --- | --- | --- | --- | --- | --- | --- | --- | --- | --- | --- | --- | --- | --- | --- | --- | --- | --- | --- | --- | --- | --- | --- | --- | --- | --- | --- | --- | --- | --- | --- | --- | --- | --- | --- | --- | --- | --- | --- | --- | --- | --- | --- | --- | --- | --- | --- | --- | --- | --- | --- | --- | --- | --- | --- | --- | --- | --- | --- | --- | --- | --- | --- | --- | --- | --- | --- | --- | --- | --- | --- | --- | --- | --- | --- | --- | --- | --- | --- | --- | --- | --- | --- | --- | --- | --- | --- | --- | --- | --- | --- | --- | --- | --- | --- | --- | --- | --- | --- | --- | --- | --- | --- | --- | --- | --- | --- | --- | --- | --- | --- | --- | --- | --- | --- | --- | --- | --- | --- | --- | --- | --- | --- | --- | --- | --- | --- | --- | --- | --- | --- | --- | --- | --- | --- | --- | --- | --- | --- | --- | --- | --- | --- | --- | --- | --- | --- | --- | --- | --- | --- | --- | --- | --- | --- | --- | --- | --- | --- | --- | --- | --- | --- | --- | --- | --- | --- | --- | --- | --- | --- | --- | --- | --- | --- | --- | --- | --- | --- | --- | --- | --- | --- | --- | --- | --- | --- | --- | --- | --- | --- | --- | --- | --- | --- | --- | --- | --- | --- | --- | --- | --- | --- | --- | --- | --- | --- | --- | --- | --- | --- | --- | --- | --- | --- | --- | --- | --- | --- | --- | --- | --- | --- | --- | --- | --- | --- | --- | --- | --- | --- | --- | --- | --- | --- | --- | --- | --- | --- | --- | --- | --- | --- | --- | --- | --- | --- | --- | --- | --- | --- | --- | --- | --- | --- | --- | --- | --- | --- | --- | --- | --- | --- | --- | --- | --- | --- | --- | --- | --- | --- | --- | --- | --- | --- | --- | --- | --- | --- | --- | --- | --- | --- | --- | --- | --- | --- | --- | --- | --- | --- | --- | --- | --- | --- | --- | --- | --- | --- | --- | --- | --- | --- | --- | --- | --- | --- | --- | --- | --- | --- | --- | --- | --- | --- | --- | --- | --- | --- | --- | --- | --- | --- | --- | --- | --- | --- | --- | --- | --- | --- | --- | --- | --- | --- | --- | --- | --- | --- | --- | --- | --- | --- | --- | --- | --- | --- | --- | --- | --- | --- | --- | --- | --- | --- | --- | --- | --- | --- | --- | --- | --- | --- | --- | --- | --- | --- | --- | --- | --- | --- | --- | --- | --- | --- | --- | --- | --- | --- | --- | --- | --- | --- | --- | --- | --- | --- | --- | --- | --- | --- | --- | --- | --- | --- | --- | --- | --- | --- | --- | --- | --- | --- | --- | --- | --- | --- | --- | --- | --- | --- | --- | --- | --- | --- | --- | --- | --- | --- | --- | --- | --- | --- | --- | --- | --- | --- | --- | --- | --- | --- | --- | --- | --- | --- | --- | --- | --- | --- | --- | --- | --- | --- | --- | --- | --- | --- | --- | --- | --- | --- | --- | --- | --- | --- | --- | --- | --- | --- | --- | --- | --- | --- | --- | --- | --- | --- | --- | --- | --- | --- | --- | --- | --- | --- | --- | --- | --- | --- | --- | --- | --- | --- | --- | --- | --- | --- | --- | --- | --- | --- | --- | --- | --- | --- | --- | --- | --- | --- | --- | --- | --- | --- | --- | --- | --- | --- | --- | --- | --- | --- | --- | --- | --- | --- | --- | --- | --- | --- | --- | --- | --- | --- | --- | --- | --- | --- | --- | --- | --- | --- | --- | --- | --- | --- | --- | --- | --- | --- | --- | --- | --- | --- | --- | --- | --- | --- | --- | --- | --- | --- | --- | --- | --- | --- | --- | --- | --- | --- | --- | --- | --- | --- | --- | --- | --- | --- | --- | --- | --- | --- | --- | --- | --- | --- | --- | --- | --- | --- | --- | --- | --- | --- | --- | --- | --- | --- | --- | --- | --- | --- | --- | --- | --- | --- | --- | --- | --- | --- | --- | --- | --- | --- | --- | --- | --- | --- | --- | --- | --- | --- | --- | --- | --- | --- | --- | --- | --- | --- | --- | --- | --- | --- | --- | --- | --- | --- | --- | --- | --- | --- | --- | --- | --- | --- | --- | --- | --- | --- | --- | --- | --- | --- | --- | --- | --- | --- | --- | --- | --- | --- | --- | --- | --- | --- | --- | --- | --- | --- | --- | --- | --- | --- | --- | --- | --- | --- | --- | --- | --- | --- | --- | --- | --- | --- | --- | --- | --- | --- | --- | --- | --- | --- | --- | --- | --- | --- | --- | --- | --- | --- | --- | --- | --- | --- | --- | --- | --- | --- | --- | --- | --- | --- | --- | --- | --- | --- | --- | --- | --- | --- | --- | --- | --- | --- | --- | --- | --- | --- | --- | --- | --- | --- | --- | --- | --- | --- | --- | --- | --- | --- | --- | --- | --- | --- | --- | --- | --- | --- | --- | --- | --- | --- | --- | --- | --- | --- | --- | --- | --- | --- | --- | --- | --- | --- | --- | --- | --- | --- | --- | --- | --- | --- | --- | --- | --- | --- | --- | --- | --- | --- | --- | --- | --- | --- | --- | --- | --- | --- | --- | --- | --- | --- | --- | --- | --- | --- | --- | --- | --- | --- | --- | --- | --- | --- | --- | --- | --- | --- | --- | --- | --- | --- | --- | --- | --- | --- | --- | --- | --- | --- | --- | --- | --- | --- | --- | --- | --- | --- | --- | --- | --- | --- | --- | --- | --- | --- | --- | --- | --- | --- | --- | --- | --- | --- | --- | --- | --- | --- | --- | --- | --- | --- | --- | --- | --- | --- | --- | --- | --- | --- | --- | --- | --- | --- | --- | --- | --- | --- | --- | --- | --- | --- | --- | --- | --- | --- | --- | --- | --- | --- | --- | --- | --- | --- | --- | --- | --- | --- | --- | --- | --- | --- | --- | --- | --- | --- | --- | --- | --- | --- | --- | --- | --- | --- | --- | --- | --- | --- | --- | --- | --- | --- | --- | --- | --- | --- | --- | --- | --- | --- | --- | --- | --- | --- | --- | --- | --- | --- | --- | --- | --- | --- | --- | --- | --- | --- | --- | --- | --- | --- | --- | --- | --- | --- | --- | --- | --- | --- | --- | --- | --- | --- | --- | --- | --- | --- | --- | --- | --- | --- | --- | --- | --- | --- | --- | --- | --- | --- | --- | --- | --- | --- | --- | --- | --- | --- | --- | --- | --- | --- | --- | --- | --- | --- | --- | --- | --- | --- | --- | --- | --- | --- | --- | --- | --- | --- | --- | --- | --- | --- | --- | --- | --- | --- | --- | --- | --- | --- | --- | --- | --- | --- | --- | --- | --- | --- | --- | --- | --- | --- | --- | --- | --- | --- | --- | --- | --- | --- | --- | --- | --- | --- | --- | --- | --- | --- | --- | --- | --- | --- | --- | --- | --- | --- | --- | --- | --- | --- | --- | --- | --- | --- | --- | --- | --- | --- | --- | --- | --- | --- | --- | --- | --- | --- | --- | --- | --- | --- | --- | --- | --- | --- | --- | --- | --- | --- | --- | --- | --- | --- | --- | --- | --- | --- | --- | --- | --- | --- | --- | --- | --- | --- | --- | --- | --- | --- | --- | --- | --- | --- | --- | --- | --- | --- | --- | --- | --- | --- | --- | --- | --- | --- | --- | --- | --- | --- | --- | --- | --- | --- | --- | --- | --- | --- | --- | --- | --- | --- | --- | --- | --- | --- | --- | --- | --- | --- | --- | --- | --- | --- | --- | --- | --- | --- | --- | --- | --- | --- | --- | --- | --- | --- | --- | --- | --- | --- | --- | --- | --- | --- | --- | --- | --- | --- | --- | --- | --- | --- | --- | --- | --- | --- | --- | --- | --- | --- | --- | --- | --- | --- | --- | --- | --- | --- | --- | --- | --- | --- | --- | --- | --- | --- | --- | --- | --- | --- | --- | --- | --- | --- | --- | --- | --- | --- | --- | --- | --- | --- | --- | --- | --- | --- | --- | --- | --- | --- | --- | --- | --- | --- | --- | --- | --- | --- | --- | --- | --- | --- | --- | --- | --- | --- | --- | --- | --- | --- | --- | --- | --- | --- | --- | --- | --- | --- | --- | --- | --- | --- | --- | --- | --- | --- | --- | --- | --- | --- | --- | --- | --- | --- | --- | --- | --- | --- | --- | --- | --- | --- | --- | --- | --- | --- | --- | --- | --- | --- | --- | --- | --- | --- | --- | --- | --- | --- | --- | --- | --- | --- | --- | --- | --- | --- | --- | --- | --- | --- | --- | --- | --- | --- | --- | --- | --- | --- | --- | --- | --- | --- | --- | --- | --- | --- | --- | --- | --- | --- | --- | --- | --- | --- | --- | --- | --- | --- | --- | --- | --- | --- | --- | --- | --- | --- | --- | --- | --- | --- | --- | --- | --- | --- | --- | --- | --- | --- | --- | --- | --- | --- | --- | --- | --- | --- | --- | --- | --- | --- | --- | --- | --- | --- | --- | --- | --- | --- | --- | --- | --- | --- | --- | --- | --- | --- | --- | --- | --- | --- | --- | --- | --- | --- | --- | --- | --- | --- | --- | --- | --- | --- | --- | --- | --- | --- | --- | --- | --- | --- | --- | --- | --- | --- | --- | --- | --- | --- | --- | --- | --- | --- | --- | --- | --- | --- | --- | --- | --- | --- | --- | --- | --- | --- | --- | --- | --- | --- | --- | --- | --- | --- | --- | --- | --- | --- | --- | --- | --- | --- | --- | --- | --- | --- | --- | --- | --- | --- | --- | --- | --- | --- | --- | --- | --- | --- | --- | --- | --- | --- | --- | --- | --- | --- | --- | --- | --- | --- | --- | --- | --- | --- | --- | --- | --- | --- | --- | --- | --- | --- | --- | --- | --- | --- | --- | --- | --- | --- | --- | --- | --- | --- | --- | --- | --- | --- | --- | --- | --- | --- | --- | --- | --- | --- | --- | --- | --- | --- | --- | --- | --- | --- | --- | --- | --- | --- | --- | --- | --- | --- | --- | --- | --- | --- | --- | --- | --- | --- | --- | --- | --- | --- | --- | --- | --- | --- | --- | --- | --- | --- | --- | --- | --- | --- | --- | --- | --- | --- | --- | --- | --- | --- | --- | --- | --- | --- | --- | --- | --- | --- | --- | --- | --- | --- | --- | --- | --- | --- | --- | --- | --- | --- | --- | --- | --- | --- | --- | --- | --- | --- | --- | --- | --- | --- | --- | --- | --- | --- | --- | --- | --- | --- | --- | --- | --- | --- | --- | --- | --- | --- | --- | --- | --- | --- | --- | --- | --- | --- | --- | --- | --- | --- | --- | --- | --- | --- | --- | --- | --- | --- | --- | --- | --- | --- | --- | --- | --- | --- | --- | --- | --- | --- | --- | --- | --- | --- | --- | --- | --- | --- | --- | --- | --- | --- | --- | --- | --- | --- | --- | --- | --- | --- | --- | --- | --- | --- | --- | --- | --- | --- | --- | --- | --- | --- | --- | --- | --- | --- | --- | --- | --- | --- | --- | --- | --- | --- | --- | --- | --- | --- | --- | --- | --- | --- | --- | --- | --- | --- | --- | --- | --- | --- | --- | --- | --- | --- | --- | --- | --- | --- | --- | --- | --- | --- | --- | --- | --- | --- | --- | --- | --- | --- | --- | --- | --- | --- | --- | --- | --- | --- | --- | --- | --- | --- | --- | --- | --- | --- | --- | --- | --- | --- | --- | --- | --- | --- | --- | --- | --- | --- | --- | --- | --- | --- | --- | --- | --- | --- | --- | --- | --- | --- | --- | --- | --- | --- | --- | --- | --- | --- | --- | --- | --- | --- | --- | --- | --- | --- | --- | --- | --- | --- | --- | --- | --- | --- | --- | --- | --- | --- | --- | --- | --- | --- | --- | --- | --- | --- | --- | --- | --- | --- | --- | --- | --- | --- | --- | --- | --- | --- | --- | --- | --- | --- | --- | --- | --- | --- | --- | --- | --- | --- | --- | --- | --- | --- | --- | --- | --- | --- | --- | --- | --- | --- | --- | --- | --- | --- | --- | --- | --- | --- | --- | --- | --- | --- | --- | --- | --- | --- | --- | --- | --- | --- | --- | --- | --- | --- | --- | --- | --- | --- | --- | --- | --- | --- | --- | --- | --- | --- | --- | --- | --- | --- | --- | --- | --- | --- | --- | --- | --- | --- | --- | --- | --- | --- | --- | --- | --- | --- | --- | --- | --- | --- | --- | --- | --- | --- | --- | --- | --- | --- | --- | --- | --- | --- | --- | --- | --- | --- | --- | --- | --- | --- | --- | --- | --- | --- | --- | --- | --- | --- | --- | --- | --- | --- | --- | --- | --- | --- | --- | --- | --- | --- | --- | --- | --- | --- | --- | --- | --- | --- | --- | --- | --- | --- | --- | --- | --- | --- | --- | --- | --- | --- | --- | --- | --- | --- | --- | --- | --- | --- | --- | --- | --- | --- | --- | --- | --- | --- | --- | --- | --- | --- | --- | --- | --- | --- | --- | --- | --- | --- | --- | --- | --- | --- | --- | --- | --- | --- | --- | --- | --- | --- | --- | --- | --- | --- | --- | --- | --- | --- | --- | --- | --- | --- | --- | --- | --- | --- | --- | --- | --- | --- | --- | --- | --- | --- | --- | --- | --- | --- | --- | --- | --- | --- | --- | --- | --- | --- | --- | --- | --- | --- | --- | --- | --- | --- | --- | --- | --- | --- | --- | --- | --- | --- | --- | --- | --- | --- | --- | --- | --- | --- | --- | --- | --- | --- | --- | --- | --- | --- | --- | --- | --- | --- | --- | --- | --- | --- | --- | --- | --- | --- | --- | --- | --- | --- | --- | --- | --- | --- | --- | --- | --- | --- | --- | --- | --- | --- | --- | --- | --- | --- | --- | --- | --- | --- | --- | --- | --- | --- | --- | --- | --- | --- | --- | --- | --- | --- | --- | --- | --- | --- | --- | --- | --- | --- | --- | --- | --- | --- | --- | --- | --- | --- | --- | --- | --- | --- | --- | --- | --- | --- | --- | --- | --- | --- | --- | --- | --- | --- | --- | --- | --- | --- | --- | --- | --- | --- | --- | --- | --- | --- | --- | --- | --- | --- | --- | --- | --- | --- | --- | --- | --- | --- | --- | --- | --- | --- | --- | --- | --- | --- | --- | --- | --- | --- | --- | --- | --- | --- | --- | --- | --- | --- | --- | --- | --- | --- | --- | --- | --- | --- | --- | --- | --- | --- | --- | --- | --- | --- | --- | --- | --- | --- | --- | --- | --- | --- | --- | --- | --- | --- | --- | --- | --- | --- | --- | --- | --- | --- | --- | --- | --- | --- | --- | --- | --- | --- | --- | --- | --- | --- | --- | --- | --- | --- | --- | --- | --- | --- | --- | --- | --- | --- | --- | --- | --- | --- | --- | --- | --- | --- | --- | --- | --- | --- | --- | --- | --- | --- | --- | --- | --- | --- | --- | --- | --- | --- | --- | --- | --- | --- | --- | --- | --- | --- | --- | --- | --- | --- | --- | --- | --- | --- | --- | --- | --- | --- | --- | --- | --- | --- | --- | --- | --- | --- | --- | --- | --- | --- | --- | --- | --- | --- | --- | --- | --- | --- | --- | --- | --- | --- | --- | --- | --- | --- | --- | --- | --- | --- | --- | --- | --- | --- | --- | --- | --- | --- | --- | --- | --- | --- | --- | --- | --- | --- | --- | --- | --- | --- | --- | --- | --- | --- | --- | --- | --- | --- | --- | --- | --- | --- | --- | --- | --- | --- | --- | --- | --- | --- | --- | --- | --- | --- | --- | --- | --- | --- | --- | --- | --- | --- | --- | --- | --- | --- | --- | --- | --- | --- | --- | --- | --- | --- | --- | --- | --- | --- | --- | --- | --- | --- | --- | --- | --- | --- | --- | --- | --- | --- | --- | --- | --- | --- | --- | --- | --- | --- | --- | --- | --- | --- | --- | --- | --- | --- | --- | --- | --- | --- | --- | --- | --- | --- | --- | --- | --- | --- | --- | --- | --- | --- | --- | --- | --- | --- | --- | --- | --- | --- | --- | --- | --- | --- | --- | --- | --- | --- | --- | --- | --- | --- | --- | --- | --- | --- | --- | --- | --- | --- | --- | --- | --- | --- | --- | --- | --- | --- | --- | --- | --- | --- | --- | --- | --- | --- | --- | --- | --- | --- | --- | --- | --- | --- | --- | --- | --- | --- | --- | --- | --- | --- | --- | --- | --- | --- | --- | --- | --- | --- | --- | --- | --- | --- | --- | --- | --- | --- | --- | --- | --- | --- | --- | --- | --- | --- | --- | --- | --- | --- | --- | --- | --- | --- | --- | --- | --- | --- | --- | --- | --- | --- | --- | --- | --- | --- | --- | --- | --- | --- | --- | --- | --- | --- | --- | --- | --- | --- | --- | --- | --- | --- | --- | --- | --- | --- | --- | --- | --- | --- | --- | --- | --- | --- | --- | --- | --- | --- | --- | --- | --- | --- | --- | --- | --- | --- | --- | --- | --- | --- | --- | --- | --- | --- | --- | --- | --- | --- | --- | --- | --- | --- | --- | --- | --- | --- | --- | --- | --- | --- | --- | --- | --- | --- | --- | --- | --- | --- | --- | --- | --- | --- | --- | --- | --- | --- | --- | --- | --- | --- | --- | --- | --- | --- | --- | --- | --- | --- | --- | --- | --- | --- | --- | --- | --- | --- | --- | --- | --- | --- | --- | --- | --- | --- | --- | --- | --- | --- | --- | --- | --- | --- | --- | --- | --- | --- | --- | --- | --- | --- | --- | --- | --- | --- | --- | --- | --- | --- | --- | --- | --- | --- | --- | --- | --- | --- | --- | --- | --- | --- | --- | --- | --- | --- | --- | --- | --- | --- | --- | --- | --- | --- | --- | --- | --- | --- | --- | --- | --- | --- | --- | --- | --- | --- | --- | --- | --- | --- | --- | --- | --- | --- | --- | --- | --- | --- | --- | --- | --- | --- | --- | --- | --- | --- | --- | --- | --- | --- | --- | --- | --- | --- | --- | --- | --- | --- | --- | --- | --- | --- | --- | --- | --- | --- | --- | --- | --- | --- | --- | --- | --- | --- | --- | --- | --- | --- | --- | --- | --- | --- | --- | --- | --- | --- | --- | --- | --- | --- | --- | --- | --- | --- | --- | --- | --- | --- | --- | --- | --- | --- | --- | --- | --- | --- | --- | --- | --- | --- | --- | --- | --- | --- | --- | --- | --- | --- | --- | --- | --- | --- | --- | --- | --- | --- | --- | --- | --- | --- | --- | --- | --- | --- | --- | --- | --- | --- | --- | --- | --- | --- | --- | --- | --- | --- | --- | --- | --- | --- | --- | --- | --- | --- | --- | --- | --- | --- | --- | --- | --- | --- | --- | --- | --- | --- | --- | --- | --- | --- | --- | --- | --- | --- | --- | --- | --- | --- | --- | --- | --- | --- | --- | --- | --- | --- | --- | --- | --- | --- | --- | --- | --- | --- | --- | --- | --- | --- | --- | --- | --- | --- | --- | --- | --- | --- | --- | --- | --- | --- | --- | --- | --- | --- | --- | --- | --- | --- | --- | --- | --- | --- | --- | --- | --- | --- | --- | --- | --- | --- | --- | --- | --- | --- | --- | --- | --- | --- | --- | --- | --- | --- | --- | --- | --- | --- | --- | --- | --- | --- | --- | --- | --- | --- | --- | --- | --- | --- | --- | --- | --- | --- | --- | --- | --- | --- | --- | --- | --- | --- | --- | --- | --- | --- | --- | --- | --- | --- | --- | --- | --- | --- | --- | --- | --- | --- | --- | --- | --- | --- | --- | --- | --- | --- | --- | --- | --- | --- | --- | --- | --- | --- | --- | --- | --- | --- | --- | --- | --- | --- | --- | --- | --- | --- | --- | --- | --- | --- | --- | --- | --- | --- | --- | --- | --- | --- | --- | --- | --- | --- | --- | --- | --- | --- | --- | --- | --- | --- | --- | --- | --- | --- | --- | --- | --- | --- | --- | --- | --- | --- | --- | --- | --- | --- | --- | --- | --- | --- | --- | --- | --- | --- | --- | --- | --- | --- | --- | --- | --- | --- | --- | --- | --- | --- | --- | --- | --- | --- | --- | --- | --- | --- | --- | --- | --- | --- | --- | --- | --- | --- | --- | --- | --- | --- | --- | --- | --- | --- | --- | --- | --- | --- | --- | --- | --- | --- | --- | --- | --- | --- | --- | --- | --- | --- | --- | --- | --- | --- | --- | --- | --- | --- | --- | --- | --- | --- | --- | --- | --- | --- | --- | --- | --- | --- | --- | --- | --- | --- | --- | --- | --- | --- | --- | --- | --- | --- | --- | --- | --- | --- | --- | --- | --- | --- | --- | --- | --- | --- | --- | --- | --- | --- | --- | --- | --- | --- | --- | --- | --- | --- | --- | --- | --- | --- | --- | --- | --- | --- | --- | --- | --- | --- | --- | --- | --- | --- | --- | --- | --- | --- | --- | --- | --- | --- | --- | --- | --- | --- | --- | --- | --- | --- | --- | --- | --- | --- | --- | --- | --- | --- | --- | --- | --- | --- | --- | --- | --- | --- | --- | --- | --- | --- | --- | --- | --- | --- | --- | --- | --- | --- | --- | --- | --- | --- | --- | --- | --- | --- | --- | --- | --- | --- | --- | --- | --- | --- | --- | --- | --- | --- | --- | --- | --- | --- | --- | --- | --- | --- | --- | --- | --- | --- | --- | --- | --- | --- | --- | --- | --- | --- | --- | --- | --- | --- | --- | --- | --- | --- | --- | --- | --- | --- | --- | --- | --- | --- | --- | --- | --- | --- | --- | --- | --- | --- | --- | --- | --- | --- | --- | --- | --- | --- | --- | --- | --- | --- | --- | --- | --- | --- | --- | --- | --- | --- | --- | --- | --- | --- | --- | --- | --- | --- | --- | --- | --- | --- | --- | --- | --- | --- | --- | --- | --- | --- | --- | --- | --- | --- | --- | --- | --- | --- | --- | --- | --- | --- | --- | --- | --- | --- | --- | --- | --- | --- | --- | --- | --- | --- | --- | --- | --- | --- | --- | --- | --- | --- | --- | --- | --- | --- | --- | --- | --- | --- | --- | --- | --- | --- | --- | --- | --- | --- | --- | --- | --- | --- | --- | --- | --- | --- | --- | --- | --- | --- | --- | --- | --- | --- | --- | --- | --- | --- | --- | --- | --- | --- | --- | --- | --- | --- | --- | --- | --- | --- | --- | --- | --- | --- | --- | --- | --- | --- | --- | --- | --- | --- | --- | --- | --- | --- | --- | --- | --- | --- | --- | --- | --- | --- | --- | --- | --- | --- | --- | --- | --- | --- | --- | --- | --- | --- | --- | --- | --- | --- | --- | --- | --- | --- | --- | --- | --- | --- | --- | --- | --- | --- | --- | --- | --- | --- | --- | --- | --- | --- | --- | --- | --- | --- | --- | --- | --- | --- | --- | --- | --- | --- | --- | --- | --- | --- | --- | --- | --- | --- | --- | --- | --- | --- | --- | --- | --- | --- | --- | --- | --- | --- | --- | --- | --- | --- | --- | --- | --- | --- | --- | --- | --- | --- | --- | --- | --- | --- | --- | --- | --- | --- | --- | --- | --- | --- | --- | --- | --- | --- | --- | --- | --- | --- | --- | --- | --- | --- | --- | --- | --- | --- | --- | --- | --- | --- | --- | --- | --- | --- | --- | --- | --- | --- | --- | --- | --- | --- | --- | --- | --- | --- | --- | --- | --- | --- | --- | --- | --- | --- | --- | --- | --- | --- | --- | --- | --- | --- | --- | --- | --- | --- | --- | --- | --- | --- | --- | --- | --- | --- | --- | --- | --- | --- | --- | --- | --- | --- | --- | --- | --- | --- | --- | --- | --- | --- | --- | --- | --- | --- | --- | --- | --- | --- | --- | --- | --- | --- | --- | --- | --- | --- | --- | --- | --- | --- | --- | --- | --- | --- | --- | --- | --- | --- | --- | --- | --- | --- | --- | --- | --- | --- | --- | --- | --- | --- | --- | --- | --- | --- | --- | --- | --- | --- | --- | --- | --- | --- | --- | --- | --- | --- | --- | --- | --- | --- | --- | --- | --- | --- | --- | --- | --- | --- | --- | --- | --- | --- | --- | --- | --- | --- | --- | --- | --- | --- | --- | --- | --- | --- | --- | --- | --- | --- | --- | --- | --- | --- | --- | --- | --- | --- | --- | --- | --- | --- | --- | --- | --- | --- | --- | --- | --- | --- | --- | --- | --- | --- | --- | --- | --- | --- | --- | --- | --- | --- | --- | --- | --- | --- | --- | --- | --- | --- | --- | --- | --- | --- | --- | --- | --- | --- | --- | --- | --- | --- | --- | --- | --- | --- | --- | --- | --- | --- | --- | --- | --- | --- | --- | --- | --- | --- | --- | --- | --- | --- | --- | --- | --- | --- | --- | --- | --- | --- | --- | --- | --- | --- | --- | --- | --- | --- | --- | --- | --- | --- | --- | --- | --- | --- | --- | --- | --- | --- | --- | --- | --- | --- | --- | --- | --- | --- | --- | --- | --- | --- | --- | --- | --- | --- | --- | --- | --- | --- | --- | --- | --- | --- | --- | --- | --- | --- | --- | --- | --- | --- | --- | --- | --- | --- | --- | --- | --- | --- | --- | --- | --- | --- | --- | --- | --- | --- | --- | --- | --- | --- | --- | --- | --- | --- | --- | --- | --- | --- | --- | --- | --- | --- | --- | --- | --- | --- | --- | --- | --- | --- | --- | --- | --- | --- | --- | --- | --- | --- | --- | --- | --- | --- | --- | --- | --- | --- | --- | --- | --- | --- | --- | --- | --- | --- | --- | --- | --- | --- | --- | --- | --- | --- | --- | --- | --- | --- | --- | --- | --- | --- | --- | --- | --- | --- | --- | --- | --- | --- | --- | --- | --- | --- | --- | --- | --- | --- | --- | --- | --- | --- | --- | --- | --- | --- | --- | --- | --- | --- | --- | --- | --- | --- | --- | --- | --- | --- | --- | --- | --- | --- | --- | --- | --- | --- | --- | --- | --- | --- | --- | --- | --- | --- | --- | --- | --- | --- | --- | --- | --- | --- | --- | --- | --- | --- | --- | --- | --- | --- | --- | --- | --- | --- | --- | --- | --- | --- | --- | --- | --- | --- | --- | --- | --- | --- | --- | --- | --- | --- | --- | --- | --- | --- | --- | --- | --- | --- | --- | --- | --- | --- | --- | --- | --- | --- | --- | --- | --- | --- | --- | --- | --- | --- | --- | --- | --- | --- | --- | --- | --- | --- | --- | --- | --- | --- | --- | --- | --- | --- | --- | --- | --- | --- | --- | --- | --- | --- | --- | --- | --- | --- | --- | --- | --- | --- | --- | --- | --- | --- | --- | --- | --- | --- | --- | --- | --- | --- | --- | --- | --- | --- | --- | --- | --- | --- | --- | --- | --- | --- | --- | --- | --- | --- | --- | --- | --- | --- | --- | --- | --- | --- | --- | --- | --- | --- | --- | --- | --- | --- | --- | --- | --- | --- | --- | --- | --- | --- | --- | --- | --- | --- | --- | --- | --- | --- | --- | --- | --- | --- | --- | --- | --- | --- | --- | --- | --- | --- | --- | --- | --- | --- | --- | --- | --- | --- | --- | --- | --- | --- | --- | --- | --- | --- | --- | --- | --- | --- | --- | --- | --- | --- | --- | --- | --- | --- | --- | --- | --- | --- | --- | --- | --- | --- | --- | --- | --- | --- | --- | --- | --- | --- | --- | --- | --- | --- | --- | --- | --- | --- | --- | --- | --- | --- | --- | --- | --- | --- | --- | --- | --- | --- | --- | --- | --- | --- | --- | --- | --- | --- | --- | --- | --- | --- | --- | --- | --- | --- | --- | --- | --- | --- | --- | --- | --- | --- | --- | --- | --- | --- | --- | --- | --- | --- | --- | --- | --- | --- | --- | --- | --- | --- | --- | --- | --- | --- | --- | --- | --- | --- | --- | --- | --- | --- | --- | --- | --- | --- | --- | --- | --- | --- | --- | --- | --- | --- | --- | --- | --- | --- | --- | --- | --- | --- | --- | --- | --- | --- | --- | --- | --- | --- | --- | --- | --- | --- | --- | --- | --- | --- | --- | --- | --- | --- | --- | --- | --- | --- | --- | --- | --- | --- | --- | --- | --- | --- | --- | --- | --- | --- | --- | --- | --- | --- | --- | --- | --- | --- | --- | --- | --- | --- | --- | --- | --- | --- | --- | --- | --- | --- | --- | --- | --- | --- | --- | --- | --- | --- | --- | --- | --- | --- | --- | --- | --- | --- | --- | --- | --- | --- | --- | --- | --- | --- | --- | --- | --- | --- | --- | --- | --- | --- | --- | --- | --- | --- | --- | --- | --- | --- | --- | --- | --- | --- | --- | --- | --- | --- | --- | --- | --- | --- | --- | --- | --- | --- | --- | --- | --- | --- | --- | --- | --- | --- | --- | --- | --- | --- | --- | --- | --- | --- | --- | --- | --- | --- | --- | --- | --- | --- | --- | --- | --- | --- | --- | --- | --- | --- | --- | --- | --- | --- | --- | --- | --- | --- | --- | --- | --- | --- | --- | --- | --- | --- | --- | --- | --- | --- | --- | --- | --- | --- | --- | --- | --- | --- | --- | --- | --- | --- | --- | --- | --- | --- | --- | --- | --- | --- | --- | --- | --- | --- | --- | --- | --- | --- | --- | --- | --- | --- | --- | --- | --- | --- | --- | --- | --- | --- | --- | --- | --- | --- | --- | --- | --- | --- | --- | --- | --- | --- | --- | --- | --- | --- | --- | --- | --- | --- | --- | --- | --- | --- | --- | --- | --- | --- | --- | --- | --- | --- | --- | --- | --- | --- | --- | --- | --- | --- | --- | --- | --- | --- | --- | --- | --- | --- | --- | --- | --- | --- | --- | --- | --- | --- | --- | --- | --- | --- | --- | --- | --- | --- | --- | --- | --- | --- | --- | --- | --- | --- | --- | --- | --- | --- | --- | --- | --- | --- | --- | --- | --- | --- | --- | --- | --- | --- | --- | --- | --- | --- | --- | --- | --- | --- | --- | --- | --- | --- | --- | --- | --- | --- | --- | --- | --- | --- | --- | --- | --- | --- | --- | --- | --- | --- | --- | --- | --- | --- | --- | --- | --- | --- | --- | --- | --- | --- | --- | --- | --- | --- | --- | --- |

| **Table S2**: Sequences generated in our study. Samples were acquired from various sources and in different stages of processing: field collections, the Kew Living Collections, the Kew DNA bank, the Millennium Seed Bank (MSB), as well as herbarium specimens from the National Museum of Wales. Field collections were collected performed over the past few years, but Living Collections samples were collected in the summer of 2013. For field and Kew Living Collection specimens, small amounts of leaf material were used for extraction. Seeds were germinated by the MSB under controlled conditions until sufficient leaf and shoot material could be collected. As far as possible, across all specimen sources, DNA extracts and specimens of British and natural origin were acquired. BF numbers are internal unique identifiers. Accession numbers are either voucher accessions (for herbarium, plant material, seed) or Kew DNA bank IDs. Genbank accession numbers will follow when sequences have been submitted. | | | | | | |
| --- | --- | --- | --- | --- | --- | --- |
|  | Taxon.name | Source | rbcL_Genbank_accession | matK_Genbank_accession | Voucher.accession | VS.Number |
| 1 | Carex laevigata | Kew silica samples | | KF997450 | MJC246 | VS7400 |
| 2 | Ribes uva-crispa | Kew silica samples | KF997392 | KF997446 | MJC248 | VS7401 |
| 3 | Lysimachia punctata | Kew silica samples | KF997269 |  | MJC426 | VS7413 |
| 4 | Rhododendron ponticum | Museum of Wales herbarium samples | KF997508 |  | NMW8263 | VS6165 |
| 5 | Trifolium hybridum | Museum of Wales herbarium samples | KF997421 |  | NMW8282 | VS6173 |
| 6 | Impatiens glandulifera | Museum of Wales herbarium samples | KF997407 |  | NMW8286 | VS6175 |
| 7 | Galanthus nivalis | Museum of Wales herbarium samples | KF997373 |  | NMW8291 | VS6177 |
| 8 | Linaria purpurea | Museum of Wales herbarium samples | KF997324 |  | NMW8316 | VS6189 |
| 9 | Cerastium tomentosum | Museum of Wales herbarium samples | KF997331 |  | NMW8327 | VS6195 |
| 10 | Pseudofumaria lutea | Museum of Wales herbarium samples | KF997438 |  | NMW8329 | VS6196 |
| 11 | Geranium pyrenaicum | Museum of Wales herbarium samples | KF997498 |  | NMW8332 | VS6197 |
| 12 | Hyacinthoides hispanica | Museum of Wales herbarium samples | KF997302 |  | NMW8369 | VS6214 |
| 13 | Aster novi-belgii | Museum of Wales herbarium samples | KF997334 |  | NMW8374 | VS6216 |
| 14 | Myrrhis odorata | Museum of Wales herbarium samples | KF997385 |  | NMW8389 | VS6222 |
| 15 | Claytonia sibirica | Museum of Wales herbarium samples | KF997318 |  | NMW8392 | VS6223 |
| 16 | Mimulus guttatus | Museum of Wales herbarium samples | KF997284 |  | NMW8417 | VS6232 |
| 17 | Phalaris canariensis | Museum of Wales herbarium samples | KF997405 |  | NMW8426 | VS6235 |
| 18 | Oxalis exilis | Dried silwood vouchers | KF997462 |  | BF15 | VS6749 |
| 19 | Soleirolia soleirolii | Museum of Wales herbarium samples | KF997497 |  | NMW8429 | VS6236 |
| 20 | Sorbus intermedia | Museum of Wales herbarium samples | KF997366 |  | NMW8446 | VS6243 |
| 21 | Barbarea intermedia | Museum of Wales herbarium samples | KF997349 |  | NMW8462 | VS6249 |
| 22 | Diplotaxis muralis | Museum of Wales herbarium samples | KF997285 |  | NMW8467 | VS6251 |
| 23 | Sisymbrium orientale | Museum of Wales herbarium samples | KF997449 |  | NMW8490 | VS6260 |
| 24 | Crassula helmsii | Museum of Wales herbarium samples | KF997481 |  | NMW8503 | VS6265 |
| 25 | Hypericum calycinum | Museum of Wales herbarium samples | KF997379 |  | NMW8508 | VS6267 |
| 26 | Calystegia pulchra | Museum of Wales herbarium samples | KF997266 |  | NMW8526 | VS6274 |
| 27 | Geranium endressii | Museum of Wales herbarium samples | KF997472 |  | NMW8558 | VS6286 |
| 28 | Campanula portenschlagiana | Museum of Wales herbarium samples | KF997316 |  | NMW8583 | VS6297 |
| 29 | Hirschfeldia incana | Museum of Wales herbarium samples | KF997351 |  | NMW8600 | VS6305 |
| 30 | Geranium phaeum | Museum of Wales herbarium samples | KF997336 |  | NMW8609 | VS6308 |
| 31 | Symphytum orientale | Museum of Wales herbarium samples | KF997359 |  | NMW8612 | VS6309 |
| 32 | Allium paradoxum | Museum of Wales herbarium samples | KF997330 |  | NMW8691 | VS6339 |
| 33 | Amsinckia micrantha | Museum of Wales herbarium samples | KF997304 |  | NMW8696 | VS6341 |
| 34 | Erinus alpinus | Museum of Wales herbarium samples | KF997404 |  | NMW8716 | VS6348 |
| 35 | Galega officinalis | Museum of Wales herbarium samples | KF997369 |  | NMW8718 | VS6349 |
| 36 | Allium roseum | Museum of Wales herbarium samples | KF997289 |  | NMW8959 | VS6445 |
| 37 | Mimulus luteus | Museum of Wales herbarium samples | KF997256 |  | NMW9054 | VS6481 |
| 38 | Scrophularia vernalis | Museum of Wales herbarium samples | KF997389 |  | NMW9077 | VS6492 |
| 39 | Saxifraga hirsuta | Kew DNA bank | KF997488 |  | 819 | VS7417 |
| 40 | Limonium bellidifolium | Kew DNA bank | KF997474 |  | 1482 | VS7418 |
| 41 | Leucojum vernum | Kew DNA bank | KF997317 |  | 1651 | VS7419 |
| 42 | Ribes alpinum | Kew DNA bank | KF997397 |  | 3587 | VS7422 |
| 43 | Ribes spicatum | Kew DNA bank | KF997408 |  | 3609 | VS7423 |
| 44 | Lilium martagon | Kew DNA bank | KF997467 |  | 3698 | VS7424 |
| 45 | Dactylorhiza lapponica | Kew DNA bank | KF997281 |  | 5552 | VS7425 |
| 46 | Thymus serpyllum | Kew DNA bank | KF997486 |  | 7725 | VS7429 |
| 47 | Rubus spectabilis | Kew DNA bank | KF997292 |  | 8287 | VS7431 |
| 48 | Veronica verna | Kew DNA bank | KF997427 |  | 9113 | VS7433 |
| 49 | Veronica alpina | Kew DNA bank | KF997333 |  | 9137 | VS7435 |
| 50 | Erica vagans | Kew DNA bank | KF997402 |  | 10520.1 | VS7437 |
| 51 | Polemonium caeruleum | Kew DNA bank | KF997311 |  | 11084 | VS7440 |
| 52 | Lavatera arborea | Kew DNA bank | KF997314 |  | 11405 | VS7442 |
| 53 | Circaea lutetiana | Kew DNA bank | | KF997503 | 11524 | VS7444 |
| 54 | Euphrasia officinalis | Kew DNA bank | KF997364 |  | 11532 | VS7445 |
| 55 | Monotropa hypopithys | Kew DNA bank | KF997344 |  | 11554 | VS7446 |
| 56 | Epilobium anagallidifolium | Kew DNA bank | KF997410 |  | 11880 | VS7448 |
| 57 | Pilularia globulifera | Kew DNA bank | | KF997478 | 11997 | VS7449 |
| 58 | Orchis militaris | Kew DNA bank | KF997273 | KF997352 | 12137 | VS7452 |
| 59 | Cicerbita macrophylla | Kew DNA bank | KF997257 |  | 12942 | VS7456 |
| 60 | Colutea arborescens | Kew DNA bank | KF997293 |  | 12943 | VS7457 |
| 61 | Oxytropis campestris | Kew DNA bank | KF997460 |  | 12965 | VS7459 |
| 62 | Gladiolus communis | Kew DNA bank | KF997464 |  | 13702 | VS7462 |
| 63 | Subularia aquatica | Kew DNA bank | KF997270 |  | 14902 | VS7469 |
| 64 | Anacamptis morio | Kew DNA bank | KF997322 | KF997506 | 16368 | VS7470 |
| 65 | Alchemilla micans | Kew DNA bank | KF997260 | KF997394 | 17217 | VS7471 |
| 66 | Potentilla argentea | Kew DNA bank | KF997452 |  | 17218 | VS7472 |
| 67 | Alchemilla mollis | Kew DNA bank | | KF997444 | 17224 | VS7473 |
| 68 | Petasites albus | Kew DNA bank | KF997274 |  | 17722 | VS7474 |
| 69 | Goodyera repens | Kew DNA bank | | KF997278 | 17773 | VS7475 |
| 70 | Epipactis purpurata | Kew DNA bank | | KF997339 | 17794 | VS7476 |
| 71 | Phleum alpinum | Kew DNA bank | KF997288 | KF997378 | 18904 | VS7478 |
| 72 | Epipogium aphyllum | Kew DNA bank | KF997303 |  | 19248 | VS7480 |
| 73 | Apera spica-venti | Kew DNA bank | KF997279 |  | 20166 | VS7482 |
| 74 | Isoetes histrix | Kew DNA bank | | KF997363 | 20174 | VS7483 |
| 75 | Ludwigia palustris | Kew DNA bank | | KF997300 | 20175 | VS7484 |
| 76 | Linaria pelisseriana | Kew DNA bank | KF997431 | KF997309 | 20618 | VS7485 |
| 77 | Spiranthes romanzoffiana | Kew DNA bank | | KF997301 | 20892 | VS7486 |
| 78 | Sagina saginoides | Kew DNA bank | KF997390 |  | 21002 | VS7487 |
| 79 | Trichomanes speciosum | Kew DNA bank | KF997443 | KF997430 | 22482 | VS7488 |
| 80 | Gentianella germanica | Kew DNA bank | KF997490 |  | 23085 | VS7490 |
| 81 | Helianthemum apenninum | Kew DNA bank | KF997432 |  | 23913 | VS7491 |
| 82 | Scilla autumnalis | Kew DNA bank | | KF997268 | 24815 | VS7493 |
| 83 | Allium scorodoprasum | Kew DNA bank | KF997327 | KF997391 | 26042 | VS7495 |
| 84 | Saxifraga rivularis | Kew DNA bank | KF997383 |  | 28920 | VS7496 |
| 85 | Rosa rugosa | Kew DNA bank | KF997265 |  | 32940 | VS7497 |
| 86 | Orchis purpurea | Kew DNA bank | KF997502 |  | 34529 | VS7499 |
| 87 | Anacamptis laxiflora | Kew DNA bank | KF997401 | KF997312 | 35747 | VS7500 |
| 88 | Solanum sarrachoides | Kew DNA bank | KF997419 | KF997283 | 38617 | VS7501 |
| 89 | Equisetum fluviatile | Kew DNA bank | | KF997291 | 41076 | VS7503 |
| 90 | Orchis ustulata | Kew DNA bank | KF997335 |  | O-755 | VS7505 |
| 91 | Sempervivum tectorum | RBGKew Liv. Coll. | KF997305 |  | 1969-12107 | VS7507 |
| 92 | Arenaria ciliata | RBGKew Liv. Coll. | KF997441 |  | 2002-193 | VS7509 |
| 93 | Erigeron borealis | RBGKew Liv. Coll. | KF997411 |  | 1992-549 | VS7510 |
| 94 | Ornithogalum umbellatum | RBGKew Liv. Coll. | KF997468 |  | 1959-47203 | VS7511 |
| 95 | Primula scotica | RBGKew Liv. Coll. | KF997463 |  | 1990-3087 | VS7512 |
| 96 | Erica ciliaris | RBGKew Liv. Coll. | KF997417 |  | 1994-2554 | VS7517 |
| 97 | Salix myrsinites | RBGKew Liv. Coll. | KF997500 |  | 1969-50377 | VS7518 |
| 98 | Lonicera xylosteum | RBGKew Liv. Coll. | KF997358 |  | 1997-6537 | VS7519 |
| 99 | Pulsatilla vulgaris | RBGKew Liv. Coll. | KF997425 |  | 1978-2478 | VS7520 |
| 100 | Verbascum lychnitis | RBGKew Liv. Coll. | KF997381 |  | 2000-2221 | VS7523 |
| 101 | Sorbus lancastriensis | Wakehurst Liv. Coll. | KF997487 |  | 1992-3742 | VS7524 |
| 102 | Sorbus subcuneata | Wakehurst Liv. Coll. | KF997455 |  | 1992-3744 | VS7525 |
| 103 | Sorbus vexans | Wakehurst Liv. Coll. | KF997461 |  | 1996-5188 | VS7526 |
| 104 | Althaea hirsuta | Wakehurst Liv. Coll. | KF997457 |  | 2002-2915 | VS7529 |
| 105 | Cardamine bulbifera | Wakehurst Liv. Coll. | KF997328 |  | 2011-1034 | VS7530 |
| 106 | Cotoneaster simonsii | Wakehurst Liv. Coll. | KF997277 |  | 1990-2541 | VS7534 |
| 107 | Festuca longifolia | RBGKew Liv. Coll. | KF997420 |  | 1975-2219 | VS7537 |
| 108 | Poa chaixii | RBGKew Liv. Coll. | KF997493 |  | 1969-19117 | VS7538 |
| 109 | Festuca armoricana | RBGKew Liv. Coll. | KF997423 |  | 1995-1359 | VS7539 |
| 110 | Koeleria vallesiana | RBGKew Liv. Coll. | KF997263 |  | 1991-239 | VS7540 |
| 111 | Phleum phleoides | RBGKew Liv. Coll. | KF997295 |  | 2000-1225 | VS7541 |
| 112 | Sorbus arranensis | RBGKew Liv. Coll. | KF997471 |  | 1988-6265 / 1980-6265? | VS7542 |
| 113 | Sorbus devoniensis | RBGKew Liv. Coll. | KF997276 |  | 1980-6328 | VS7543 |
| 114 | Sorbus bristoliensis | RBGKew Liv. Coll. | KF997399 |  | 1966-66114 | VS7544 |
| 115 | Sorbus pseudofennica | RBGKew Liv. Coll. | KF997433 |  | 1980-1818 | VS7545 |
| 116 | Primula elatior | RBGKew Liv. Coll. | KF997489 |  | No acc. | VS7547 |
| 117 | Cerastium brachypetalum | Kew Millennium Seed Bank | KF997372 |  | 9782 | VS8033.23 |
| 118 | Petrorhagia prolifera | Kew Millennium Seed Bank | KF997492 |  | 51574 | VS8034.23 |
| 119 | Tordylium maximum | Kew Millennium Seed Bank | KF997310 |  | 97341 | VS8035.23 |
| 120 | Phalaris minor | Kew Millennium Seed Bank | KF997414 |  | 113968 | VS8037.23 |
| 121 | Crepis vesicaria | Kew Millennium Seed Bank | KF997466 |  | 114806 | VS8038.23 |
| 122 | Cerastium nigrescens | Kew Millennium Seed Bank | KF997275 |  | 122629 | VS8039.23 |
| 123 | Limonium normannicum | Kew Millennium Seed Bank | KF997356 |  | 123279 | VS8040.23 |
| 124 | Armeria arenaria | Kew Millennium Seed Bank | KF997272 |  | 123280 | VS8041.23 |
| 125 | Rumex longifolius | Kew Millennium Seed Bank | KF997361 |  | 131483 | VS8042.23 |
| 126 | Draba muralis | Kew Millennium Seed Bank | KF997354 |  | 119384 | VS8046.23 |
| 127 | Carex vulpina | Kew Millennium Seed Bank | KF997370 |  | 120441 | VS8047.23 |
| 128 | Gnaphalium supinum | Kew Millennium Seed Bank | KF997337 |  | 121105 | VS8048.23 |
| 129 | Sonchus palustris | Kew Millennium Seed Bank | KF997470 |  | 112503 | VS8049.23 |
| 130 | Epilobium alsinifolium | Kew Millennium Seed Bank | KF997325 |  | 154408 | VS8051.23 |
| 131 | Silene otites | Kew Millennium Seed Bank | KF997306 |  | 35019 | VS8052.23 |
| 132 | Vulpia unilateralis | Kew Millennium Seed Bank | KF997332 |  | 143259 | VS8054.23 |
| 133 | Trifolium bocconei | Kew Millennium Seed Bank | KF997424 |  | 111344 | VS8056.23 |
| 134 | Epilobium ciliatum | Dried silwood vouchers | | KF997264 | BF4 | VS6738 |
| 135 | Aceras anthropophorum | Kew DNA bank | KF997307 |  | O-761 | VS7328 |
| 136 | Orchis simia | Kew DNA bank | KF997259 | KF997476 | 7227 | VS7329 |
| 137 | Ligusticum scoticum | Kew DNA bank | KF997267 |  | 8278 | VS7330 |
| 138 | Viola persicifolia | Kew DNA bank | KF997501 |  | 10126 | VS7331 |
| 139 | Arbutus unedo | Kew DNA bank | KF997388 | KF997315 | 10518.1 | VS7332 |
| 140 | Impatiens parviflora | Kew DNA bank | KF997297 | KF997439 | 11068 | VS7334 |
| 141 | Peucedanum palustre | Kew DNA bank | KF997499 |  | 11079 | VS7335 |
| 142 | Pentaglottis sempervirens | Kew DNA bank | KF997280 |  | 11308 | VS7337 |
| 143 | Veronica fruticans | Kew DNA bank | KF997290 |  | 11311 | VS7338 |
| 144 | Epilobium ciliatum | Kew DNA bank | KF997326 |  | 11396 | VS7340 |
| 145 | Epilobium brunnescens | Kew DNA bank | KF997341 |  | 11636 | VS7341 |
| 146 | Calystegia silvatica | Kew DNA bank | KF997298 | KF997403 | 11987 | VS7342 |
| 147 | Alchemilla glomerulans | Kew DNA bank | KF997342 | KF997376 | 12364 | VS7343 |
| 148 | Alchemilla wichurae | Kew DNA bank | KF997374 | KF997398 | 12366 | VS7344 |
| 149 | Alchemilla monticola | Kew DNA bank | KF997494 |  | 12367 | VS7345 |
| 150 | Alchemilla acutiloba | Kew DNA bank | KF997350 | KF997313 | 12370 | VS7346 |
| 151 | Acaena novae-zelandiae | Kew DNA bank | KF997415 | KF997396 | 12925 | VS7347 |
| 152 | Saussurea alpina | Kew DNA bank | KF997375 |  | 12974 | VS7349 |
| 153 | Alchemilla alpina | Kew DNA bank | KF997346 | KF997340 | 13676 | VS7350 |
| 154 | Himantoglossum hircinum | Kew DNA bank | KF997440 | KF997261 | 14051 | VS7351 |
| 155 | Herniaria ciliolata | Kew DNA bank | KF997294 |  | 14512 | VS7352 |
| 156 | Trinia glauca | Kew DNA bank | KF997473 | KF997485 | 14522 | VS7353 |
| 157 | Phyteuma orbiculare | Kew DNA bank | KF997454 |  | 14632 | VS7354 |
| 158 | Alchemilla minima | Kew DNA bank | KF997287 | KF997456 | 14875 | VS7355 |
| 159 | Alchemilla glaucescens | Kew DNA bank | KF997343 | KF997355 | 14876 | VS7356 |
| 160 | Sium latifolium | Kew DNA bank | KF997429 |  | 14901 | VS7357 |
| 161 | Platanthera bifolia | Kew DNA bank | KF997482 | KF997458 | 16025 | VS7358 |
| 162 | Arabis scabra | Kew DNA bank | KF997382 |  | 16138 | VS7359 |
| 163 | Dianthus gratianopolitanus | Kew DNA bank | KF997505 |  | 16646 | VS7360 |
| 164 | Bunium bulbocastanum | Kew DNA bank | KF997400 |  | 17661 | VS7361 |
| 165 | Sibbaldia procumbens | Kew DNA bank | KF997360 |  | 17757 | VS7362 |
| 166 | Sagina nivalis | Kew DNA bank | KF997380 |  | 21004 | VS7363 |
| 167 | Salix arbuscula | Kew DNA bank | KF997495 | KF997418 | 34547 | VS7364 |
| 168 | Physospermum cornubiense | Kew DNA bank | KF997435 |  | 35673 | VS7365 |
| 169 | Leucojum aestivum | Kew silica samples | KF997319 |  | MCS130 | VS7383 |
| 170 | Euphorbia hyberna | Kew silica samples | KF997442 |  | MCS312 | VS7388 |
| 171 | Saxifraga spathularis | Kew silica samples | KF997357 |  | MCS317 | VS7389 |
| 172 | Seseli libanotis | Kew silica samples | KF997338 |  | MCS319 | VS7390 |
| 173 | Seriphidium maritimum | Kew silica samples | KF997353 |  | MCS323 | VS7391 |
| 174 | Corynephorus canescens | Kew silica samples | | KF997384 | MCS327 | VS7392 |

**Table S3**: Age calibration points used in divergence time estimation (Mya = millions of years)

| **Clade (Crown group)** | **Age constraint (mya)** | **References** |
| --- | --- | --- |
| Tracheophytes | 454 (max) | (Clarke, Warnock & Donoghue 2011) |
| Monilophytes | 354 (min) | (Bateman 1991) |
| Seed plants | 310 (min) | (Miller 1999) |
| Angiosperms | 140 (min) – 217 (max) | (Soltis *et al.* 2008; Smith, Beaulieu & Donoghue 2010) |
| Nymphaceae | 115 (min) | (Friis, Pedersen & Crane 2001) |
| Eudicots | 121 (fixed) | (Drinnan, Crane & Hoot 1994) |
| Curcibitales - Fagales | 84 (min) | (Wikström, Savolainen & Chase 2001) |

Bateman, R.M. (1991) Palaeobiological and phylogenetic implications of anatomically-preserved archaeocalamites from the Dinantian of Oxroad Bay and Loch Humphrey Burn, southern Scotland. *Palaeontographica Abteilung B Palaophytologie*, **223**, 1–59.

Clarke, J.T., Warnock, R.C.M. & Donoghue, P.C.J. (2011) Establishing a time-scale for plant evolution. *New Phytologist*, **192**, 266–301.

Drinnan, A.N., Crane, P.R. & Hoot, S.B. (1994) Patterns of floral evolution in the early diversification of non-magnoliid dicotyledons (eudicots). *Plant Systematics and Evolution*, **8**, 93–122 supp.

Friis, E.M., Pedersen, K.R. & Crane, P.R. (2001) Fossil evidence of water lilies (Nymphaeales) in the Early Cretaceous. *Nature*, **410**, 357–360.

Miller, C.N. (1999) Implications of fossil conifers for the phylogenetic relationships of living families. *The Botanical Review*, **65**, 239–277.

Smith, S.A., Beaulieu, J.M. & Donoghue, M.J. (2010) An uncorrelated relaxed-clock analysis suggests an earlier origin for flowering plants. *Proceedings of the National Academy of Sciences of the United States of America*, **107**, 5897–5902.

Soltis, D.E., Bell, C.D., Kim, S. & Soltis, P.S. (2008) Origin and Early Evolution of Angiosperms. *Annals of the New York Academy of Sciences*, **1133**, 3–25.

Wikström, N., Savolainen, V. & Chase, M.W. (2001) Evolution of the angiosperms: calibrating the family tree. *Proceedings of the Royal Society London B*, **268**, 2211–2220.

**Table S4**: Model averaging results for generalized linear models with invasiveness as a binary trait for the entire UK. Relative trait difference and PNND were calculated using the DAPHNE phylogeny. N = 346 alien species (259 non-invasive, 87 invasive). Coefficients are averages from the full set of candidate models. SE = standard error, CI = 95 % confidence intervals; relative variable importance is the sum of Akaike weights across all models that included that variable (Burnham & Anderson 2002); * = a parameter was considered significant if its 95% confidence interval (CI) of the parameter estimate does not include 0.

| **Variable** | **Model averaged coefficient** | **Adjusted**  **SE** | **Lower CI** | **Upper CI** | **Relative**  **Importance** |
| --- | --- | --- | --- | --- | --- |
| Ellenberg *F* | 0.306 | 0.094 | 0.123 | 0.490 | 0.92 * |
| Ellenberg *N* | 0.244 | 0.105 | 0.037 | 0.451 | 0.76 * |
| Ellenberg *L* | -0.228 | 0.117 | -0.457 | 0.001 | 0.50 |
| Ellenberg *N* (Difference) | 0.126 | 0.097 | -0.064 | 0.317 | 0.24 |
| Ellenberg *L* (Difference) | 0.164 | 0.132 | -0.094 | 0.422 | 0.24 |
| Clonality | 0.396 | 0.314 | -0.221 | 1.012 | 0.21 |
| Ellenberg *S* (Difference) | -0.207 | 0.198 | -0.595 | 0.180 | 0.17 |
| Log height | 0.084 | 0.108 | -0.128 | 0.296 | 0.17 |
| Alien group  (archeophyte / neophyte) | -0.256 | 0.307 | -0.858 | 0.345 | 0.15 |
| Ellenberg *R* (Difference) | 0.093 | 0.134 | -0.170 | 0.356 | 0.13 |
| Life form (difference) | 0.128 | 0.277 | -0.415 | 0.671 | 0.13 |
| Ellenberg *R* | -0.006 | 0.159 | -0.318 | 0.307 | 0.13 |
| PNND | 0.0004 | 0.002 | -0.004 | 0.005 | 0.12 |
| Log height (difference) | 0.051 | 0.191 | -0.323 | 0.424 | 0.12 |
| Ellenberg *S* | 0.022 | 0.307 | -0.580 | 0.624 | 0.12 |
| Clonality (Difference) | -0.042 | 0.307 | -0.643 | 0.560 | 0.12 |
| Ellenberg *F* (Difference) | 0.306 | 0.094 | -0.181 | 0.207 | 0.12 |
| Primary life form:  Bulbous geophytes  Non-bulbous geophytes  Hemicryptophytes  Hydrophyte  Phanerophyte  Nanophanerophyte  Therophyte | -1.891  -0.203  -0.196  2.524  0.290  0.405  -0.510 | 1.210  0.855  0.621  1.379  0.715  0.781  0.620 | -4.262  -1.879  -1.413  -0.180  -1.113  -1.125  -1.725 | 0.479  1.474  1.020  5.227  1.692  1.935  0.705 | 0.08 |
